# Chlorthalidone vs Hydrochlorothiazide and Kidney Outcomes in Patients With Hypertension: A Secondary Analysis of a Randomized Clinical Trial

**DOI:** 10.1001/jamanetworkopen.2024.49576

**Published:** 2024-12-10

**Authors:** Areef Ishani, Cynthia Hau, Srihari Raju, Jessica K. Wise, Peter A. Glassman, Addison A. Taylor, Ryan E. Ferguson, William C. Cushman, Sarah M. Leatherman

**Affiliations:** 1 Minneapolis Veterans Affairs (VA) Healthcare System, Minneapolis, Minnesota; 2 Department of Medicine, University of Minnesota, Minneapolis; 3 Cooperative Studies Program Coordinating Center, VA Boston Healthcare System, Boston, Massachusetts; 4 Department of Biostatistics, Boston University School of Public Health, Boston, Massachusetts; 5 Pharmacy Benefits Management Services, Department of Veterans Affairs, Washington, DC; 6 VA Greater Los Angeles Healthcare System, Los Angeles, California; 7 David Geffen School of Medicine at UCLA, Los Angeles, California; 8 Michael E. DeBakey VA Medical Center, Houston, Texas; 9 Department of Medicine, Baylor College of Medicine, Houston, Texas; 10 Department of Medicine, Boston University Chobanian and Avedisian School of Medicine, Boston, Massachusetts; 11 Medical Service, Memphis VA Medical Center, Memphis, Tennessee; 12 Department of Preventive Medicine, University of Tennessee Health Science Center, Memphis

## Abstract

**Importance:**

Hypertension is a risk factor for the development and progression of chronic kidney disease (CKD). It is unclear whether different thiazide diuretics have a differential impact on kidney outcomes.

**Objective:**

To compare kidney outcomes in patients with hypertension taking chlorthalidone and hydrochlorothiazide.

**Design, Setting, and Participants:**

This prespecified secondary analysis of the Diuretic Comparison Project, a randomized clinical trial comparing chlorthalidone and hydrochlorothiazide for the treatment of hypertension, was conducted between June 1, 2016, and June 1, 2022, through Veterans Affairs facilities nationwide. This analysis extended follow-up to December 31, 2023. Veterans 65 years or older with hypertension who were taking hydrochlorothiazide were included.

**Intervention:**

The Diuretic Comparison Project randomized 13 523 participants to continue hydrochlorothiazide or switch to chlorthalidone.

**Main Outcome and Measures:**

The main kidney outcome was CKD progression, defined as doubling of serum creatinine level from baseline, a terminal estimated glomerular filtration rate (eGFR) less than 15 mL/min, or dialysis initiation.

**Results:**

Analysis included 12 265 participants (90.7%) with a baseline and 1 or more follow-up creatinine measurements (median [IQR] age, 71 [69-75] years; 3.2% female and 96.8% male). The mean (SD) study duration was 3.9 (1.3) years. Chlorthalidone was not superior to hydrochlorothiazide at preventing kidney outcomes (369 of 6118 [6.0%] vs 396 of 6147 [6.4%]; hazard ratio [HR], 0.94; 95% CI, 0.81-1.08; *P* = .37). Similar results were observed when a 40% or greater reduction of eGFR was substituted for doubling of creatinine in the above outcome, as well as any of the components of the primary composite outcome. There was no difference in the incidence of CKD (961 of 4520 [21.3%] for chlorthalidone vs 939 of 4518 [20.8%] for hydrochlorothiazide; *P* = .59) or acute kidney injury requiring hospitalization (391 [6.4%] for chlorthalidone vs 379 [6.2%] for hydrochlorothiazide; *P* = .63) between groups. However, a statistically significant increased incidence of hypokalemia for chlorthalidone vs hydrochlorothiazide was observed (545 [8.9%] vs 426 [6.9%]; *P* < .001).

**Conclusions and Relevance:**

Chlorthalidone was not superior to hydrochlorothiazide for kidney outcomes but was associated with an increased risk for hypokalemia. Given these findings, clinicians should feel confident using either agent for the treatment of hypertension and kidney outcomes.

**Trial Registration:**

ClinicalTrials.gov Identifier: NCT02185417

## Key Points

**Question:**

In patients with hypertension, is chlorthalidone superior to hydrochlorothiazide at preventing kidney outcomes?

**Findings:**

In this secondary analysis of a randomized clinical trial including 12 265 patients aged 65 years or older with hypertension, chlorthalidone was not superior to hydrochlorothiazide in preventing kidney outcomes. There was an increased incidence of hypokalemia events in the chlorthalidone group compared with the hydrochlorothiazide group.

**Meaning:**

Both chlorthalidone and hydrochlorothiazide may be used for the treatment of hypertension and kidney outcomes.

## Teaser

This secondary analysis of a randomized clinical trial examines whether chlorthalidone is superior to hydrochlorothiazide in preventing kidney outcomes in patients with hypertension.

## Introduction

Chronic kidney disease (CKD) is a common condition, with 92% of individuals having concomitant hypertension. Individuals with CKD and hypertension have an increased risk of both cardiovascular disease and progression of kidney disease. Extracellular volume excess is considered a primary driver of hypertension and CKD.^1,2^ Hypertension is a major cause of kidney failure requiring treatment (KFRT) and is the second most common cause of KFRT in the US.^3^ Thiazide diuretics improve cardiovascular outcomes^4^; however, there is limited evidence regarding the influence of diuretics on progression of kidney disease or the development of end-stage kidney disease. Secondary analysis of ALLHAT (Antihypertensive and Lipid-Lowering Treatment to Prevent Heart Attack Trial) suggested that individuals randomized to chlorthalidone had an estimated glomerular filtration rate (eGFR) of 3 to 6 mL/min/1.73 m^2^ lower at year 4 compared with those randomized to amlodipine.^5^ However, there was no difference in the incidence of a 50% reduction in eGFR or development of KFRT. Long-term follow-up from ALLHAT demonstrated no difference in KFRT when comparing chlorthalidone with either the amlodipine or lisinopril groups.^6^

Although diuretics can improve cardiovascular outcomes in those with CKD, it is unclear whether all thiazide diuretics are similar regarding clinical outcomes. Additionally, thiazide diuretics have differing incidences of hypokalemia,^7^ which may increase the risk of both cardiovascular disease and progression of kidney disease. Many clinical trials^8,9^ evaluating cardiovascular outcomes have found that chlorthalidone is beneficial. Several observational studies^7,10,11^ comparing chlorthalidone with hydrochlorothiazide have suggested an increased risk of acute kidney injury and progression of kidney disease with chlorthalidone compared with hydrochlorothiazide.

The Diuretic Comparison Project (DCP) was a large, pragmatic randomized clinical trial comparing chlorthalidone with hydrochlorothiazide and demonstrated no difference between groups for major adverse cardiovascular events and noncancer death.^12^ Adverse kidney events were a prespecified secondary outcome in the DCP protocol, and we aimed to examine whether chlorthalidone was superior to hydrochlorothiazide at preventing kidney outcomes. Assessment of treatment effects on risks of exploratory kidney outcomes and adverse events (eg, hypokalemia and acute kidney injury requiring hospitalization) were also included in the current secondary analysis.

## Methods

### Study Design

The design and main results of the DCP have been previously reported.^12,13^ The trial was conducted between June 1, 2016, and June 1, 2022. In brief, the DCP was a multicenter, 2-arm, comparative-effectiveness, embedded, pragmatic, open-label trial that was performed in the Veterans Affairs (VA) population. The key eligibility criteria included age of 65 years or older, history of hypertension with a most recent clinic systolic blood pressure (SBP) of 120 mm Hg or higher, and active hydrochlorothiazide prescription of 25 or 50 mg/d. There were no exclusion criteria related to kidney function. The study intervention was embedded in each of the 72 participating health care systems. Primary care practitioners at the included facilities were identified and approached with an electronic consent through the electronic health record (EHR) system.^14^ Once a physician consented to participate, their panel of patients was electronically screened to identify those meeting the inclusion and exclusion criteria. Eligible participants were mailed study information and consented by telephone. Medical record review was performed by study nurses to verify the eligibility status before randomization. The study was approved by the VA Central Institutional Review Board and sponsored by the VA Cooperative Studies Program and was conducted following the Consolidated Standards of Reporting Trials (CONSORT) reporting guideline.^15^ The original trial protocol is in Supplement 1.

Patients were centrally randomized to continue their hydrochlorothiazide or switch to chlorthalidone at what were assumed to be pharmacologically comparable doses (eg, from 25 mg/d of hydrochlorothiazide to 12.5 mg/d of chlorthalidone). Study medications were filled by the local VA pharmacy as a usual care medication and were not labeled as research medications. Both patients and physicians were aware of the treatment assignment. After randomization, patients were considered usual care, and all treatment prescriptions (including study medications) and monitoring of hypertension were managed by the primary care physicians. EHR-based pharmacy dispensing data were used, indicating when participants switched study medication (hydrochlorothiazide was dispensed to patients in the chlorthalidone group or vice versa) or discontinued taking the randomized drug (>90-day gap in drug coverage). The study did not define a blood pressure target for the primary care physicians. The study required no additional clinical visits or data collection beyond usual care.

The DCP trial was performed between June 1, 2016, and June 1, 2022; for this prespecified secondary analysis, we included an additional 1.5 years of EHR-based follow-up until December 31, 2023. Patients who underwent randomization and had a baseline and 1 or more follow-up creatinine measures were included in this intention-to-treat analysis.

### Outcomes

Evaluation of kidney outcomes was a priori specified in the DCP protocol. The primary outcome was CKD progression, defined as doubling of serum creatinine level from baseline, a terminal eGFR of less than 15 mL/min, or dialysis initiation. Other exploratory kidney outcomes included (1) an alternative composite measure consisting of a 40% reduction in eGFR (a terminal eGFR<15 mL/min or dialysis initiation), (2) incidence of new CKD (defined as eGFR<60 mL/min) among those without CKD at baseline, and (3) evaluation of change in annual eGFR slope (estimated as the absolute change between baseline and last eGFR taken during the study divided by duration of the 2 measurements and multiplied by 365.25 days). We additionally evaluated the incidence of hypokalemia and hospitalization for acute kidney injury as safety outcomes. Finally, we evaluated the effect of the intervention on prespecified subgroups.

Drug fills from VA outpatient pharmacies were also examined, limiting to those of interest and prescribed during the study. Specifically evaluated for this article are pharmacy fills for angiotensin-converting enzyme inhibitors, angiotensin receptor blockers, spironolactone, eplerenone, loop diuretics, sodium glucose cotransporter 2 inhibitors, and potassium supplementation.

### Data Collection and Outcome Ascertainment

Baseline patient characteristics, including demographics, comorbidities, medication history, SBP, and laboratory data, were extracted from EHR and/or claims data. Race and ethnicity were self-identified by participants and are included to assess the generalizability of the trial. Racial categories were Black or African American (hereafter Black), White, other (including American Indian, Asian, Hawaiian, Pacific Islander, and multiracial), and unknown due to missing data. Ethnicity categories were Hispanic or Latino and non–Hispanic or Latino. Study outcomes were ascertained by analyses of laboratory and EHR data stored in a national repository (ie, the VA Corporate Data Warehouse). Data related to kidney outcomes were updated from the original DCP trial to include follow-up measures until December 31, 2023. A list of predefined diagnosis and procedure codes using the *International Statistical Classification of Diseases, Tenth Revision* classification were applied to flag events of interest (eTable 1 in Supplement 2).

### Statistical Analysis

The current analysis focused on assessing whether chlorthalidone was superior to hydrochlorothiazide at preventing kidney outcomes. Patient characteristics were reported as mean (SD) or median (IQR) for continuous variables with nongaussian distribution by the Shapiro-Wilk test. Discrete and categorical variables were presented as number (percentage). Overall changes in SBP and potassium levels with mean (SD) are presented graphically. Treatment effects on risks for hypokalemia and acute kidney injury requiring hospitalization were examined with the Fisher Exact Test, as well as the proportion of participants with incident CKD. A Mann-Whitney *U* test was applied to assess group differences on the overall change in mean eGFR slope.

A Cox proportional hazards regression model, stratified by VA health care systems and with hydrochlorothiazide as the reference group, was used to estimate hazard ratios (HRs) for the kidney outcomes, including each individual component. Analyses were further refined with adjustment of baseline factors (age, male sex, Black race, body mass index, rurality of residence, history of diabetes, history of heart failure, current smoking status, baseline SBP, baseline eGFR, and baseline use of angiotensin-converting enzyme inhibitors, angiotensin receptor blockers, mineralocorticoid receptor antagonists, and sodium-glucose cotransporter-2 inhibitors. The covariates were selected based on study protocol description and clinical relevance. Cox proportional hazards regression was performed to evaluate potential interactions between treatment assignment and the prespecified subgroups.

All analyses were performed using SAS software, version 9.4 (SAS Institute Inc) and followed the intention-to-treat principles. A 2-sided *P* < .05 was considered statistically significant.

## Results

The DCP randomized 13 523 participants, of whom 12 265 (90.7%) had baseline and at least 1 follow-up creatinine measurement (median [IQR] age, 71 [69-75] years; 96.8% male and 3.2% female; 15.0% Black, 77.6% White, 2.3% other race, and 5.1% unknown race) (Table 1; eFigure 1 in Supplement 2). Of these patients, 6118 were randomized to receive chlorthalidone, and 6147 received hydrochlorothiazide. The mean (SD) follow-up for this analysis was 3.9 (1.3) years compared with 2.4 (1.4) years for the main outcomes analysis.^12^ Baseline characteristics are presented in Table 1. There were generally no differences in characteristics between those randomized to chlorthalidone vs hydrochlorothiazide and when stratified by baseline level of kidney function (eTable 2 in Supplement 2). There was a very high incidence of diabetes at baseline (45.9%) along with other comorbid conditions. At baseline, the median (IQR) eGFR was 71.2 (59.0-85.0) mL/min/1.73 m^2^, median (IQR) SPB was 139 (131-152) mm Hg, CKD was present in 3227 patients (26.3%), and the use of kidney-protective medications did not differ between groups.

**Table 1.** Demographic and Baseline Characteristics

Characteristic	No. (%) of patients^a^		Standardized difference
	Chlorthalidone group (n = 6118)	Hydrochlorothiazide group (n = 6147)	
Age, median (IQR), y	71 (69-75)	71 (69-75)	0.01
BMI, median (IQR)	31.1 (27.8-35.0)	31.2 (27.9-35.2)	0.04
Sex			
Female	198 (3.2)	191 (3.1)	0.01
Male	5920 (96.8)	5956 (96.9)	
Race			
Black	915 (15.0)	922 (15.0)	0.01
White	4745 (77.6)	4773 (77.6)	
Other^b^	144 (2.4)	142 (2.3)	
Unknown due to missing data	314 (5.1)	310 (5.0)	
Ethnicity			
Hispanic or Latino	219 (3.6)	245 (4.0)	0.01
Non–Hispanic or Latino	5681 (92.9)	5693 (92.6)	
Unknown due to missing data	218 (3.6)	209 (3.4)	
Resided in rural areas^c^	2778 (45.4)	2783 (45.3)	0.01
Current smoker	1385 (22.6)	1301 (21.2)	0.03
Medical history			
Diabetes	2763 (45.2)	2862 (46.6)	0.04
Heart failure	488 (8.0)	487 (7.9)	0.003
Myocardial infarction	214 (3.5)	232 (3.8)	0.02
Stroke	494 (8.1)	445 (7.2)	0.03
Hydrochlorothiazide, 25 mg	5783 (94.5)	5810 (94.5)	0.002
Duration of prior hydrochlorothiazide use, median (IQR), y	9.0 (4.0-13.9)	8.8 (3.9-13.6)	0.02
SBP, median (IQR), mm Hg	139 (131-152)	139 (130-152)	0.01
Serum creatinine, median (IQR), mg/dL	1.1 (0.9-1.2)	1.1 (0.9-1.2)	0.04
Serum potassium, median (IQR), mEq/L	4.0 (3.8-4.3)	4.0 (3.8-4.3)	0.01
eGFR, median (IQR), mL/min/1.73 m^2^	71.6 (68.9-84.4)	70.8 (58.7-84.8)	0.02
CKD status			
No CKD	4520 (73.8)	4518 (73.5)	0.01
With CKD	1598 (26.1)	1629 (26.5)	
Stage 3a	1114 (18.2)	1120 (18.2)	
Stage 3b	349 (5.7)	361 (5.9)	
Stage 4	135 (2.2)	148 (2.4)	
Antihypertensive drug use			
Hydrochlorothiazide alone	792 (12.9)	760 (12.4)	0.03
Hydrochlorothiazide plus 1 additional blood pressure medication	2100 (34.3)	2066 (33.6)	
Hydrochlorothiazide plus 2 additional blood pressure medications	1989 (32.5)	2026 (33.0)	
Hydrochlorothiazide plus 3 additional blood pressure medications	984 (16.1)	1005 (16.3)	
Hydrochlorothiazide plus 4 additional blood pressure medications	253 (4.1)	290 (4.7)	
Other drug use			
Angiotensin-converting enzyme inhibitor or angiotensin receptor blocker	3974 (65.0)	4035 (65.6)	0.02
Loop diuretic	131 (2.1)	157 (2.6)	0.03
Sodium-glucose cotransporter-2 inhibitor	161 (2.6)	170 (2.8)	0.01
Spironolactone or eplerenone	769 (12.6)	784 (12.8)	0.007

Abbreviations: BMI, body mass index (calculated as weight in kilograms divided by height in meters squared); CKD, chronic kidney disease; eGFR, estimated glomerular filtration rate; SBP, systolic blood pressure.

SI conversion factors: To convert creatinine to micromoles per liter, multiply by 88.4; potassium to millimoles per liter, multiply by 1.

^a^ Unless otherwise indicated.

^b^ Other includes American Indian, Asian, Hawaiian, Pacific Islander, and multiracial.

^c^ Based on the Veterans Affairs urban, rural, or highly rural classification.

**Figure 1.**
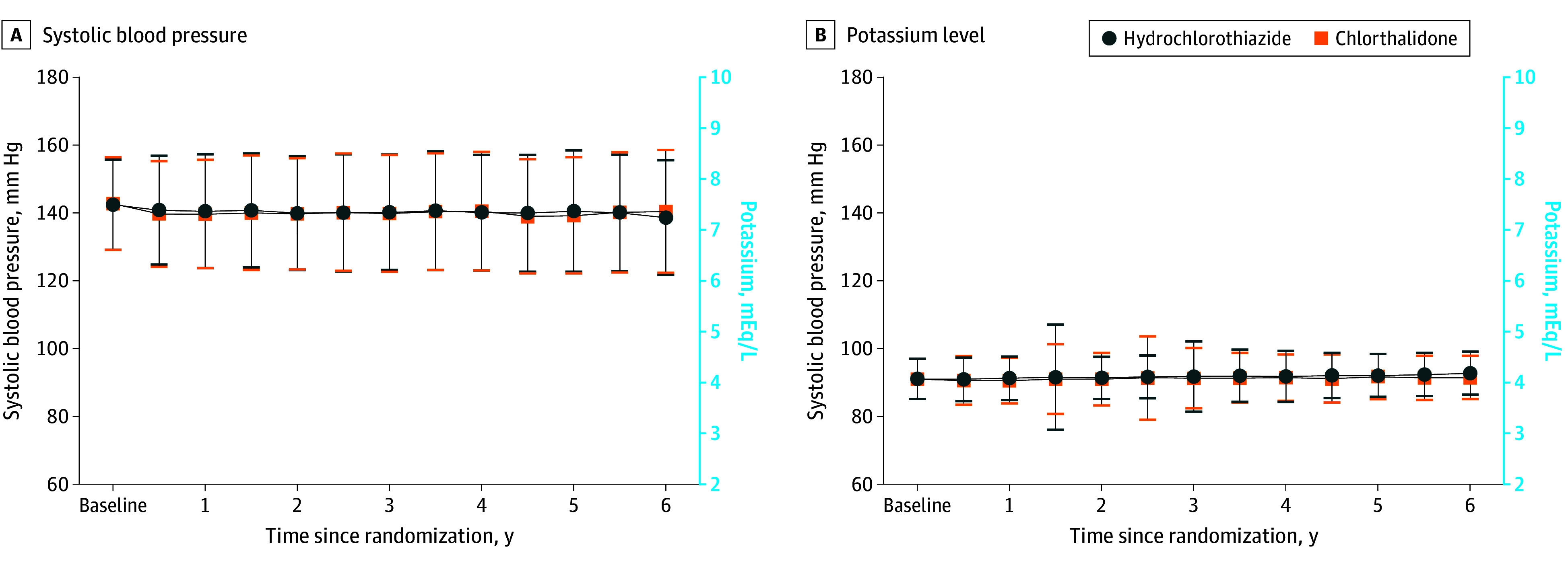
Systolic Blood Pressure and Potassium Level Error bars indicate SDs. To convert potassium to millimoles per liter, multiply by 1.

Throughout the trial, the median (IQR) doses used were 12.3 (8.1-13.4) mg/d for chlorthalidone and 23.0 (16.3-25.0) mg/d for hydrochlorothiazide, with a mean (SD) medication possession ratio of 79.7% (33.2%) for chlorthalidone and 79.4% (28.7%) for hydrochlorothiazide. Throughout the trial, 948 patients (15.5%) randomized to chlorthalidone were switched back to treatment with hydrochlorothiazide; 242 (3.9%) who had been randomized to continue treatment with hydrochlorothiazide were switched to chlorthalidone. SBP was similarly controlled between the chlorthalidone and hydrochlorothiazide groups both at baseline (mean [SD] baseline SBP, 142.7 [13.7] mm Hg for the chlorthalidone group vs 142.4 [13.3] mm Hg for the hydrochlorothiazide group) and at the mean (SD) duration of follow-up of 2.4 (1.4) years (140.2 [17.3] mm Hg for the chlorthalidone group vs 140.1 [17.3] mm Hg for the hydrochlorothiazide group) (Figure 1). The mean potassium level was also similar between the chlorthalidone and hydrochlorothiazide groups during the trial. Those in the chlorthalidone group were more likely to be taking potassium supplements during the trial (Figure 1). Use of other kidney-protective medications was similar over time by treatment group (eTable 3 in Supplement 2).

For the primary kidney outcome, chlorthalidone was not superior to hydrochlorothiazide in the incidence of doubling of serum creatinine level, an eGFR less than 15 mL/min, or dialysis initiation by treatment group (369 of 6118 [6.0%] vs 396 of 6147 [6.4%]; HR, 0.94; 95% CI, 0.81-1.08; *P* = .37) (Table 2; eFigure 2 in Supplement 2). These results were similar for individual components of the composite. Results were similar for a decrease in eGFR by 40%, an eGFR less than 15 mL/min/1.73 m^2^, or dialysis initiation by treatment group (778 [12.7%] in the chlorthalidone group vs 818 [13.3%] in the hydrochlorothiazide group; HR, 0.96; 95% CI, 0.87-1.06; *P* = .39) (Table 2; eFigure 3 in Supplement 2). Among those without CKD at baseline, incident CKD developed in 1900 of 9038 (21.0%) but was not different by randomized groups (961 of 4520 [21.3%] in the chlorthalidone group vs 939 of 4518 [20.8%] in the hydrochlorothiazide group; *P* = .59). Results did not change for the above outcomes when adjusted for baseline characteristics (Table 2). The overall mean slope of eGFR progression for the trial was −1.0 mL/min/1.73 m^2^ yearly with no mean (SD) difference between groups (−1.0 [7.9] in the chlorthalidone group vs −1.1 [8.9] in the hydrochlorothiazide group; *P* = .18).

**Table 2.** Primary and Exploratory Composite Kidney Outcomes by Treatment

Outcome	No. (%) of patients with outcome		Unadjusted^a^		Adjusted^b^	
	Chlorthalidone group (n = 6118)	Hydrochlorothiazide group (n = 6147)	HR (95% CI)	*P* value	HR (95% CI)	Log-rank *P* value
Primary composite outcome 1^c^	369 (6.0)	396 (6.4)	0.94 (0.81-1.08)	.37	0.96 (0.83-1.11)	.57
Exploratory composite outcome 2^d^	778 (12.7)	818 (13.3)	0.96 (0.87-1.06)	.39	0.98 (0.88-1.08)	.63
Outcome components						
Doubling of baseline serum creatinine level	331 (5.4)	353 (5.7)	0.94 (0.81-1.10)	.46	0.95 (0.82-1.11)	.52
eGFR decreased ≥40%	746 (12.2)	787 (12.8)	0.96 (0.86-1.06)	.37	0.97 (0.87-1.07)	.52
eGFR<15 mL/min/1.73 m^2^	255 (4.2)	255 (4.2)	1.01 (0.85-1.20)	.93	1.06 (0.89-1.27)	.50
Dialysis initiation	19 (0.3)	17 (0.3)	1.13 (0.59-2.17)	.72	1.13 (0.59-2.18)	.71

Abbreviations: eGFR, estimated glomerular filtration rate; HR, hazard ratio.

^a^ Included stratification of 72 participating Veterans Affairs health care systems.

^b^ Further adjusted for age, male sex, Black race, body mass index, rurality of residence, history of diabetes, history of heart failure, current smoking status, baseline systolic blood pressure, baseline eGFR, and baseline use of angiotensin-converting enzyme inhibitors, angiotensin receptor blockers, mineralocorticoid receptor antagonists, and sodium-glucose cotransporter-2 inhibitors.

^c^ Defined as the composite of doubling of serum creatinine from baseline, a terminal eGFR of less than 15 mL/min/1.73 m^2^, or development of kidney failure requiring treatment.

^d^ Defined as the composite of 40% reduction in eGFR, a terminal eGFR less than 15 mL/min/1.73 m^2^, or development of kidney failure requiring treatment.

**Figure 2.**
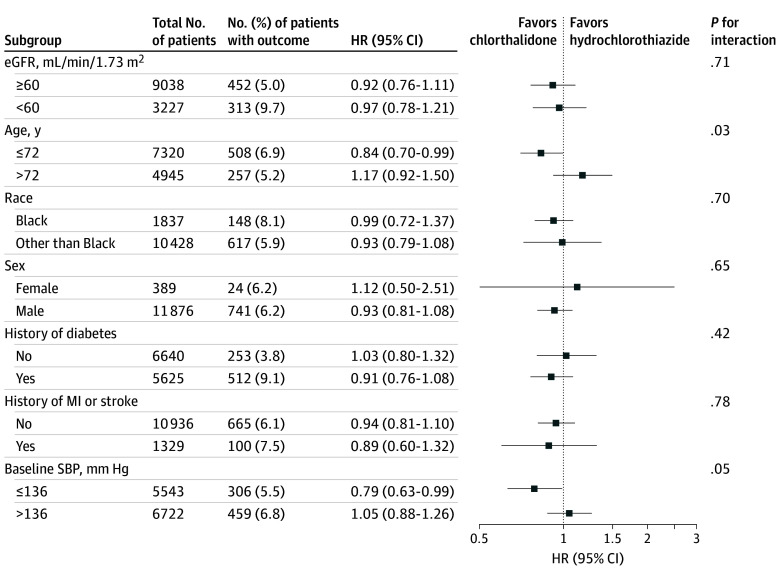
Effect of Intervention by Prespecified Subgroups on Primary Outcome The primary outcomes were unadjusted time to doubling of serum creatinine level, terminal estimated glomerular filtration rate (eGFR) less than 15 mL/min/1.73 m^2^, or dialysis initiation. HR indicates hazard ratio; MI, myocardial infarction; SBP, systolic blood pressure.

Figure 2 presents the adjusted primary kidney outcome by subgroups. Chlorthalidone was not superior to hydrochlorothiazide within subgroups defined by baseline CKD status, race, sex, the presence or absence of diabetes, myocardial infarction or stroke at baseline, or baseline mean SBP. There was a significant interaction by age and treatment group (chlorthalidone vs hydrochlorothiazide: age ≤72 years: HR, 0.84; 95% CI, 0.70-0.99; age >72 years: HR, 1.17; 95% CI, 0.92-1.50; *P* = .03 for interaction). Results were not different in adjusted analysis or in the secondary outcome analysis (eFigures 4 and 5 in Supplement 2).

### Adverse Events

Throughout the trial, the incidence of acute kidney injury requiring hospitalization was similar between groups (391 [6.4%] in the chlorthalidone group and 379 [6.2%] in the hydrochlorothiazide group; *P* = .63) (Table 3). Those randomized to the chlorthalidone group had an increased incidence of hypokalemia (potassium <3.1 mEq/L [to convert to millimoles per liter, multiply by 1]; 400 [6.5%] in the chlorthalidone group vs 293 [4.8%] in the hydrochlorothiazide group; *P* < .001). There was also a greater incidence of hospitalizations for hypokalemia in those randomized to chlorthalidone (213 [3.5%]) compared with hydrochlorothiazide (179 [2.9%]), although this difference was not significantly different (*P* = .07). When stratified by baseline CKD status, the difference in hypokalemia (potassium <3.1 mEq/dL) remained similar in those with and without CKD, with a difference of 1.8% and 1.3%, respectively. Only those without CKD had an increased risk of hospitalization for hypokalemia in the chlorthalidone group (161 [3.6%]) compared with the hydrochlorothiazide group (123 [2.7%]) (*P* = .03) (eTable 4 in Supplement 2).

**Table 3.** Other Kidney and Safety Outcomes by Treatment

Outcome	No. (%) of patients^a^		*P* value
	Chlorthalidone group (n = 6118)	Hydrochlorothiazide group (n = 6147)	
Other renal outcomes			
Yearly change in eGFR slope, mean (SD), mL/min/1.73 m^2^	−1.0 (7.9)	−1.1 (8.9)	.18^b^
Incident CKD	961/4520 (21.3)	939/4518 (20.8)	.59
Adverse outcomes			
Hospitalization for acute kidney injury	391 (6.4)	379 (6.2)	.63
Hypokalemia	545 (8.9)	426 (6.9)	<.001
Primary cause of hospitalization	213 (3.5)	178 (2.9)	.07
Potassium <3.1 mEq/L	400 (6.5)	293 (4.8)	<.001

Abbreviations: CKD, chronic kidney disease; eGFR, estimated glomerular filtration rate.

^a^ Unless otherwise indicated.

^b^ Based on Mann-Whitney *U* test; otherwise, based on Fisher exact tests.

## Discussion

Results from this analysis of the DCP demonstrated that chlorthalidone was not superior to hydrochlorothiazide in the incidence of the primary or secondary composite kidney outcome. Additionally, results were similar for the individual components of the primary composite outcome, including progression of kidney disease or dialysis initiation. Similarly, chlorthalidone was not superior to hydrochlorothiazide for the incidence of CKD or in the slope of eGFR progression. Subgroup analysis demonstrated similar effects across subgroups except by age, where the interaction between subgroup and treatment was significant. The clinical relevance of this interaction is uncertain. Finally, there was no difference in the incidence of acute kidney injury requiring hospitalization between groups. Individuals randomized to chlorthalidone had an overall increased risk of hypokalemia (potassium <3.1 mEq/dL) and an increased risk of hospitalization for hypokalemia compared with those receiving hydrochlorothiazide, particularly those without CKD at baseline.

Thiazide diuretics have been demonstrated to reduce blood pressure and the incidence of cardiovascular outcomes in those with hypertension. They are recommended as first-line agents by numerous hypertension guidelines for the general population^4,16^ and in those with CKD and no proteinuria.^17^ Historically, thiazide diuretics have been avoided in patients with CKD because they had been thought to be ineffective at lowering blood pressure because thiazides were believed not to reach the distal convoluted tubule in those with CKD. Rahman et al^5,18^ used ALLHAT to demonstrate that chlorthalidone reduced blood pressure to the same degree as other antihypertensive agents among those with advanced CKD (eGFR, 30-59 mL/min/1.73 m^2^) and that chlorthalidone similarly reduced major adverse cardiovascular event outcomes in this population compared with other antihypertensive agents. Similar results were observed in an extended follow-up of the ALLHAT cohort averaging 8.8 years.^6^ A meta-analysis^19^ by the Blood Pressure Lowering Treatment Trialists’ Collaboration suggested similar reductions in cardiovascular outcomes in those with an eGFR less than 65 mL/min/1.73 m^2^ compared with those with a greater eGFR and similar benefits regardless of class of antihypertensive agent used (including thiazides). More recently, the 2021 updated KDIGO (Kidney Disease: Improving Global Outcomes) guideline for the management of blood pressure in those with CKD recommended thiazide diuretics as first-line agents.^17^ The CLICK (Chlorthalidone in Chronic Kidney Disease) trial extended the evidence for chlorthalidone to those with stage 4 CKD by demonstrating an improvement in blood pressure at 12 weeks compared with placebo.^20^ However, chlorthalidone was associated with a greater reduction in eGFR and hypokalemia during the 12-week trial compared to placebo.

Although diuretics can reduce cardiovascular outcomes, there is concern regarding their effect on kidney function. Early epidemiologic and small observational studies have suggested greater decreases in eGFR and an increased incidence of kidney replacement therapy among thiazide users.^21,22,23^ Similar results were seen in ALLHAT in which participants randomized to chlorthalidone had lower year 4 eGFRs compared with those randomized to other antihypertensive agents but no difference in the incidence of KFRT.^5,6^ Recently, Fitzpatrick et al,^24^ using Kaiser observational data, suggested that in those with incident CKD, thiazide diuretic use was not associated with a composite kidney outcome (50% reduction in eGFR, a final eGFR<15 mL/min or KFRT) or with KFRT. To our knowledge, no randomized clinical trials have evaluated the effect of thiazide diuretics on long-term kidney outcomes.

Which thiazide diuretic to choose has also been controversial for cardiovascular disease as well as for kidney outcomes. A large propensity-matched cohort of incident diuretic users suggested that chlorthalidone use compared with hydrochlorothiazide use was associated with a greater risk of an eGFR decrease of 30% or greater but no difference in KFRT.^11^ Chlorthalidone was associated with an increased risk of hypokalemia compared with hydrochlorothiazide (HR, 1.70; 95% CI, 1.55-1.87), particularly in those with higher baseline eGFR values (*P* = .001 for interaction). These results contrast with this analysis from the DCP study, which demonstrated no difference in kidney outcomes, including doubling of serum creatinine level or initiation of dialysis, between chlorthalidone and hydrochlorothiazide groups. Additionally, the risk of incident CKD was similar between randomized groups. The DCP previously has demonstrated that cardiovascular events were also similar between groups in those with and without CKD at baseline.^12^ There was, however, an increased risk of hypokalemia among those randomized to chlorthalidone compared with hydrochlorothiazide, particularly in those without CKD at baseline.

### Strengths and Limitations

This analysis has many strengths, including the randomized design of the DCP, which allowed direct comparison of chlorthalidone with hydrochlorothiazide on the rate of progression of kidney disease, a prespecified analysis. We did not perform an a priori power analysis for this secondary outcome; however, the width of the CI suggests the study was not underpowered and rules out any clinically important differences between the 2 drugs (95% CI, 0.81-1.08). The DCP did not have any kidney exclusion criteria; however, most of those included had relatively preserved kidney function. The study was pragmatic and included individuals typically excluded from traditional exploratory trials, such as those living in rural and highly rural areas. Data for this analysis were from an extended follow-up from the DCP study, with a mean (SD) duration of follow-up of 3.9 (1.3) years compared with 2.4 (1.4) years for the primary results report. Finally, because of the pragmatic nature of the study, the results likely represent real clinical effects of these interventions.

There are also several weaknesses to this analysis. The trial only included individuals previously taking hydrochlorothiazide and likely limited adverse effects associated with thiazide diuretics (ie, hyponatremia). Similarly, individuals switched to chlorthalidone experienced a greater incidence of self-reported adverse events and switches back to hydrochlorothiazide. This was likely related to trial design, as those randomized to chlorthalidone started taking a new medication, whereas those randomized to hydrochlorothiazide continued taking a medication that they previously tolerated. The trial randomized patients to chlorthalidone or hydrochlorothiazide, and after randomization all follow-up was left to the primary care physician. There were no protocoled laboratory evaluations, including for kidney function or potassium levels. Results presented are from clinically evaluated laboratory values, resulting in varying numbers of laboratory values for each participant. This may have led to differential outcome ascertainment by group; however, it is likely that randomization removed both measured and unmeasured confounding for follow-up care. Dialysis initiation was ascertained using claims data, which have high specificity but lack sensitivity. Although some events may have been missed, it is likely that these missed events were balanced between groups given the randomized study design. Another limitation is that 95% of participants were initially taking the lower doses of hydrochlorothiazide (25 mg/d) or chlorthalidone (12.5 mg/d), so any potential harmful or beneficial kidney effects of the 50- or 25-mg doses, respectively, may have been missed.

## Conclusions

Overall results from this secondary analysis of the DCP demonstrate that chlorthalidone was not superior to hydrochlorothiazide for kidney outcomes at the doses observed. There was a greater tendency for a higher incidence of hypokalemia in those randomized to chlorthalidone compared with hydrochlorothiazide even in those with CKD, although the incidence and difference between the groups were small. Given these findings, clinicians should feel more confident in using either agent for the treatment of hypertension.

## Notes

Supplement 1.
Trial Protocol and Statistical Analysis

Supplement 2. **eFigure 1.** Consort Diagram of Patient Flow **eTable 1.** CPT Codes Used to Define Dialysis Procedures **eTable 2.** Demographic and Baseline Information by Treatment and eGFR Level **eFigure 2.** Primary Outcome: Time to Doubling of Serum Creatinine, Terminal eGFR<15 mL/min, or Dialysis Initiation **eFigure 3.** Exploratory Outcome: Time to a 40% Reduction in eGFR, Terminal eGFR<15 mL/min, or Dialysis Initiation **eTable 3.** Use of Selected Medications Over Time **eFigure 4.** Adjusted Subgroup Analysis: Time to Doubling of Serum Creatinine, Terminal eGFR<15 mL/min/1.73m^2^, or Dialysis Initiation **eFigure 5.** Unadjusted Subgroup Analysis: Time to >40% Reduction in eGFR, Terminal eGFR<15 mL/min/1.73m^2^, or Dialysis Initiation **eTable 4.** Safety Outcomes by Treatment and eGFR Level

Supplement 3.
Data Sharing Statement
