# Supplementary material for: Chlorthalidone vs Hydrochlorothiazide and Kidney Outcomes in Patients With Hypertension: A Secondary Analysis of a Randomized Clinical Trial
Source: JAMA Netw Open. 2024 Dec 10;7(12):e2449576. doi: 10.1001/jamanetworkopen.2024.49576 (PMC11632543; doi:10.1001/jamanetworkopen.2024.49576)
Supplement: Supplement 1. — Trial Protocol and Statistical Analysis [file jamanetwopen-e2449576-s001.pdf]

Department of Veterans Affairs Cooperative Studies Program

CSP#597: Diuretic Comparison Project (DCP)

a proposal from the Point of Care Program

Study Chair: Frank A. Lederle, MD

Study Co-Chair: William C. Cushman, MD

Protocol Version 2.1

12/21/2015

Diuretic Comparison Project (DCP)

Table of Contents

I. Abbreviations and Acronyms 3
II. Study Question 6
III. Background and Rationale 6
IV. The Point of Care Program within the VA’s Office of Research and Development 15
V. Relevance of our trial design to current needs of VA healthcare 18
VI. Study Design 20
VII. Study Population 20
VIII. Study Intervention 22
IX. Study Procedures 23
X. Outcome Measures 27
XI. Biostatistical Considerations 37
XII. Feasibility and Recruitment Plan 43
XIII. Data Collection and Data Sources 44
XIV. Data Management and Data Security Plans 45
XV. Human Subjects 48
XVI. Quality Control Procedures 51
XVII. Study Monitoring Plan 52
XVIII. Study Organization and Administration 55
XIX. Publications 58
XX. References 59

A. **Executive Summary**

Thiazide-type diuretics have been in use for more than 50 years and are considered in JNC-7 and VA guidelines to be the first-line treatment for hypertension. Of the more than 1 million veterans prescribed a thiazide-type diuretic each year, more than 95% receive hydrochlorothiazide, and fewer than 2.5% receive chlorthalidone. However, indirect evidence has been accumulating for many years that chlorthalidone may be more effective than hydrochlorothiazide at preventing cardiovascular events, by about 20% according to a recent network meta-analysis. Possible mechanisms for such an effect include longer duration of action, better nighttime blood pressure control, and pleiotropic effects of chlorthalidone. A randomized trial comparing the effect of the two drugs on cardiovascular outcomes has never been conducted, primarily for reasons of cost.

We are proposing a new type of efficient, less expensive randomized trial (termed a "clinically integrated" or "point of care" trial) to answer the question of whether chlorthalidone is more effective than hydrochlorothiazide at preventing cardiovascular outcomes in older patients with hypertension. Our primary outcome will be a composite consisting of: stroke, myocardial infarction, non-cancer death, urgent coronary revascularization, and hospitalization for acute congestive heart failure. We plan to enroll patients over age 65 years currently prescribed hydrochlorothiazide 25 or 50 mg daily with no recent systolic blood pressure below 120 mm Hg, and randomize them to either continue on hydrochlorothiazide or receive open-label chlorthalidone at suggested doses of 12.5 or 25 mg, respectively.

To have a 90% power with 2-sided $\alpha = 0.05$ to detect a 17.5% reduction in the expected 3% per year primary outcome occurrence rate in the hydrochlorothiazide group, we plan to randomize 13,500 patients (270 patients per center at 50 centers) over 3 years and follow them for a mean of 3 years, for a total study duration of 4.5 years.

The key feature of our design is that, instead of employing local investigators, we substitute centralized study processes and rely on usual primary care. Specifically, this involves: 1) identification of eligible patients using the VA electronic medical record system (EMR), 2) centralized recruitment and enrollment, involving permission from the patient's primary care provider, an 'opt-out' patient recruitment letter, and informed consent obtained by telephone, 3) centralized placement of notes and orders using the VA EMR, 4) all patient care including the study drug to be managed by the primary care provider, and 5) centralized passive collection of outcomes and process variables using the VA EMR, Medicare, and other national VA and non- VA databases.

I. Abbreviations and Acronyms

ACCOMPLISH	Avoiding Cardiovascular Events in Combination Therapy in Patients Living with Systolic Hypertension
ACCORD	Action to Control Cardiovascular Risk in Diabetes Trial
ACE	Angiotensin Converting Enzyme
ACEI	Angiotensin Converting Enzyme Inhibitors
ACME	Automated Classification of Medical Entities
AHA	American Heart Association
ALLHAT	Antihypertensive and Lipid-Lowering Therapy to Prevent Heart Attack Trial
ANBP2	Second Australian National Blood Pressure Study
ARB	Angiotensin Receptor Blocker
ARIC	Atherosclerosis Risk in Communities
BIRLS	Beneficiary Identification and Records Locator Subsystem database
CAC	Clinical Application Coordinator
CBOC	Community Based Outpatient Clinics
CDW	Corporate Data Warehouse
CFR	Code of Federal Regulations
CHD	Coronary Heart Disease
CHF	Congestive Heart Failure
CI	Confidence Interval
CPRS	VA Computerized Patient Record System
CSP	Cooperative Studies Program

126	CSPCC	Cooperative Studies Program Coordinating Center
127	CSRD	Clinical Sciences Research and Development
128	CSSEC	Clinical Sciences Scientific Evaluation Committee

29

CVD	Cardiovascular Disease
DM	Diabetes Mellitus
DMC	Data Monitoring Committee
DoD	US Department of Defense
ECG	Electrocardiography
EMR	Electronic Medical Record
EHR	Electronic Health Record
EXAMINE	EXamination of cArdiovascular outcoMes with alogliptIN versus standard of carE in patients with type 2 diabetes mellitus and acute coronary syndrome
FDA	US Food and Drug Administration
HCTZ	Hydrochlorothiazide
HDFP	Hypertension Detection and Follow-up Program
HF	Heart Failure
HIPAA	Health Insurance Portability and Accountability Act
HR	Hazard Ratio
ICD-9	International Classification of Disease, 9th edition.
ICD	Internal Cardiac Defibrillator
IRM	Information Resource Management
ISO	Information Security Officer
JNC-7	Seventh Report of the Joint Committee on Prevention, Detection, Evaluation and Treatment of High Blood Pressure
meq/L	Milli-equivalents per liter
mg	Milligram
MI	Myocardial Infarction
mmHG	Millimeters of Mercury
MRFIT	Multiple Risk Factor Intervention Trial

NHLBI	National Heart, Lung, Blood Institute
NIH	National Institutes of Health
PACT	Patient Aligned Care Team
PBM	VA Pharmacy Benefits Management
PCP	Primary Care Provider
PEACE Trial	Prevention of Events with Angiotensin-Converting Enzyme Inhibition PHI
	Protected Health Information
POC-CT	Point of Care Clinical Trial
PROBE	Prospective, Randomized, Open-label, Blinded-Endpoint Trial
SBP	Systolic Blood Pressure
SHEP	Systolic Hypertension in the Elderly Population Trial
SPRINT	Systolic Blood Pressure Intervention Trial
SOP	Standard Operating Procedure
TOMHS-T	Treatment of Mild Hypertension Study
TRACER	Thrombin-Receptor Antagonist Vorapaxar in Acute Coronary Syndromes
(TRA2°P)-TIMI 50	Thrombin Receptor Antagonist in Secondary Prevention of Atherothrombotic Ischemic Events Trial
UK NICE	United Kingdom National Institute for Health and Care Excellence
VA	US Department of Veterans Affairs
VAMC	VA Medical Center
VIREC	VA Information Resource Center
VISN	Veterans Integrated Service Network
WHO	World Health Organization

II. Study Question

Does treatment with chlorthalidone reduce cardiovascular outcomes compared with hydrochlorothiazide in older patients with hypertension?

III. Background and Rationale

A. Diuretics for hypertension

Hypertension is the most common primary diagnosis in America (1), and 3 of the 10 most commonly prescribed drugs in the US are antihypertensive agents (2). Thiazide-type diuretics are recommended as first line therapy by VA/DoD hypertension guidelines (3) and by the Seventh Report of the Joint Committee on Prevention, Detection, Evaluation, and Treatment of High Blood Pressure (JNC-7). JNC-7 noted that “diuretics have been virtually unsurpassed in preventing complications of hypertension” and as a result “should be used in drug treatment for most patients with uncomplicated hypertension” (4). In a network meta-analysis of 42 trials involving 192,478 patients randomized to active drug treatment for hypertension vs. placebo, low-dose diuretics (usually hydrochlorothiazide or the thiazide-type diuretic chlorthalidone) were “the most effective first-line treatment for preventing the occurrence of cardiovascular disease morbidity and mortality”(5).

B. Hydrochlorothiazide and chlorthalidone

Nearly all thiazide-type diuretic prescriptions in the U.S. are for hydrochlorothiazide. Hydrochlorothiazide is among the top 10 most commonly prescribed drugs in the US (2), with 135 million prescriptions written annually (6). In the VA national outpatient prescription database, of the more than 1 million veterans prescribed a thiazide-type diuretic each year from 2003-8, more than 95% received hydrochlorothiazide, and fewer than 2.5% received chlorthalidone (7). From our own search of the subsequent 3-year period 2009-11, 1.5 million veterans received hydrochlorothiazide from the VA and 50,000 received chlorthalidone.

The nearly universal use of hydrochlorothiazide has been attributed to a variety of factors, including 1) early aggressive marketing by its manufacturer (Merck) using “the largest pharmaceutical sales force in the world” (8), 2) its use in the early landmark VA hypertension treatment trials, 3) its early and frequent inclusion into combination pills, numbering at least 28 preparations (8), and 4) the ease of abbreviation to “HCTZ”, which may influence physician preference (9).

Both drugs have been approved by the FDA and in use for more than 50 years, have long been available as generics, and are included in the VA national formulary in the same drug class: USP code CV 701, thiazide-related diuretics. There is no patient characteristic that influences the choice between these two drugs - it is based solely on physician preference.

C. Drug dosages

Both drugs were once commonly used at doses of 100 mg per day and higher, but by 1990, concern over the lack of reduction in coronary events from blood pressure reduction by thiazides (despite proven efficacy for stroke), and further concern that this might be due to competing harms (particularly ventricular arrhythmias) from thiazide-induced metabolic abnormalities, led to recommendations to use lower doses of diuretics, including suggestions to use 12.5 mg of hydrochlorothiazide (10).

In the early 1990’s, publications of randomized dosing trials from the VA cooperative study group concluded that the doses of 25-50 mg of hydrochlorothiazide were nearly as effective as higher doses at controlling blood pressure with fewer adverse metabolic effects, favoring the use of 25 mg and a maximum dose of 50 mg (11,12). Several randomized dosing trials of chlorthalidone found 12.5 mg per day to be effective, and found 25 mg per day to be nearly as effective as higher doses with fewer adverse metabolic effects (13,14). Since then, doses of 12.5- 25 mg per day have been widely used (15) and rarely exceeded for both drugs, with higher doses accounting for only 7% of VA prescriptions in 2008 (7). However, several authors have pointed out that hydrochlorothiazide doses lower than 25 mg have not been shown to reduce cardiovascular outcomes and have performed poorly in randomized trials (6). As a result, JNC-8 recommends target doses of 25-50 mg per day for hydrochlorothiazide and 12.5-25 mg per day for chlorthalidone (16).

D. MRFIT – the first evidence that chlorthalidone may be more effective

Despite the near universal use of hydrochlorothiazide in the U.S., evidence has accumulated over the past 30 years suggesting that chlorthalidone may be more effective at reducing cardiovascular outcomes. The first indication was from the Multiple Risk Factor Intervention Trial (MRFIT), a large randomized trial of a multi-component ‘special intervention’ to prevent cardiovascular events in which either hydrochlorothiazide or chlorthalidone could be used as first-line treatment of hypertension in the special intervention arm. During the study, clinics that used hydrochlorothiazide were noted to have 44% more coronary heart disease deaths than those using chlorthalidone. In 1980, the MRFIT Policy Advisory Board changed the protocol, recommending chlorthalidone over hydrochlorothiazide for initial therapy, and lowered the maximum dose to 50 mg. Mortality in the former hydrochlorothiazide clinics subsequently dropped 28% (which could, of course, partially reflect regression to the mean). A recent analysis of the use of chlorthalidone and hydrochlorothiazide within MRFIT reported significantly fewer cardiovascular events with chlorthalidone, though the findings of this non-randomized comparison are confounded by large differences in dosage, randomized group, and lipid lowering (17). A separate non-randomized analysis of MRFIT data concluded that chlorthalidone was associated with less left ventricular hypertrophy than hydrochlorothiazide (18).

E. Indirect comparisons of randomized trial data

Because of the MRFIT observations, most subsequent NIH-funded blood pressure trials have used chlorthalidone, including the Hypertension Detection and Follow-up Program (HDFP), the Systolic Hypertension in the Elderly Program (SHEP), the Treatment of Mild Hypertension Study (TOMHS), and the Antihypertensive and Lipid-Lowering Treatment to Prevent Heart Attack Trial (ALLHAT), and in the ongoing Systolic Blood Pressure Intervention Trial (SPRINT), it is 'preferred'. The use of chlorthalidone in these trials and of hydrochlorothiazide in many other trials has enabled indirect comparisons of the effects of the two drugs against a third drug or class. Thus, hydrochlorothiazide resulted in worse outcomes than an ACE inhibitor (enalapril) in men in the Second Australian National Blood Pressure Study (ANBP2) (19), and worse outcomes than amlodipine (with all patients receiving benazepril) in the Avoiding Cardiovascular Events in Combination Therapy in Patients Living with Systolic Hypertension (ACCOMPLISH) trial (6), whereas in ALLHAT, chlorthalidone was found to be superior to an ACE inhibitor (lisinopril) or amlodipine "in preventing 1 or more major forms of CVD" (though there was no difference in the primary outcome) (20). These and other indirect comparisons have recently been combined into a focused network meta-analysis that estimated a 21% risk reduction ($p< .0001$) in cardiovascular events with chlorthalidone relative to hydrochlorothiazide that persisted (as 18%, $p=.024$) in an analysis adjusted for attained blood pressure (21).

F. Relative Potency

Several studies have found chlorthalidone to have about twice the potency of hydrochlorothiazide (22,23,24) and this is reflected in the VA Pharmacy Benefits Management (PBM) 2009 evidence review (25). Two indirect meta-analyses have reported similar conclusions. Ernst et al (26) found that at identical doses, chlorthalidone had a greater effect than hydrochlorothiazide on lowering blood pressure. Pederzan et al (27) considered the 2 drugs to have equivalent effects at equally potent doses, which they considered to be 3 to 1. Despite these indications of greater potency, in practice chlorthalidone is not used in lower doses than hydrochlorothiazide (7,28,29). This may reflect greater awareness of appropriate thiazide dosing by the small proportion of prescribers who use chlorthalidone, rather than widespread belief that the potencies are equivalent. There is essentially universal agreement among experts that chlorthalidone at 12.5 or 25 mg is equipotent to hydrochlorothiazide at 25 or 50 mg (respectively).

G. Possible differences between the two diuretics

No randomized trials have been conducted that directly compare clinical (e.g., cardiovascular) outcomes of chlorthalidone and hydrochlorothiazide, but several short-term randomized trials

have examined blood pressure and metabolic effects. Two recent short-term (10 and 12 weeks) randomized double-blind trials of 609 and 1071 patients (respectively) compared blood pressure

control and adverse effects when taking chlorthalidone 25 mg or hydrochlorothiazide 25 mg in patients also taking an angiotensin receptor blocker (ARB) (30,31). In both studies, chlorthalidone resulted in lower systolic blood pressure by at least 5 mmHg in clinic and, in the larger study, by 24 hour ambulatory recording.

Chlorthalidone is known to have a longer duration of action than hydrochlorothiazide. The elimination half-life of chlorthalidone is 50-60 hours compared with 9-10 hours for hydrochlorothiazide (32). Ernst et al (23) randomized 30 patients to either chlorthalidone 12.5mg/day, titrated to 25 mg/day or hydrochlorothiazide 25 mg/day, titrated to 50mg/day. After 8 weeks, there was no significant difference in office systolic blood pressure (SBP), but 24-h ambulatory SBP favored chlorthalidone over hydrochlorothiazide: -12.4 (± 1.8) vs. -7.4 (± 1.7) mmHg, respectively, P = 0.054, an effect primarily driven by the lower nighttime SBP for chlorthalidone compared with hydrochlorothiazide: -13.5 (± 1.9) and -6.4 (± 1.8) mmHg, respectively, P = 0.009. Of note in this regard, large observational studies have found nighttime blood pressure to be a better predictor of cardiovascular outcomes than daytime blood pressure (33,34).

A recent in vitro study reported that chlorthalidone reduced epinephrine-induced platelet aggregation and increased angiogenesis more than the thiazide bendroflumethiazide (35). There have been no reports of any related clinical effects (e.g., increased bleeding), but these mechanisms could help to explain differences between thiazides in reducing vascular events (36).

In summary, given the longer duration of action, better nighttime blood pressure control, and possible pleiotropic effects of chlorthalidone, it is possible that chlorthalidone and hydrochlorothiazide could have different long-term effects on cardiovascular outcomes even at doses that result in similar office blood pressures.

H. Drug costs

Although both drugs are inexpensive generic products, chlorthalidone costs the VA seven times as much as hydrochlorothiazide. According to VA Pharmacy Benefits Management (PBM), the cost to the VA for hydrochlorothiazide 50 mg is 1.6¢ per tablet and for chlorthalidone 25mg is 11¢ per tablet (with half doses costing half as much). Thus if the approximately 1 million VA patients using hydrochlorothiazide (nearly all on 12.5 or 25 mg) were switched over to chlorthalidone 12.5 mg at an additional cost of 5¢ per day, the total increased cost would be about $18 million per year.

I. Comparative metabolic effects

Thiazide diuretics have a variety of metabolic effects. They generally lower serum sodium (36) and potassium (27,38) levels and increase blood sugar (39,40) and uric acid (27,41) levels. In a small randomized trial (42), hydrochlorothiazide 50 mg per day lowered potassium by a mean of

0.44 meq/L more than placebo, regardless of whether potassium supplements or triamterene were added. Perhaps the best source of information on the effect of thiazides comes from ALLHAT, which randomized 33,357 patients with hypertension: 15,255 to chlorthalidone and 9000 each to amlodipine and lisinopril. Overall, chlorthalidone was considered superior to the other drugs in preventing cardiovascular events (20). The year-1 incidence of hypokalemia (<3.5 meq/L) was higher with chlorthalidone (12.9%) than with lisinopril (1.0%) or amlodipine (2.1%), but only 3.5% of the chlorthalidone group had a level <3.2 meq/L, and, in the chlorthalidone group, hypokalemia was associated with fewer cardiovascular outcomes than was normokalemia (38).

According to UpToDate (43): "The severity of the manifestations of hypokalemia tends to be proportionate to the degree and duration of the reduction in serum potassium. Symptoms generally do not become manifest until the serum potassium is below 3.0 meq/L, unless the serum potassium falls rapidly or the patient has a potentiating factor, such as a predisposition to arrhythmia due to the use of digitalis. Symptoms usually resolve with correction of the hypokalemia. … Muscle weakness usually does not occur at serum potassium concentrations above 2.5 meq/L if the hypokalemia develops slowly. … In addition to causing muscle weakness, severe potassium depletion (serum potassium less than 2.5 meq/L) can lead to muscle cramps, rhabdomyolysis, and myoglobinuria." In one study, occurrence of premature ventricular contractions was twice as common when serum potassium was below 3.0 meq/L (43).

In ALLHAT (39,40), chlorthalidone was also associated with more incident diabetes (defined as any fasting blood sugar >125 mg/dL) than were the other drugs (chlorthalidone: 14%, amlodipine: 11.1%, lisinopril: 9.5%). While overall, those with incident diabetes had more cardiovascular deaths, incident diabetes in the chlorthalidone group had lower cardiovascular deaths than incident diabetes in other groups, leading the ALLHAT investigators to conclude that "there is no conclusive or consistent evidence that this diuretic-associated increase in DM risk increases the risk of clinical events" (39), so "concerns regarding potential adverse diabetic effects associated with thiazide-type diuretic therapy should not inhibit its use" (40). Roush et al (44) recently reviewed this literature and concluded that "Chlorthalidone-induced diabetes mellitus (DM) is "chemical diabetes" rather than DM leading to cardiovascular pathology."

Also in ALLHAT, chlorthalidone did not increase the rate of development of either end-stage renal disease or of a 50% or greater decrease in glomerular filtration rate compared with lisinopril or amlodipine (45,46).

Sodium, uric acid, and gout were not followed in ALLHAT. Thiazides have been associated with hyponatremia in observational studies. In the Rotterdam study, about 50 of 3400 patients treated with thiazides over 6 years developed Na < 130 meq/L, 4.5 times as many as controls (36).

However, in SHEP, patients randomized to chlorthalidone did not differ from those receiving placebo in sodium levels after 1 year (47).

Similarly, in the observational Atherosclerosis Risk in Communities (ARIC) study, thiazides were associated with an increased risk of incident gout mediated by increased uric acid levels

(41). A recent systematic review concluded "There is a trend toward a higher risk for acute gouty arthritis attacks in patients on loop and thiazide diuretics, but the magnitude and independence is not consistent. Therefore, stopping these useful drugs in patients who develop gouty arthritis is not supported by the results of this review" (48). In HDFP, patients randomized to chlorthalidone had reduced mortality compared with usual care regardless of baseline uric acid level (49). In a matched sample comparison of national pharmacy records, new onset gout episodes occurred with similar frequency in the year following prescription for chlorthalidone or hydrochlorothiazide, despite the 2 drugs being used in equal milligram doses (50).

The above comparisons involving thiazide vs. no thiazide demonstrate effects that are minor and of uncertain clinical importance. In addition, two studies from university (51) and VA (52) settings changed 19 and 40 patients (respectively) on a stable dose of hydrochlorothiazide to an equal milligram dose of chlorthalidone. Both reported reduced blood pressure with no significant metabolic effects except for one instance of hypokalemia in the university study.

Changing from hydrochlorothiazide to a roughly equipotent half dose of chlorthalidone (the intervention proposed in this study) should result in even smaller effects. In the randomized study by Ernst et al (23), hypokalemia < 3.5 meq/L occurred in nearly identical proportions at 2:1 doses: 50% of patients on hydrochlorothiazide 25/50mg and 46% on chlorthalidone

12.5/25mg. Within the commonly used dosing range of 12.5-25 mg per day, potassium reduction was found to be equivalent for the 2 drugs in one meta-analysis (26), whereas the other found it to mirror the potency results, i.e. greater for chlorthalidone at equal milligram doses (27). In a Dutch population-based case-control study, hyponatremia was twice as common with chlorthalidone compared with hydrochlorothiazide at equal doses, but no difference was observed comparing 2:1 dosing (53). In the short-term randomized trials of 609 and 1071 patients that compared equal doses (25 mg) of the 2 drugs in patients also taking an angiotensin receptor blocker, hypokalemia was rare, occurring in 1-2% (30,31). Summarizing the metabolic data available for the 2 drugs, a recent review (54) concluded that "factors such as serum potassium, glucose, lipids, endothelial function, and oxidative status" "are either favorable to chlorthalidone or are neutral in arriving at a decision as to which drug is superior."

More recently, a population-based observational study from Ontario compared effects of starting treatment with chlorthalidone (10,384 patients) vs. hydrochlorothiazide (propensity matched sample of 19489 patients) with a mean follow-up of about one year (29). Patients treated with chlorthalidone received higher doses (despite its greater potency), and were less likely to also be treated with an ACEI or ARB (drugs that raise potassium levels). Chlorthalidone was associated with a small non-significant reduction in the composite cardiovascular outcome,

from 3.4 to 3.2 per 100 patient-years (adjusted HR 0.93, CI: 0.81-1.06). However, treatment with chlorthalidone was associated with significantly more hospitalizations with (not necessarily “for”) hypokalemia (0.69 vs 0.27 events per 100 patient-years, adjusted HR 3.06, CI: 2.04-4.58) and hyponatremia

(0.69 vs 0.49 events per 100 patient-years, adjusted HR 1.68, CI: 1.24-2.28). The authors included hospitalizations that listed electrolyte abnormalities as secondary diagnoses noted during hospitalizations for other reasons. Hypokalemia and hyponatremia were each recorded as a secondary outcome noted during hospitalization less than once per 100 patient-years. In response to a letter suggesting that the analysis should be restricted to hospitalizations "for" hypokalemia (as primary diagnosis), the authors responded that doing so would result in so few hospitalizations that "such an analysis would be severely underpowered" (55). So while it is not known whether chlorthalidone caused more hospitalizations "for" hypokalemia, it is clear that such hospitalizations were rare.

The Ontario study had the advantages of large size and direct comparison of the two drugs in a population. The principal disadvantages were the observational design and the very small (and therefore potentially quite different) proportion taking chlorthalidone. Incomplete adjustment for known confounders (e.g., for dose and co-treatment) or from unrecognized confounders in the treated populations could have influenced the findings, as noted in a letter by the ALLHAT investigators (56). For example, chlorthalidone is likely used more often by hypertension specialists who might have been more attentive to recording electrolyte abnormalities on discharge summaries. Review of US data indicates that chlorthalidone is used in patients with more severe co-morbidities than those given hydrochlorothiazide (57). The Ontario study nevertheless raises questions regarding the possible superiority of chlorthalidone. In the correspondence following its publication, both the ALLHAT investigators and the Ontario authors stress the need for a randomized trial, such as the one we are proposing, to resolve this uncertainty (55,56).

In summary, the metabolic effects of thiazides are minor and have little or no clinical effect. Evidence from a variety of studies indicates that substitution of an equipotent dose of chlorthalidone for hydrochlorothiazide can be expected to have no more metabolic effect than might occur if the patient remained on hydrochlorothiazide without the substitution. **This is the basis for our assertion that this substitution constitutes minimal risk**.

J. Expert views on the choice of drugs and the need for a randomized trial

The evidence summarized above is consistent with a substantial benefit from using chlorthalidone rather than hydrochlorothiazide, but is not compelling, as the Ontario study illustrates. Many of the studies describing a possible advantage of chlorthalidone were conducted by Ernst and colleagues at the University of Iowa (7,18,22,23,26,28), but those authors have stated that "we do not believe there is strong evidence to support the use of chlorthalidone over HCTZ" (22). Ernst and Marvin Moser (who pioneered thiazide use in the 1950's) wrote in 2009: "Whether hydrochlorothiazide and chlorthalidone are interchangeable in reducing the risk of cardiovascular events is questionable"(32). On the other hand, Messerli et al reviewed the literature and concluded that "there is no evidence showing that HCTZ in the dose

of 12.5-25 mg reduces myocardial infarction, stroke, or death” and argue that “if a thiazide-type diuretic is

indicated, either chlorthalidone or indapamide should be selected” (6). The 2011 UK National Institute for Health and Care Excellence (NICE) guidelines for hypertension (58) recommend “a thiazide like diuretic, such as chlorthalidone…, in preference to a conventional thiazide diuretic such as bendroflumethiazide or hydrochlorothiazide”, whereas the 2013 European Society guidelines dispute this and conclude that “no recommendation can be given to favor a particular diuretic agent” (59).

A recent review from investigators at the New Mexico VA (15) concludes: “The available evidence therefore supports both HCTZ and chlorthalidone as safe and effective drugs for treating hypertension. Although there are favorable trends both in terms of antihypertensive efficacy as well as clinical outcomes data with chlorthalidone compared with HCTZ, the results are not conclusive, and as such may not be enough to shift the treatment paradigm in favor of chlorthalidone, given the comfort level that most prescribers have with HCTZ. A head-to-head study looking at hard clinical outcomes, which may or may not ever be performed, may be the only way to resolve the ongoing debate as to which is the preferred thiazide for treating hypertension.” Floyd and Psaty noted in 2012 (60) that “In the area of pharmacological drug treatment for high blood pressure, the current question of primary interest is whether health outcomes associated with the use of hydrochlorothiazide and chlorthalidone may differ.

…Reliable and valid comparisons between hydrochlorothiazide and chlorthalidone will require a large, long-term clinical trial.”

As suggested by the New Mexico authors, it is extremely unlikely that a randomized clinical outcomes trial of hydrochlorothiazide vs. chlorthalidone will ever be undertaken except by the inexpensive methodology we are proposing. Neaton and Grimm, University of Minnesota Professors who participated in MRFIT, SHEP, and subsequent NIH hypertension trials, have been lobbying for such a trial since the end of SHEP more than 20 years ago. They have discussed the idea with the NHLBI project office numerous times over conference calls and had a meeting in Bethesda about this issue. According to Dr. Grimm, NHLBI project officers have maintained that they will only consider a 5 year proposal capped at $1.5 million a year. The Director of the NHLBI Cardiovascular division has recently written that “we can no longer afford to undertake randomized effectiveness trials that cost tens or hundreds of millions of dollars” (61). Apart from a trial design such as we are proposing, which may be unique to the VA system, these investigators found it impossible to design a study for an amount close to this budget. Richard Grimm concluded a 7/29/13 email to the principal proponent (FAL) with: “It looks like the last chance of getting a definitive answer for this incredibly important question is your VA proposal.”

K. Summary of evidence and potential impact of the proposed trial

Direct evidence shows chlorthalidone to be more potent and longer-lasting than hydrochlorothiazide, and indirect interventional evidence from the Roush network meta-analysis

(21) suggests that chlorthalidone may have a more beneficial effect on cardiovascular events,

whereas the large Ontario observational study (29) did not find a significant reduction in adverse cardiovascular events. In comparing these 2 methodologies, the International Society for Pharmacoeconomics and Outcomes Research Indirect Treatment Comparisons Good Research Practices Task Force concluded that “a network meta-analysis must be considered observational evidence, but is arguably less prone to confounding bias than an observational comparative (prospective) cohort study” (62).

Currently available data thus favor a likely substantial benefit from chlorthalidone compared with hydrochlorothiazide, but these data have had little effect on prescribing, which continues to overwhelmingly favor hydrochlorothiazide.

If cardiovascular events were reduced by even a small amount by chlorthalidone, the public health effect would be considerable because of the large number of patients who take diuretics. For the VA, it would likely justify the effort and cost of implementing a national policy to change drugs. However, the evidence is not yet persuasive enough to justify active measures directed at increasing chlorthalidone use. A randomized head-to-head comparison of the effectiveness of these two drugs at reducing cardiovascular events is clearly necessary to determine whether chlorthalidone is superior and if so, to justify efforts to change practice.

IV. The Point of Care Program within the VA's Office of Research and Development

A. Program Background

Medical decision making is informed by clinical trials and observational studies. While clinical trials are the gold standard in clinical research the high cost of conducting these studies combines with issues of generalizability to limit their contribution to changes in medical practice.

Observational studies are far less expensive and thus more numerous, addressing a broader scope of clinical issues. However, their primary failing is that the lack of randomization often leaves open the possibility of bias due to selection by indication, and residual confounding of results due to unobserved prognostic factors that influence treatment decisions.

Point of care (POC) randomization represents an intermediate strategy between these two approaches. The intent is to introduce the opportunity to randomize patients at decision points in clinical care where two or more alternatives are considered equivalent on average by the medical community (that is, clinical equipoise exists). Patients who agree to be randomly assigned to treatment options will become subjects in the clinical experiment.

POC randomization preserves the experimental quality of clinical trials without the cost of the clinical trial apparatus; recruitment and randomization is done at the point of care with minimal perturbation of work flow, and outcomes are assessed by automated extraction of data from the medical record. Reduction of the need for research staff interaction with potential subjects (limited to obtaining informed consent) greatly reduces cost and generates data that reflects the *effectiveness* (rather than efficacy) of experimental interventions in clinical care.

To be effective, the additional burden on patients and health care providers imposed by POC randomization must be minimal. The VA electronic medical record system (VistA) can be customized to identify, enroll and randomize patients and serve as the source of outcomes data and is thus well suited for such a trial design. Subject recruitment and enrollment will be accomplished by embedding processes within the VistA system through a series of dialog boxes.

As noted in a recent editorial (63), "With optimal use of EMRs, the administrative costs of a trial need not increase with the sample size; this decoupling of costs and size facilitates large, simple, and inexpensive trials that have the potential to transform health systems into entities that learn and continuously improve."

As the VA system continues to lead in the development of EMR that support increasingly sophisticated monitoring for outcome-based evaluation of care, we anticipate that the methods we test using POC randomization will have even greater scope for application. The

methods are also a natural fit for testing personalized and precision medicine strategies and for experimental comparative effectiveness research.

B. POC Pilot Study

The first implementation of POC randomization is a pilot study that is currently underway at 3 VAMC across 2 VISNs. The goal of the pilot was fivefold:

1. To test the feasibility of the method for modification of VistA/CPRS screens and the ability to randomize within the system;
2. To assess patient and provider acceptance of the new methodology;
3. To assess the regulatory acceptance of:
    a. Informed consent procedures;
    b. Safety monitoring;
    c. Ethical considerations;
4. To test the method for data extraction and passive collection of endpoint data using the EMR;
5. To apply Informatics techniques to refine and improve efficiencies in the identification of endpoints, etc.

The pilot is a comparison of two standard strategies of insulin administration for hospitalized patients and is designed as an open-label, randomized trial comparing sliding scale regular insulin (ssRI) to a weight based regimen for control of hyperglycemia in non-ICU inpatients. The strategy is to enroll patients into the study directly from the point of contact with clinical care within all inpatient facilities. All non-ICU patients who require in-hospital insulin therapy are eligible for this study. Clinicians decide at the time of care (through the VistA order entry screen for insulin) whether or not they will allow their patient to be contacted by POC study staff. If the treating clinician agrees, consenting patients are randomized to treatment arms and treated by their clinicians according to usual practices. All technical modifications necessary are executed within the "Clinical Alert" package. Consenting patients are then followed through VistA from randomization until 30 days post discharge. Comparisons of effectiveness will be formally conducted using length of hospital stay measured in days as our primary outcome measure.

The pilot has demonstrated the feasibility of the POC method as an alternative design to traditional trials. To date, 92% of eligible patients have accepted participation and have been consented into the pilot protocol. Additionally, 71% of all clinicians that are able to order medications have accepted randomization within the clinic and have referred their patients to the protocol. All patient data has been extracted from the EMR and has been validated with chart review by a qualified clinician. There have been no significant safety events and no findings from regulatory audits or the local institutional review boards.

C. The POC National Program

The goal of POC program is to deliver state of the art treatments to patients simultaneously with enrolling them as subjects to redefine that care. By institutionalizing a process of statistically sound and efficient learning, and by integrating that learning with automatic

implementation of best practice, the participating VA health care systems will accelerate improvements in the effectiveness of care for veterans. With this goal in mind, Point of Care Research has been designated as one of the Secretary’s (SECVA) Transformational Initiatives within the VA and

within the Office of Research and Development (ORD). Accordingly, this initiative has a national scope and an operating budget of approximately $10.2Million (exclusive of study budgets).

The MAVERIC CSPCC at the Massachusetts Veterans Epidemiology Research and Information Center (MAVERIC) has been tasked with leading the effort to implement POC randomization within the VA. To that end, MAVERIC’s mission is to build the infrastructure necessary to support POC research; to educate providers, veterans and investigators on the initiative; to build consensus for support of the initiative among the VA community as a whole; and to explore the ethical, scientific, and regulatory aspects of the POC method itself. In order to fulfill its mission, MAVERIC collaborates with national leaders in the fields of medical ethics and pragmatic clinical trials to produce scholarly works and it will conduct focus groups of veterans, veteran service organizations, and providers in order to develop a national educational campaign for all VA stakeholders.

V. Relevance of our trial design to current needs of VA healthcare

The most reliable way to learn if medical interventions provide more benefit than harm for our patients is through large randomized trials (64). However, large randomized trials that enroll thousands of patients can cost hundreds of millions of dollars, placing them out of range for most payers, including VA. As a result, many important questions remain unanswered.

We are proposing herein a 'clinically integrated' study design (65,66) that will incorporate and extend previously described VA 'Point of Care' Clinical Trial methodology (67,68). This is an efficient and inexpensive design, that will rely on a centralized processes involving mail, phone, and data extraction from the VA electronic medical records (EMR) , assisted by a designated study champion at each site (the site Liaison) and by local Clinical Application Coordinators (CACs). These methods will allow us to avoid having to employ study personnel at each site to manage patients and collect data. As a result, the infrastructure costs typically dedicated to these activities can be reinvested to answer other questions for the healthcare system. The goal, as Lauer and D'Agostino suggest, is "to design and conduct megatrials with what we have: bigger data and smaller budgets" (61).

The following quotes, excerpted from the 2011 Institute of Medicine report "Learning what works best - the nation's need for evidence on comparative effectiveness in health care"(69), provide support for our study objectives and design:

"A core objective for the nation is achieving the best health outcome for every patient. This objective simply cannot be accomplished until we have better evidence on which to base healthcare decisions"; "the most rapidly growing problem is that of our inability to produce the needed evidence in a timely fashion"; "Estimates of the proportion of medical care in the United States that is based on, or supported by, adequate evidence range widely. However, given concerns about the extent to which this information may be generalized, and the quality of the evidence which is used, some place this figure at well below half."; "Within the overall umbrella of clinical effectiveness research, the most practical need is for studies of comparative effectiveness, the comparison of one diagnostic or treatment option to one or more others."; "issues in need of additional systematic evaluation … include issues related to the comparative evaluation of different drugs within a single class"; "A learning healthcare system is one in which the clinical research paradigm depends more judiciously on the serial conduct of randomized controlled trials—important, but often too expensive, untimely, and of limited applicability—and draws more heavily on electronic health records (EHRs) to generate evidence as a natural by-product of the clinical experience."

More recently, the FDA has launched a Clinical Trials Transformation Initiative intended to promote and enable the conduct of larger, simpler, less expensive randomized trials using streamlining methodologies such as use of electronic health records (70).

In addition to addressing an important clinical question, our proposed trial is responsive to all these needs. This trial represents a new efficient methodology designed to provide reliable answers to practical clinical questions at a greatly reduced cost.

VI. Study Design

The study design is a multicenter clinically integrated (65) [or “point of care” (67,68)] prospective randomized open-label blinded-endpoint (PROBE) trial (71).

VII. Study Population

A. Inclusion Criteria

Eligible patients are those (including women and minorities) who:

1. Are over age 65 years

2. Are receiving hydrochlorothiazide from the VA pharmacy at a daily dose of 25 or 50 mg

3. Have a most recent SBP in CPRS ≥ 120 mm Hg, with no SBP < 120 mm Hg recorded in CPRS in the previous 90 days

B. Exclusion Criteria

Patients will be excluded if they are known to have any of the following:

1. Impaired decision-making capacity rendering the patient unable to provide informed consent (i.e., if there is any question during the nurse’s CPRS chart review that the individual does not have the ability to make an autonomous decision or the PCP declines permission to randomize)

2. Death expected within 6 months (inferred by PCP permission to randomize)

3. K< 3.1 meq/L (or 3.5 meq/L if on digoxin) in the past 90 days (assessed by CPRS review)

4. Na < 130 meq/L in the past 90 days (assessed by CPRS review)

5. Known to be enrolled in Medicare Part C (assessed through administrative data or on consent phone call). This exclusion will only be employed if we determine that we cannot obtain sufficient information from Part C data (see below under Rationale).

C. Rationale

We limit to age over 65 years to allow data collection through Medicare (which is only widely available starting at this age) [and will exclude known Part C enrollees (those enrolled in Health Maintenance Organizations), for whom usual encounter data is not available through Medicare if we determine that we cannot obtain sufficient information from Part C data].

We limit randomization to patients receiving hydrochlorothiazide because a) 95% of thiazide-type diuretic prescriptions are for hydrochlorothiazide, b) there is little evidence to suggest that chlorthalidone is inferior to hydrochlorothiazide, and c) the few PCPs who have deliberately chosen to use chlorthalidone over the much more commonly used hydrochlorothiazide are less likely to be willing to change drugs.

We limit to hydrochlorothiazide doses of 25 or 50 mg because lower doses may not be effective and cannot be easily converted to chlorthalidone (which is available only in 25mg tablets), and because higher doses are not recommended and are rarely used.

We limit to SBP ≥ 120 mmHg primarily to minimize risk from hypotension, and also to avoid enrolling patients whose blood pressure is already low enough that a potentially more effective drug would not be expected to add benefit (72, 73). The cutoff point of 120 mmHg was selected, in part, because we are using routine clinic blood pressures recorded in CPRS to determine study eligibility. These are obtained using a less rigorous measurement protocol than is normally used in randomized trials and that tends to overestimate blood pressure (74), in one study by a mean of 8 mmHg (75).

We will attempt to exclude patients in Part C as long as it appears that we will not be able to obtain adequate data. Part C data have not been available for research purposes in the past, but CMS plans to make the Healthcare Effectiveness Data and Information Set (HEDIS) for the years 2006-2011 available sometime in 2015. We plan to check with CMS periodically, and if, before or during enrollment, adequate Part C data appear to be accessible, we will discontinue the exclusion and attempt to enroll these patients.

VIII. Study Intervention

Participants will be randomly assigned to remain on their current dose of hydrochlorothiazide (25 or 50 mg), or to replace it with half that dose of chlorthalidone (12.5 or 25 mg, respectively), both changeable by the PCP. Chlorthalidone 12.5 mg will require tablet splitting, and a splitter will be mailed with the prescription. [Tablet splitting is a common procedure in VA pharmacy, sometimes including the splitting of hydrochlorothiazide 50 mg to get 25 mg. A recent review (76) concluded that “Tablet splitting does not seem to significantly affect clinical outcomes related to management of hypertension, cholesterol, or psychiatric disorders, nor influence overall patient adherence.”]

<u>Rationale for the default use of 1:2 chlorthalidone dose</u>: Because chlorthalidone is not used at lower doses than hydrochlorothiazide in practice (7,28,29), it could be argued that the most appropriate pragmatic comparison would be a 1:1 substitution. We selected the 1:2 dose default for 3 reasons: 1) as noted above, the use of similar doses in practice probably reflects greater awareness of appropriate thiazide dosing by the small proportion of prescribers who use chlorthalidone, rather than belief that the potencies are equivalent, 2) a 1:1 design would change the study question from assessment of a possible inherent difference between the two diuretics to an assessment whether low doses of hydrochlorothiazide are less effective, whereas our interest is in the former question , and 3) an equal dose of chlorthalidone would represent an intensification of diuretic therapy, resulting in increased effects on blood pressure and blood chemistries (51,52,53), whereas the 1:2 design results in virtually no change in blood pressure or metabolic effects (see Background and Rationale, parts F and I).

IX. Study Procedures

A. Procedures prior to patient enrollment

We will identify a "Liaison" at each site, usually a physician in primary care and/or hypertension management whose role will be limited to identifying key personnel at the site and introducing the study by giving presentations to local Primary Care Providers (PCPS), pharmacy, the Information Resource Management service (IRM), Clinical Application Coordinators (CACs), and Information Security Officers (ISOs) and subsequently referring all questions and comments to MAVERIC CSPCC. The Liaison will not be involved in recruitment or have patient interaction related to the study. Because we are introducing a new design and intend to evaluate its success, PCPs are also considered to be research subjects (see XI. Biostatistical Considerations, F. Secondary Data Analysis, 4. Primary Care Provider Metrics of Interest).

Our procedures are summarized in a flow diagram (**Appendix C**). Working through the VA national ISO structure and local CAC's and pharmacy personnel, we will obtain permission for MAVERIC CSPCC to have the necessary access to local CPRS systems to enter notes and post orders as View Alerts to PCPs and to collect the study data on enrolled patients. Research staff at the MAVERIC CSPCC will be responsible for communicating with local PCPs, coordinating and implementing all patient recruitment activities and completing the enrollment process including randomization and placing assigned treatment orders for signature by the PCP in CPRS. The MAVERIC CSPCC will partner with the Canandaigua VA Medical Center to conduct centralized calling and mailing activities, including contacting potential participants and obtaining informed consent by telephone.

We plan to roll out the study in blocks of sites. When a site starts up, the MAVERIC CSPCC will generate a local list of PCPs along with their eligible patients. We will also identify local PCP email groups and obtain the PCPs' VA Outlook email addresses. The MAVERIC CSPCC will send an introductory letter (by mail and email) to local PCPs signed by the co-Chairs providing information about the study (**Appendix A.1**). The site Liaison will present information about the study at primary care staff meetings and other forums (individual PACT meetings, video staff meetings for Community-Based Outpatient Clinics, etc.).

After these activities are completed, a View Alert 'testpatient' order will be sent to each PCP identified by the method above. The testpatient order will accompany a progress note containing the text of the Provider Information Sheet which will contain the elements of informed consent and detail the study procedures. (**Appendix B.3**) By signing the 'testpatient' view alert order, the PCP is agreeing to participate in the study as a research subject and allowing the recruitment letter (**Appendix A.2**) to be sent to eligible patients by the MAVERIC CSPCC. Alternatively, the PCP can "discontinue" the order, in which case that PCP will not be enrolled in the study and his/her patients will not be contacted.

B. Enrollment procedures

The procedures described below are summarized in a flowchart (**Appendix C**) and relevant CPRS screen shots are shown in **Appendix D**.

Using the VA electronic medical record, the MAVERIC CSPCC will identify eligible patients of PCPs who signed the testpatient order and will mail the study recruitment letter (**Appendix A.2**) with the Patient Information Sheet containing the consent transcript (**Appendix B.2**) to these patients. We will attempt to send the letter 3-4 months before the patient’s next planned appointment with the PCP to make it easier for the PCP to obtain any follow up on blood pressure and laboratory tests that the PCP might want. However, the VA Advanced Clinical Access/Recall system may prevent early detection of these appointments. The study recruitment letter and the Information Sheet with the transcript of the consent phone call that will be sent with the letter provide information about the study and the opportunity for the patient to opt out of future contact from the study.

If no ‘opt out’ reply is received by the coordinating center within 2 weeks after mailing the study recruitment letter, the patient will be contacted to confirm the details of consent. This will be done through the Canandaigua VAMC Call Center using a telephone line that displays the calling source as the VA on caller ID. We will make multiple attempts to reach the patient, only leaving a message on the first and last attempt. The U.S. National Health Interview Survey used a maximum of 15 call attempts and a 1994 study confirmed this number to be optimal (77). In 2 surveys conducted by Statistics Sweden, nonresponse rates stabilized at 15 and 20 maximum call attempts (78). In the Consumer Assessment of Health Plans Study, “most hard-to reach non-respondents were called 10 or more times” (79). A recent study at the University of Minnesota

(80) had an amendment approved by the UMN IRB in 2011 to increase the maximum number of call attempts from 6 to 12. Regarding left messages, Koepsell (81) “found that leaving a brief message about the study and promising a call-back improved the response rate by nearly 20 percentage points”. On the phone call, the information included in the letter will be reviewed and the patient’s informed consent will be sought using a pre-approved telephone script (**Appendix B.1**).

After telephone consent is obtained, a View Alert order will be sent to the PCP which, if signed by the PCP, will indicate permission to randomize that particular patient. Alternatively, if the PCP believes that patient should be excluded for any reason (e.g., incompetence or short life expectancy), the order can be “discontinued” and the patient will then receive a letter saying that their PCP declined their participation in the study (**Appendix A.3**).

C. Randomization

After the PCP signs the order allowing the patient to be randomized, the patient will be randomized by the MAVERIC CSPCC. Randomization will be to chlorthalidone or hydrochlorothiazide with equal probability. Randomized group will be open label, but allocation

will be concealed before randomization and irrevocable afterwards. All randomized patients will

be included in the analysis according to the intent-to-treat principle. Outcome assessment will be conducted by investigators blinded to treatment assignment.

We are thus proposing a "prospective randomized open-label blinded-endpoint" (PROBE) trial

(71). The rationale for our open-label design is several fold: 1) since there is no local coordinator, it is essential that the PCP manage the diuretic therapy, and we are concerned that this may not always occur if we use a blinded study drug, 2) there is no local investigator to assist with emergency unblinding, 3) we believe that keeping patients on open label therapy, being more familiar and straightforward from the patient's perspective, will enhance recruitment, thereby improving both feasibility and generalizability, 4) local pharmacy management of blinded drug would require a level of effort and local engagement in research incompatible with our streamlined study structure, 5) the expense of producing a study preparation that is identical for the two drugs, and then labeling and tracking each patient's therapy, would greatly increase cost, defeating the purpose of our highly efficient clinically integrated design. A recent meta- analysis found PROBE trials to be comparable to blinded trials in terms of assessing antihypertensive drug effect on blood pressure measurements, though clinical outcomes were not examined (71).

After randomization, a templated progress note placed by the MAVERIC CSPCC will appear as a View Alert to the PCP. The note will provide information on relative drug potency and provide the following reminders: 1) when resolving the view-alert orders, the PCP can accept the order by signing as is or change the dose, 2) the PCP can discontinue the order to continue their patient on his/her current diuretic, 3) the PCP may wish to order any desired laboratory tests or blood pressure checks, and 4) the PCP should manage the diuretic in the future according to the patient's needs.

Patients will be sent a letter informing them of their randomized group (**Appendix A.4**). Patients assigned to hydrochlorothiazide will simply remain on their current prescription. For patients randomized to chlorthalidone, the MAVERIC CSPCC will also generate View Alert orders to the PCP cancelling the hydrochlorothiazide prescription and replacing it with chlorthalidone.

Patients randomized to chlorthalidone will be instructed to discard their hydrochlorothiazide pills, and will be reimbursed for their co-pay on the discarded pills. The change from hydrochlorothiazide to chlorthalidone is a typical pharmacy action in usual care and will otherwise be handled with usual pharmacy procedures and information at that medical center.

Randomization will generate "health factors" that will serve to identify patients as enrolled in the study for tracking purposes in the VA EMR. In the unlikely event that the PCP, having very recently signed the "permission to randomize" view alert order, does not sign (i.e., "discontinues") the initial study drug order for chlorthalidone, the patient will still be analyzed

in the randomized group according to intent to treat. In these instances, we will contact the PCP to ask the reason for the discontinuation of the order.

We will monitor drug prescribing for study patients throughout the study. If the study drug is discontinued, a prescription is written for the other diuretic (cross-over), or the prescription is not refilled for 90 days after expected, a View Alert will be sent to remind the PCP about the study. If the situation persists after 2 weeks, we will review the chart to determine if the lapse was intentional and if so, try to determine the reason. If questions remain after the review, we may query the PCP.

X. Outcome Measures

A. Primary outcome

The primary outcome measure will be time to a major cardiovascular event, defined as a composite outcome comprised of the first occurrence (after randomization) of any of the following:

1. Stroke
2. Myocardial infarction
3. Urgent coronary revascularization (completed or attempted) because of unstable angina
4. Hospitalization for acute congestive heart failure
5. Non-cancer death

B. Secondary outcomes

Secondary outcomes will include:

1. All deaths
2. The composite outcome substituting all deaths for non-cancer deaths
3. "Possibly vascular deaths" defined as all deaths caused by vascular diseases, diabetes, external causes, and unknown causes
4. The composite outcome substituting "possibly vascular deaths" for non-cancer deaths
5. Each of the 5 components of the composite primary outcome
6. Any revascularization of any artery
7. Erectile dysfunction, defined as first prescription for PDE5 inhibitor or referral for ED

C. Process variables

1. Mean blood pressure during the study (outpatient clinics only; excludes inpatient, Emergency Department and Operating Room)
2. Time to discontinuation of the randomly assigned diuretic (defined as discontinue order or no prescription for ≥ 6 months at last observation during study period)

3. Mean compliance with study drug using “Medication Possession Ratio” (82) (used by VA Pharmacy Benefits Management Services)

4. Other antihypertensive drug use

D. Tertiary Outcomes

1. Hospitalization for primary diagnosis of hypokalemia, hyponatremia, or renal failure

2. Renal failure. Defined as dialysis, vascular access for dialysis, or renal transplant. (Doubling of serum creatinine from baseline will also be recorded and reported in the final analyses).

3. Other recorded hypokalemia (< 3.1 meq/L), or hyponatremia (< 130 meq/L)

4. New diabetes, defined as first use of a medication for diabetes

5. Acute gout episodes

6. New allergic reaction to thiazide-type diuretic, defined as new entry in Allergies/Adverse reaction (ART) database

E. Rationale for elements of composite primary outcome

The elements of the composite primary outcome are intended to represent the clinically important effects of diuretic therapy for hypertension. Myocardial infarction and stroke are traditional outcomes for cardiovascular clinical trials and will not be further justified here. Myocardial infarction is defined as the third universal definition (83). Stroke is defined as a neurological deficit of cerebrovascular cause that persists beyond 24 hours or is interrupted by death within 24 hours, including ischemic and hemorrhagic strokes (84).

<u>Urgent coronary revascularization</u>. Urgent coronary revascularization is an important outcome because these events accurately reflect both the progression of underlying atherosclerotic vascular disease and represent an important contributor to cardiovascular morbidity and healthcare costs. Disease progression that formerly resulted in myocardial infarction now frequently results in revascularization that aborts the infarction. Furthermore, because of increasingly sensitive troponin assays, many patients having urgent coronary revascularization attributed to unstable angina actually have mild troponin positivity and would be classified as myocardial infarctions in a classic trial design with in-depth outcome review (Christopher P. Cannon, MD, personal communication, Aug 2013). Including urgent coronary revascularization in our study will prevent us from missing these infarctions. Urgent coronary revascularization is more restrictive than the revascularization outcome usually reported in previous trials and selects for the most clinically relevant events. For example, in the PEACE trial of patients with stable coronary artery disease and normal ejection fraction (85), “all coronary

revascularizations” were more frequent than all the other events combined that make up our composite. On the other hand,

in the (TRA2°P)-TIMI 50 trial of an antiplatelet agent in patients with vascular disease (86), urgent coronary revascularization because of unstable angina was less frequent than myocardial infarction and similar in frequency to cardiovascular death or stroke, and increased the composite rate in the placebo group from 10.5% for those 3 outcomes to 12.4% for those 3 outcomes plus urgent revascularization.

Reductions in urgent coronary revascularization represent an important contribution to the overall effectiveness of therapy. The observed reductions in urgent revascularizations in randomized trials are concordant with changes in other major outcomes. In (TRA2°P)-TIMI 50 (87), the hazard ratio for the primary composite outcome of cardiovascular death, myocardial infarction, or stroke was 0.87, and for urgent coronary revascularization was 0.88. Urgent coronary revascularization has also been used in TRACER (82) and is being used in EXAMINE (88). CSSEC recently approved a broader revascularization outcome for VA CSP #593, the VA Fenofibrate Intervention Trial (VA-FIT) (for which the indications included stable angina with a >50% target lesion).

In summary, urgent coronary revascularization is a significant contributor to cardiovascular morbidity and health care costs, its inclusion will allow assessment of the full impact of therapy, and its relative contribution to overall endpoints is not anticipated to be disproportionate to that of other study outcomes. Evaluation of urgent coronary revascularization by review of EMR is straightforward, as discussed below.

Hospitalization for acute congestive heart failure. Hospitalization for acute congestive heart failure is another important component of our primary outcome. It will, in most instances, represent a new diagnosis of heart failure because most patients with established heart failure are maintained on a loop diuretic such as furosemide and are not treated with hydrochlorothiazide and thus would not be enrolled in our study. We will seek to identify clinical exacerbations of symptoms (e.g., not hospitalization for ICD placement), which should be associated with intensification of treatment, which we will assess in our algorithm.

Heart failure is a major public health problem with a profound impact on prognosis and also on costs, and is the most frequent cause of hospitalization among people older than 65 years (89). Its impact is summarized in the AHA 2013 update (90): "HF incidence approaches 10 per 1000 population after 65 years of age", …"approximately 50% of people diagnosed with HF will die within 5 years", …"One in 9 deaths has HF mentioned on the death certificate", …"In 2009, HF any-mention mortality was 274 601" and "HF was the underlying cause in 56 410 of those deaths".

Heart failure and stroke are the two major cardiovascular outcomes most related to hypertension and most benefited by treatment of hypertension. Seventy-five percent of heart

failure cases have antecedent hypertension (90). The Framingham Heart Study (91) found the highest risk ratio from hypertension to be for heart failure in men (Figure 1).

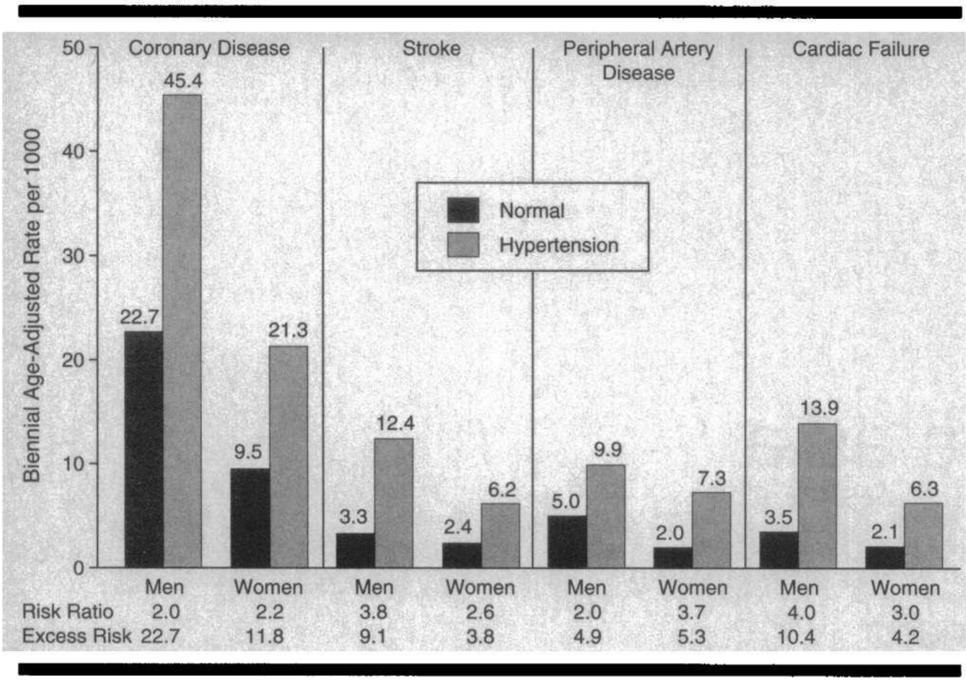

Figure 1. A prospective analysis of the 36-year follow-up data from the Framingham Heart Study (91) demonstrates that hypertension (blood pressure ≥ 140/90 mmHg) predisposes to all major atherosclerotic cardiovascular disease outcomes, but the largest risk ratios are for cardiac failure and stroke in men.

Diuretic-based treatment arms in outcome trials have reduced HF by an average of 50% over placebo (compared with 30-40% reduction for stroke), and more effectively than calcium channel blockers and ACE inhibitors in comparative trials (Figure 2) (5). In ALLHAT (89), the alpha blocker doxazosin, the calcium channel blocker amlodipine, and the ACE inhibitor lisinopril were associated with 80%, 38% and 19% higher risk of heart failure compared with chlorthalidone. Therefore, heart failure is likely to be one of the most sensitive outcomes for detecting a true difference in reducing cardiovascular disease between the two diuretics in our study.

Figure 2. Network Meta-analysis of First-Line Treatment Strategies in Randomized Controlled Clinical Trials in Hypertension

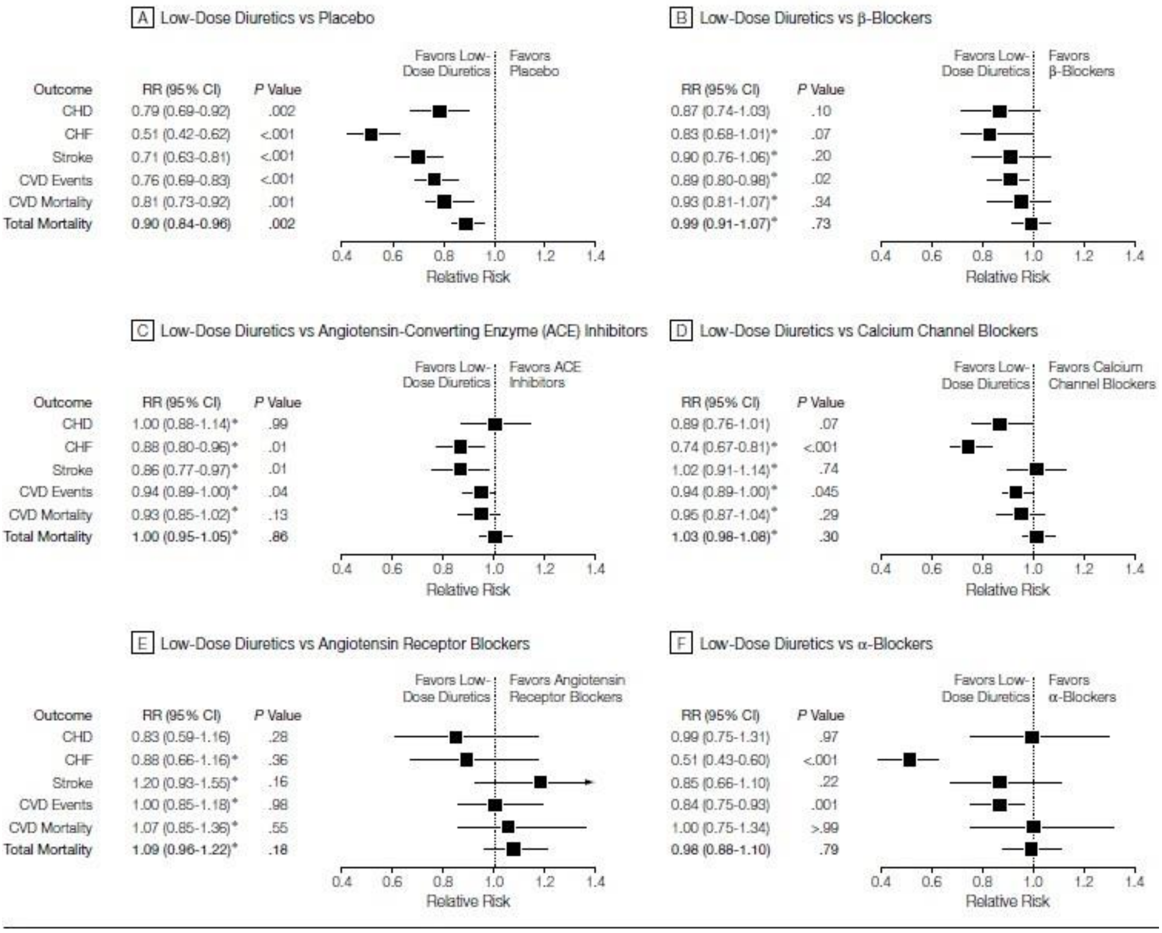

Figure 2. From a network meta-analysis of hypertension trials (5). Heart failure (CHF) can be seen to be a particularly effective outcome for discriminating between diuretics and other drugs. “CVD Events” include CHD, CHF, stroke and CVD deaths.

Hospitalized heart failure is not disproportionately common compared with our other outcomes. In the principal ALLHAT report (20), there were 885 non-fatal MIs, 724 hospitalized or fatal heart failure events, and 675 strokes. Hospitalized heart failure events in ALLHAT attracted close scrutiny because they were reported by site investigators and not adjudicated. The authors undertook a separate study in which they successfully validated these events (89), and from which they concluded “Heart failure proved to be a common outcome in ALLHAT and one that was affected differentially by the randomized treatment assignments. In addition, patients who developed HF had significantly poorer survival than those who did not. Whether this poor prognosis can be altered presents an important clinical and public health question.

Thus, *in planning future hypertension treatment trials* (and perhaps also treatment trials in other

populations at high risk of HF, such as diabetics and survivors of acute coronary syndromes) *serious consideration should be given to including HF in the primary end point*, along with death, MI, and stroke” (italics added).

Heart failure is reasonably well identified using discharge diagnoses. In a population-based study of 4537 cases of heart failure from Olmsted County MN, ICD-9 code 428 constituted 80% of heart failure codes, and 82% of the cases coded as 428 met Framingham criteria for heart failure when records were reviewed by experienced abstractors (92).

Identification of clinically important episodes can be improved by looking for evidence of treatment intensification. The draft report of the FDA expert panel on Cardiovascular Endpoints in Clinical Trials (93) recommends inclusion of the following criteria for determining a heart failure hospitalization event: “The patient receives initiation or intensification of treatment specifically for HF, including at least one of the following: a. augmentation in oral diuretic therapy, b. intravenous diuretic, inotrope, or vasodilator therapy, or c. Mechanical or surgical intervention, such as i. mechanical circulatory support (e.g., intra-aortic balloon pump, ventricular assist device) or ii. mechanical fluid removal (e.g., ultrafiltration, hemofiltration, dialysis).” We will employ these criteria and others, such as exclusion of admissions for ICD placement, in our algorithms to maximize the likelihood that the events we capture are clinically relevant.

<u>Non-cancer deaths</u>. We include non-cancer deaths in our primary composite outcome as a compromise between total mortality, which includes many irrelevant events that dilute the effect of the intervention, and cardiovascular mortality, which is less accurately distinguished on death certificates and may miss relevant deaths. We exclude cancer deaths because they are numerous, relatively accurately identified on death certificates (94,95,96), and believed to be unrelated to diuretic use, so excluding them reduces “noise” relative to expected effects of the intervention. In the Physicians’ Health Study (95), cancer deaths in patients older than 65 years were identified from death certificates using standard nosology protocols and the Automated Classification of Medical Entities (ACME) Decision Tables with specificity well over 99% compared with an adjudication committee, meaning that the deaths excluded from our primary outcome will almost certainly be due to cancer.

Prieto-Merino and colleagues (97) note: “If outcomes that are causally related to the trial treatment are combined with those that are not, the estimate of the treatment effect is diluted towards the null and we may fail to identify potentially important benefits or harms…. Because few treatments will be causally related to all causes of death, all-cause mortality is a composite outcome that combines causally related causes of death with those unrelated to the treatment.” We did not restrict to cardiovascular deaths because some other deaths may be relevant, such as accidents due to syncope from hypotension and deaths from unknown

causes (which could have been cardiovascular), and other deaths (e.g., pneumonia, COPD) that are not accurately

distinguished from cardiovascular deaths by death certificate diagnoses. Competing risk from cancer deaths will be considered in a secondary analysis.

F. Ascertainment of Outcome Data

Data collection will be via electronic medical records and administrative data. We will use both VA and CMS data. Assessment of outcomes and relevant data elements as well as adverse event is by passive collection of data in electronic health records; the study will not attempt to generate any additional tests or procedures. All outcome processing will be conducted by investigators at the MAVERIC CSPCC unaware of treatment group. Using a 150 chart review confined to VA data we have determined that we can identify the data elements we need to assess each of the components of the primary outcome. In this section we describe our approach to ascertainment and assessment of these outcomes.

The Boston MAVERIC Center has 15 years of experience in ascertainment and assessment of cardiovascular outcomes using the VA electronic health record (EHR) and CMS data. We will build on this extensive experience to refine a specific procedure for doing the same in this trial in order to accurately identify and assess outcomes. The process involves several clearly defined steps. First, we develop a method to screen the electronic medical records of all participants on a periodic basis (every 2 weeks) for potential cases. Second, algorithms are developed to collect and analyze data elements that are used to confirm or refute the potential cases. The algorithms are based on standard criteria used in clinical trials, and are applied to potential cases to determine if the case is confirmed, disconfirmed or deemed indeterminate. Third, indeterminate cases will undergo manual adjudication review. We expect to be left with a relatively small number of events that cannot be resolved by the algorithm, but all of these will be referred for adjudication to an outcomes committee unaware of treatment group. This will greatly lessen the workload for manual adjudication. All patients will be followed until death or the end of the study (even if the primary outcome is determined to have occurred) to collect secondary outcomes including death.

The development of the screening process and the algorithms for confirming cases follows a systematic approach that we have used at MAVERIC for a number of years. The screening approach is used to identify all potential cases using key elements in the EHR. The screening tool varies for each outcome. Algorithms for cases are constructed using “gold standard” cases identified by manual review and data elements used in criteria for confirmation obtained from the medical record, such as cardiac enzymes for MI. Once the algorithm for a specific case is defined, the algorithms accuracy is checked by manual review of cases identified by the algorithm and adjudicated in this way.

A pilot study using VA EHR was conducted to determine the availability of potential cases and core data elements that would be needed to develop algorithms to assess the primary

outcomes. A total of 150 medical records were reviewed, 30 in each category for stroke, myocardial

infarction and urgent coronary revascularization and 60 records of patients with a diagnosis of acute congestive heart failure. A medical record abstraction form for each outcome was developed based on standard diagnostic definitions that included information on symptom presentation, physical findings, critical laboratory values, radiographic or imaging findings, electrocardiographic results, hemodynamic data and administration of medications and therapeutic interventions (84, 98) During this study these data will be augmented with CMS data for all participants. Below we describe the approach to ascertainment of potential cases and the assessment of each.

Deaths: Death ascertainment from combined VA databases and Social Security has been shown to have comparable accuracy to the National Death Index and is more timely (99). We will collect data on deaths from combined VA databases (e.g., BIRLS, Medical SAS Inpatient), Medicare Vital Status, Social Security Administration Death Master File and the National Death Index.

The assigned cause of death will be determined by nosology protocols based on death certificate information obtained from the National and Social Security Death Indices using the International Classification of Diseases (ICD) in conjunction with the Automated Classification of Medical Entities (ACME) Decision Tables. ACME automates the underlying cause-of-death coding rules. The input to ACME is the multiple cause-of-death codes (ICD) assigned to each entity (e.g., disease condition, accident, or injury) listed on cause-of-death certifications, preserving the location and order as reported by the certifier. ACME then applies the World Health Organization (WHO) rules to the ICD codes and selects an underlying cause of death. This method has been shown to be accurate for general categories like cancer or vascular death (95).

Myocardial Infarction: For myocardial infarction, hospitalization ICD codes (410) as well as procedure and medications codes, such as lytic therapy, will be used to search for potential cases. MI codes labeled as “length of stay” diagnoses or labeled as “admitting” or “other” diagnoses will be considered as potential cases. While the admitting diagnoses can be overturned after admission [e.g., rule-out-MI scenarios] or may just indicate a previous history of an event that required consideration during the current admission but was not a current event, we still include these as potential cases. Cardiac enzyme laboratory values and ECG results can be extracted.

The definition of definite myocardial infarctions is based on third universal definition criteria. In our experience cardiac biomarker data are generally available. In a small review of 30 charts of potential MI, we found this to be the case. Based on this manual review of data extracted from 30 charts we are confident that these cases can be identified and adjudicated accurately and only a small fraction will require manual adjudication.

Stroke: For stroke using codes 430, 431, 432.x, 434.01, 434.11, 434.91, we were able to identify potential cases. Our review of 30 charts also provided confidence that the data elements for adjudication are available in the electronic records of potential cases. Based on the review of 30

charts, it appears that there will be a slightly higher number of indeterminate cases that will need manual adjudication than for MI but the proportion is still modest.

Unstable Angina with revascularization: The potential cases are easy to identify using a diagnostic code for coronary revascularization (e.g., ICD9 36.x or CPT 33510-45 or 92920-44 or 92997-8). Additional data on the reason for hospitalization such as a code for unstable angina (e.g., ICD9 411.1-.89, and we will explore 413 and 786.5) on the same admission as or up to 15 days before an inpatient procedure code can also be easily obtained. Additional data such as use of an appropriate platelet inhibitor can also be extracted. As there was greater heterogeneity among potential cases, further work will be required to create an acceptable algorithm. As expected we found a number of patients in this preliminary review that underwent coronary revascularization, but did not meet the unstable angina criteria. Further work will be needed to refine how to improve our identification of these cases and adjudication of these potential cases to reduce the number that will be needed to be manually adjudicated.

Acute hospitalization for congestive heart failure: This outcome is more complex than the previous three because it relies more heavily on unstructured data. We will construct an algorithm using such features as: a hospitalization ICD code for heart failure (398.91,402.01, 402.11, 402.91, 404.01, 404.03, 404.11, 404.13, 404.91, 404.93, 425.4x-425.9x, 428.x, 429.3,

514) whether this code is labeled as “length of stay” diagnosis or not. Because of the broad range of ICD-9 codes (15 unique codes) listed for acute congestive heart failure we uncovered considerable heterogeneity in the cluster of potential cases with these codes. We did find that certain ICD-9 codes resulted in a low yield for the primary outcome category. For example, none of the patients reviewed with an ICD-9 code 425.4 “Other primary cardiomyopathies”, met the definition criteria for acute congestive heart failure. Additional features such as absence of a code for implantable cardioverter defibrillator placement (generally associated with a chronic rather than acute case of CHF in our review), and evidence of intensification of treatment (as defined above by the FDA expert panel and which can be extracted successfully from the medical record) will be used to construct the screening tools and the algorithms that will minimize the need for manual adjudication.

G. Adjudication of events

In cases where the outcome diagnosis is not clear based on the electronic adjudication, we will resort to manual adjudication to determine the validity of the diagnosis. Based on our 150 chart review, it is expected that fewer than 300 to 400 cases will require this form of adjudication. The adjudication will consist of a chart review by a physician of medical records pertaining to the hospital admission for VA admissions and a chart review of VA inpatient and outpatient records as well as CMS data following the discharge date for non-VA hospitalizations. In some cases, we may query the PCP as to whether a possible event occurred.

We will also manually adjudicate 10% of outcomes confirmed or refuted by our algorithms to serve as a quality check on the algorithms. We will develop near-final algorithms during the first year of the study, but will continue to refine the algorithms and retrospectively apply these refinements to all data.

H. Ascertainment of potential cases, adverse events and other case data

Investigators at the MAVERIC CSPCC unaware of treatment group will process potential cases and adverse events by collecting relevant codes, laboratory values, and prescription data from CPRS, the VA electronic medical record system (e.g., acute gout episode = ICD9 274.01). Blood pressures will be collected from routine clinic visits, and not from emergency room visits or inpatient stays, and only the reading with the lowest SBP of the day will be retained (99).

Section XIII details the data sources and the data collection (i.e., data extraction) procedures that will be used. When the study drug is discontinued, we may query the PCP as to the reason if not clear from the chart.

XI.Biostatistical Considerations

A. Overview of the Study Design

The proposed study is a prospective randomized open-label blinded-endpoint, multicenter, two arm intervention trial testing the effectiveness of chlorthalidone for prevention of cardiovascular events and non-cancer death among patients currently receiving hydrochlorothiazide. The primary hypothesis is that chlorthalidone is superior to hydrochlorothiazide for the prevention of cardiovascular events and non-cancer death over time.

The primary outcome measure is time from enrollment in the study to the first occurrence of a cardiovascular event or non-cancer death. Cardiovascular (CVD) events are defined as stroke, myocardial infarction, urgent coronary revascularization, and hospitalization for acute congestive heart failure. The results for CVD and non-cancer death event-free survival will be analyzed by means of a two-sided log-rank test.

The study will have one interim analysis and one final analysis.

1. Estimated Incidence of the Primary Endpoint

This study will enroll a total of 13,500 patients, 6,750 of whom will receive chlorthalidone and 6,750 of whom will receive hydrochlorothiazide. We expect to enroll, on average, 90 patients per year for three years to accrue approximately 270 patients per site at 50 VA medical centers. All subjects will be followed through the end of the four and one-half (4.5) year study period yielding an average follow-up time of three (3) years. We posit a four and one-half year rate of 13.5% of the composite outcome in the hydrochlorothiazide group and 11.1% in the chlorthalidone group. We utilized the VA National data from fiscal years 2010 to 2012 to identify a subgroup of subjects who would be potentially eligible for the proposed study. Details of the analysis can be found in **Section XII. Feasibility and Recruitment Plan**.

The cardiovascular event rate, using a composite similar to that proposed here, was 2% per year in ALLHAT (20) and ACCORD (101), and is projected as 2% for the ongoing SPRINT. Unlike those studies, we are limiting enrollment to patients over age 65 years and are not excluding very old or seriously ill patients (unless life expectancy is known to be less than 6 months), and we are including all non-cancer deaths rather than only cardiovascular deaths, all of which would be expected to increase the event rate. For patients 65-79 years old with no serious illness (and creatinine <1.5 mg/dL) in ACCORD, the composite event rate (stroke, myocardial infarction, and cardiovascular death) was 2.8% per year (96). Our event rate should be higher because we include urgent revascularization, acute heart failure, and non-cancer deaths. ANBP2 enrolled patients over 65 years but included all deaths in the composite, and observed an event rate of

>4% per year (19).

We believe that the most relevant event rates for our study come from the Ontario observational study (29) comparing chlorthalidone with hydrochlorothiazide, discussed above. The Ontario study is both recent, published in 2013 and reporting data from 1993 to 2010, large (nearly 30,000 patients), and shares important features with our proposed trial, including: 1) study patients are aged 66 and older and taking diuretics for hypertension, 2) outcome data were collected passively from administrative databases, and 3) the primary outcome was a composite similar to the one we are proposing, with two differences: a) they did not include urgent revascularization, and b) they included all deaths whereas we do not include cancer deaths.

We expect these 2 differences to very nearly cancel each other’s effect. The rate for the Ontario composite outcome was 3.4% per year in the hydrochlorothiazide group. The rate for all deaths was 1.8% per year, or 45% of the total number of individual outcomes (some patients had more than one of the composite outcome elements). Many studies, such as the Physicians Health Study

(95) have found that all deaths in this age group are comprised of roughly 1/3 each of deaths due to cardiovascular disease, cancer and other causes, suggesting that our exclusion of cancer deaths would reduce the composite rate by 15%. In the (TRA2°P)-TIMI 50 trial (86), urgent coronary revascularization was less frequent than myocardial infarction (0.82% in the Ontario study, or 20% of the total number of individual outcomes) and similar in frequency to stroke (0.46% in the Ontario study, or 11% of the total number of individual outcomes), suggesting that our inclusion of this outcome will raise the composite by 10-15%.

These adjustments result in a best estimate for the expected composite rate of about 3.2% per year. For the proposed study, because event rates tend to decrease over time, we conservatively project a 3% per year event rate in the hydrochlorothiazide group.

2. Effects of the Intervention

Of 125,000 VA patients started on drug in routine practice in 2004, 72% of hydrochlorothiazide users and 62% of chlorthalidone users remained on the drug 1 year later (28). Onsite coordinators in standard trials may be able to maintain better drug adherence than occurs in usual practice. ACCORD (101) and SPRINT (84) considered a 20% relative reduction to be an appropriate minimum important difference for power calculations. Because the difference in outcomes in our study could be reduced by patients coming off drug more often than in previously published trials, we reduce the minimum important difference value to **17.5%**. A reduction in CVD events or non-cancer death of this magnitude or greater would be considered clinically significant.

B. The Primary Analysis

We posit a four and one-half event rate of 13.5% in the hydrochlorothiazide group and a 17.5% reduction of CVD events in the chlorthalidone group to inform our primary hypothesis.

Formal statement of the primary hypothesis

Under the null hypothesis:

The 4.5 year event rate will be 13.5% (or 578 primary events)

Under the alternative hypothesis for study participants treated with chlorthalidone:
The 4.5 year event rate will be 11.1% (or 477 primary events)

The reductions attributed to chlorthalidone may be viewed in several ways. The absolute reduction from 13.5% to 11.1% is 2.4%, the relative reduction is (13.5 – 11.1)/13.5 = 17.5%, and the hazard ration (chlorthalidone hazard rate/hydrochlorothiazide hazard rate) of 0.81 is approximately midway between the simple odds ratio, 11.1(100-13.5)/13.5(100-11.1) = 0.80 and the risk ratio 11.1/13.5 = 0.82.

Formally, the null hypothesis is that the two treatment groups do not differ in their time-to-event hazard rates. The alternative hypothesis is that chlorthalidone has a lower or higher hazard rate than hydrochlorothiazide therapy with a hazard ratio for chlorthalidone compared to hydrochlorothiazide less than 0.82 or greater than 1.22. We will test this hypothesis with a two- sided log-rank test.

C. Sample Size and Statistical Power Considerations for the Primary Hypothesis

The formal hypothesis test is two-sided allowing for chlorthalidone to be either more or less effective than hydrochlorothiazide. A significant difference showing that the intervention chlorthalidone compared to the control hydrochlorothiazide decreases (or increases) the hazard of a major cardiovascular event will be regarded a positive result. The results for the primary outcome measure will be analyzed by means of the two-sided log-rank test to detect either a hazard ratio that exceeds 1.22 or is less than 0.82. The test will have a two-sided 5.9% type I error. The test has 90% power to detect a hazard ratio of 1.22 or larger or 0.82 or less with a total of 13,500 study participants, 6,750 per arm. The remaining Type I error of 0.1% is used for the interim analysis.

If the annual event rate is 2.5% (rather than the posited 3%), then the study has 84% power; if 2%, it has 75% power.

D. Primary Data Analysis

The primary outcome hypothesis will be a time-to-event analysis with the use of a two-sided log-rank test based on intention-to-treat principles. The model will not include any covariates.

Analytic reports will include hazard rates, their ratio, and the 95% confidence interval about the ratio.

This will be followed by further refined covariate-adjusted exploratory analyses, using Cox proportional hazards regression modeling, controlling for baseline factors. Covariates will include demographic factors (e.g., age, sex, smoking status, education) and clinical factors (e.g.,

blood pressure, medications, comorbidities, history of disease, and BMI). We will test the proportional hazards assumption by including a time-treatment interaction term in the model.

E. Interim Analysis

We will perform one interim analysis when the 500th event occurs, approximately 3.5 years after initiation of enrollment. Assuming a uniform rate of enrollment over time, the first patient entered will potentially have 3.5 years of follow-up and the last patient entered will potentially have 0.5 years of follow-up. Thus, we will have an average follow-up time of approximately 2 years on 13,500 subjects.

Using the O-Brien Fleming procedure, this interim analysis will have a type I error of 0.1%, which negligibly decreases the overall type I error and has virtually no effect on the power to show that chlorthalidone is different from hydrochlorothiazide. The sample size of 13,500 accounts for the interim analysis with a corresponding inflation factor, for increase in sample size of 1.001, resulting in14 more subjects per arm (102). We will confer with the Data Monitoring Committee (DMC) members and the program leadership for potential stopping guidelines based on findings from the interim analysis.

F. Secondary Data Analysis

1. Treatment Effect in Subgroups
   The Cox regression modeling will explore the possibilities of treatment variation across pre-specified subgroups based on status at time of enrollment including:

   a) gender,
   b) age (dichotomized at median),
   c) baseline SBP (dichotomized at median),
   d) history of MI or stroke,
   e) black race vs. not,
   f) diabetes vs. not,
   g) eGFR < 60, and
   h) good compliance (medication possession ratio ≥ 80%) with hydrochlorothiazide over the year before randomization.

2. Individual Components of Primary Outcome
   In addition to the subgroup analyses listed above, each component of the composite primary outcome measure (stroke, myocardial infarction, urgent coronary revascularization, hospitalization for acute congestive heart failure, and non-cancer death) will be separately analyzed using log-rank and Cox proportional hazards models to compare chlorthalidone and hydrochlorothiazide. The Cox analyses will include the covariates considered in the primary data analysis.

3. Additional Outcomes of Interest (Analysis of Secondary and Tertiary Objectives)

In addition we will run time-to-event analyses comparing the treatment effect on:

a) all-cause mortality,
b) the composite outcome substituting all deaths for non-cancer deaths,
c) vascular deaths defined as all deaths caused by diabetes, vascular diseases, external causes, and unknown causes,
d) the composite outcome substituting vascular deaths for non-cancer deaths,
e) any revascularization of an artery,
f) hospitalization for primary diagnosis of hypokalemia, hyponatremia, or renal failure,
g) other hypokalemia (< 3.5 meq/L), hyponatremia (< 130 meq/L), or renal failure,
h) new diabetes, requiring medications, defined as first use of medication for diabetes,
i) acute gout episode,
j) erectile dysfunction (ED), defined as first prescription for PDE5 inhibitor or referral for ED, and
k) new allergic reaction to thiazide-like diuretic, defined as new flag warning in CPRS

4. Primary Care Provider Metrics of Interest.
    a) percent of patients approved
    b) percent of approved patients for whom order signed
    c) reasons order not signed
    d) rate of discontinuation of both drugs
    e) reasons for discontinuation of both drugs

5. Medication Compliance

We will compare treatments with respect to overall compliance (adherence) to the randomly assigned medication, indirectly measured by the medication possession ratio (MPR) and average daily dose (ADD). The comparison will be made using a GEE analysis to account for varying periods of follow-up. The potential period ends if the subject dies, has an outcome event, or changes medication. Subjects categorized as medication compliant will make up the per-protocol subgroup of the study cohort.

6. Non-acute Outcomes

The repeated measures of systolic blood pressure during the disease-free intervals (free of component events) will be compared by treatment. We will use a mixed effects repeated measures model allowing for irregular time intervals, the spatial power law extension of AR(1) covariance option where r(t1,t2) = $r(t_1, t_2) = \exp(|t_1 - t_2|)$. In each model we will assume linear growth over time. A random effect will be included for both intercept and linear time. The hypotheses will test if SBP increases/decreases over time for subjects receiving chlorthalidone, if SBP increases/decreases over time for subjects

receiving hydrochlorothiazide, and if both have non-zero slopes, a test of whether they differ or are equal.

We will conduct a time-to-event analysis comparing treatments for the outcome of time to first discontinuation of assigned diuretic (defined as no prescription for ≥ 3 months at last observation during study period) and time to first protocol deviation from assigned diuretic.

G. Exploratory Objectives

The primary intent to treat analyses of randomly assigned treatments takes no account of post-baseline changes such as protocol deviations and treatment switches from chlorthalidone to hydrochlorothiazide (and vice versa). Thus, exploratory analyses will model such changes to assess robustness of conclusions.

The per-protocol analysis will determine if chlorthalidone and hydrochlorothiazide differ among the subset of protocol compliant subjects. We will run other analyses that include all subjects and attempt to model the time-dependent effects of protocol deviations such as medication changes and levels of compliance. Frailty analyses will assess center effects. We will explore censoring patterns such as models that assume not missing-at-random censoring (103).

A change in medication may alter the subsequent risk of a cardiovascular event. First, we will add time-dependent covariates to a Cox model, such as binary indicators of a switch from chlorthalidone to hydrochlorothiazide, switch from hydrochlorothiazide to chlorthalidone, and the start of new medication that interacts with chlorthalidone or hydrochlorothiazide. To directly estimate subsequent risk, we will use a multistage model (MSM) to assess the hazard rate of a major cardiovascular event after a switch (104). MSM models extend the time-to-first- event models to second, third, and more events. This extension of the Cox model allows direct estimation of the hazard rate associated with any transition adjusted for previous history and baseline characteristics. Within MSM we will carry out a competing risks analysis to assess the effect of cancer deaths.

H. Randomization

To control for potential imbalance in randomization, both stratification and blocking will be employed. The randomization scheme will be stratified by participating site to account for possible regional differences in clinical practice. Participants will be randomized to either hydrochlorothiazide or chlorthalidone within blocks of size 6.

Enrollment and randomization of subjects will occur in accordance with **Section IX. Study Procedures** and **Appendix C. Recruitment Flowchart**.

.

XII.Feasibility and Recruitment Plan

A. Feasibility

In 2012, 69 VA medical centers prescribed single-agent hydrochlorothiazide 25 mg or 50 mg to at least 2000 patients over age 65 (**Appendix E.1**). In VISN1, 80% of these had SBP ≥120 mm Hg at last measurement with no SBP measurement below 120 mm Hg in the previous 90 days (**Appendix E.2**). Applying these regional SBP data to the national medical center data, in 2012 there would have been 76 VA medical centers with ≥1500 eligible patients and 104 centers with

≥1000 eligible patients.

Based on experience with the CONFIRM study (CSP #577) and the Million Veteran Program and other sources, we estimate that: 15% of PCPs will opt out; PCPs who don’t opt out will exclude 5% of their patients; 5% of patients mailed the initial letter will opt out; we will reach 65% of patients we attempt to call; 60% of those we reach by phone will agree to be randomized; 2% of those who agree by phone will opt out or be removed by their PCP before randomization. Combining these rates: (.85)(.95)(.95)(.65)(.6)(.98) = .29, suggesting we should expect to enroll about 30% of eligible patients identified.

Based on these considerations, randomization of 13,500 patients (270 per site at 50 sites) should be feasible. We anticipate that this process will take 3 years, a duration that could be adjusted by the level of staffing of the central call center. If recruitment falls short of our expectations, our primary strategy will be to add additional sites as needed.

B. Recruitment Plans

As previously described the primary recruitment plan will be to enroll patients who have been identified through the corporate data warehouse as being eligible and cleared by their PCP through the direct mailing of an opt-out letter followed by telephone based consent. Enrollment will be initiated in 3 VISNs at the start of the study. This will allow the DCP team to learn about the feasibility of recruitment, to identify issues with provider participation and CPRS use, and to refine the primary recruitment plans based on the experience from the vanguard sites. Patients will also be allowed to self-refer to the study call center as well. In this regard, the DCP will allow all patients fitting the eligibility criteria to be enrolled regardless of how they were identified.

In addition, we will initiate 20% more sites (i.e., 10 additional sites) than what is needed for successful enrollment into the study. As sites fail at recruitment or as the potential patient pool decreases at the vanguard sites, enrollment into the DCP will be started at the 10 additional sites. Should this occur, the financial support for the failing sites will be transferred to the new sites.

XIII.Data Collection and Data Sources

Data for this study will be obtained from the medical and administrative data that are collected and maintained by the United States Department of Veterans Affairs (VA) Corporate Data Warehouse (CDW). This database covers the entire veteran population that utilizes the VA and contains individual information on demographic factors, medical history, key laboratory values, procedure codes, and diagnoses (inpatient and outpatient) coded with the ICD-9-CM classification system. Healthcare encounters outside of the VA system will be captured using Medicare data that will be obtained from VIREC for this project. Data from the various VA databases will be linked together using a unique veteran identification number that is assigned to each veteran at entry into the system.

Deaths will be ascertained from the VA Vital Status File. This file allows for complete ascertainment of death as it pulls data from multiple sources, including: the Beneficiary Identification and Records Locator Subsystem database; the Death Master File from the Social Security Administration; and the National Patient Care Database. Ascertainment of death with this method has demonstrated 98% sensitivity and 98% agreement with the National Death Index.

We will monitor for discontinuation or expiration of the assigned diuretic, detection of which will prompt queries to the PCP asking the reason. No effort will be made by the study to influence blood pressure goals or the prescribing of other drugs.

XIV.Data Management and Data Security Plans

The MAVERIC CSPCC will create and maintain an electronic study database to manage the trial data. All study data will be collected electronically from CPRS by the Coordinating Center throughout the duration of the study. There will be no paper-based study documents.

Study data is housed on secure VA servers, encrypted and protected in accordance with VA policies compliant with FDA requirements, Federal Information Security Management Act and the HIPAA Privacy and Security rules. MAVERIC CSPCC personnel manage the data access request process for the electronic systems to ensure that data access is appropriate for each individual and the level of the individual access. VA's Office of Information & Technology (OI&T) is responsible for managing other VA system access and ensuring the security and integrity of VA information systems, including the databases and servers housing study data. In accordance with VA Handbooks and Directives, OI&T is responsible for ensuring that appropriate firewalls and data security is implemented and maintained, that data backups are performed and that data may be restored in the event of a system malfunction.

Data security incidents will be classified into two main categories for reporting: local incidents (i.e., those occurring at MAVERIC CSPCC), and field-based incidents (i.e., those occurring at the clinical sites managed by the center). Incidents will be reported according to 1058.01. All MAVERIC CSPCC staff will be expected to report data security incidents to the responsible authority as they become aware of the breach. Whenever possible, the reporting of data security incidents will be handled by the MAVERIC CSPCC Associate Center Director for Quality Assurance (ACDQA). This will be done to facilitate communication between the center and the oversight bodies. In the event that an incident must be reported by a staff member other than the ACDQA, all communication after the initial report will be handled by the MAVERIC CSPCC Center Director or the MAVERIC CSPCC ACDQA. [Note: all new and current MAVERIC CSPCC personnel will be trained on reporting data security incidents on a yearly basis.]

All local data security incidents will be reported in accordance with VA policy within one hour of discovering the incident to:

1. The Boston Information Security Officer (ISO)
2. The Boston Privacy Officer (PO)
3. The Boston ACOS for Research
4. The MAVERIC CSPCC Quality Assurance department

The MAVERIC CSPCC will ensure that all field-based data security incidents are reported in accordance with VA policy within one hour of discovering the incident to:

1. The District (local) Information Security Officer (ISO)
2. The District (local) Privacy Officer (PO)
3. The District (local) ACOS for Research

4. The MAVERIC CSPCC Quality Assurance department

In addition, field-based data security incidents will be treated as unanticipated problems by the MAVERIC CSPCC and reported to the VA Central IRB according to the procedure detailed below for unanticipated problems (Section XVII.C.3).

Study data will be coded and stored using a unique study identifier for each participant. Identifiable information will be collected for patient tracking and safety purposes, and kept in an encrypted, password protected file to which a small number of people will have access. Access to the cross-walk file linking the participant's identifiers and their study data will be restricted to the approved personnel at the CSP coordinating center. This file will be destroyed according to CSP policy well after the close of the study.

Access to the study data is restricted to individuals with CSP approval. Individuals must be properly credentialed research staff and must be compliant with VA security trainings (e.g. HIPAA, Rules of Behavior, and Good Clinical Practices). Once formal training is completed, user accounts utilizing a URL specific to the study to access and use the system and enter patient data will be activated. Accounts will be password protected and unique to the each user. The account permissions will correspond with the users' functional study group (i.e., those for a study chair would differ from those of the coordinating center). Furthermore, the permissions of the electronic systems are heavily restricted. The site Liaisons will not have access to study data.

Research data will be stored on VA secure servers with restricted permissions for copying and exporting data. Only properly approved Coordinating Center personnel will have the ability to copy and export data. These individuals have received training on the local standard operating procedure (SOP) governing their permissions. Access to protected health information (PHI) will be restricted to individuals approved by CSP to have access to the data.

At the MAVERIC CSPCC the following staff will have access to all forms of PHI:

1. Center Director
2. Study Director
3. Project Management
4. Nurse Coordinator
5. Data Management
6. Biostatisticians
7. Quality Assurance Officer

8. SAS/Database Programmer
9. Research Assistant

10. Clinical Applications Coordinator

11. Informatics Team

Periodic access control assessments will be made by Coordinating Center Quality Assurance personnel to verify that access is controlled and appropriate for personnel. In addition, the CSPCC QA group will provide continuing education on good clinical practices compliance and will evaluate clinical site operations for violations of VA policies including VA data security policies and GCP.

At the end of the study, the data for DCP will remain property of the Cooperative Studies Program and be stored and shared according to CSP guidelines and procedures. Retention and destruction of data will be conducted according to CSP operating procedures and federal and local VA regulations. This will include electronic data stored at the MAVERIC CSPCC, and at the VA facility housing our servers. Identifiable data will be kept according to CSP policy as outlined in the "CSP Guidelines for the Planning and Conduct of Cooperative Studies".

XV. Human Subjects

A. Waiver of HIPAA authorization

A waiver of HIPAA authorization to use VA data to determine eligibility will be requested because the research could not practicably be conducted without access to and use of this information. In order to conduct the study, it is necessary to first be able to identify eligible patients so that the recruitment letter can be mailed to them.

We will also request IRB approval for a waiver of HIPAA authorization to collect data prospectively. Unlike traditional randomized clinical trials, a feature of the POC methodology is that data collection is performed by passive data capture. All data elements will be collected electronically from CPRS/VISTA using both the Veteran’s Information and Computing Infrastructure (VINCI), a collaborative effort between the VA Office of Information Technology and the VA Office of Research and Development, and the Office of Information Technology’s Corporate Data Warehouse (CDW). Healthcare encounters outside of the VA will be captured using Medicare data. Data from the various VA databases will be linked together and maintained using a unique veteran identification number (not SSN). Access to the cross-walk file linking the participant’s identifiers and their study data will be restricted to approved personnel at the CSP coordinating center.

Because data abstraction is done electronically and not by staff perusing the electronic medical record we believe that a waiver of HIPAA authorization is justified and involves no more than minimal risk to the privacy of individuals, particularly since the protected health information of the patients will remain within the VA. Moreover, it would be impractical to obtain a signed authorization from patients in this study for use of their health information. The PCP’s cannot obtain a written HIPAA authorization for research purposes from patients who are subjects in this study because they will not see the patient until weeks or months after the patient has been on the randomized treatment. The PCPs are not study team members. We plan to identify and disclose to participants in our information statement the health information to be collected and the specific databases from which it will be obtained.

In both instances, the patient eligibility screening and the prospective collection of patient data, the use of the requested information involves no more than minimal risk to the privacy of individuals based on the security measures used by the MAVERIC CSPCC to protect the identifiers from improper use and disclosure, and to destroy the identifiers at the earliest opportunity consistent with conduct of the research. The requested information will not be reused or disclosed to any other person or entity, except as required by law, for authorized oversight of the research study, or for other research for which the use or disclosure of the requested information would be permitted by the Privacy Rule.

B. Basis for waiver of Documentation of Informed Consent

Though we are not planning to obtain written informed consent, multiple measures are in place to ensure that all subjects are given all of the information they need in a manner that is understandable before they consent and throughout the study. Primary care providers will receive information through site liaisons as well as an introductory letter that is both mailed and emailed to their VA addresses prior to receiving the initial testpatient order in CPRS. The initial testpatient order will be sent together with a progress note containing the Provider Information Sheet and by signing the testpatient order, the providers are consenting to participate. Provider participation is completely voluntary and minimal risk, facts that will be reiterated throughout this process.

Patients are sent a transcript of the consent and an explanatory letter with study contact information before the first phone call, full consent is obtained on the call, and they are informed of the randomized group after the call.

In this study, the relevant risks involved are those of changing therapy of half of the patients from hydrochlorothiazide to the equivalent dose of chlorthalidone, a very similar drug with the same indications and metabolic effects, plus the theoretical risk to confidentiality from compiling individual data.

Regarding the former, both drugs have been in use for more than 50 years, have long been available as generics, and are included in the VA national formulary in the same drug class: USP code CV 701, thiazide-type diuretics. The VA frequently selects one member of such a class to make available “on formulary” based on cost or other considerations, and VA Pharmacy and Therapeutics Committees will direct VA pharmacies to substitute drugs within a class when one drug becomes unavailable, so it is not uncommon that VA physicians and patients have limited choice within a class.

While thiazide-type diuretics affect some blood chemical levels as discussed above, these effects have not demonstrated adverse clinical impact that would affect their being the preferred treatment for hypertension. Furthermore, our plan to substitute an equivalent dose of chlorthalidone for the current dose of hydrochlorothiazide, i.e. half the number of milligrams, would be expected to have an effect on blood pressure and on blood chemical levels, small enough to be virtually indistinguishable from the variation in these parameters that would ensue with remaining on the prescribed hydrochlorothiazide (see Background and Rationale, parts F and I). We therefore propose that the study intervention qualifies as minimal risk. Because the two drugs are used interchangeably, and the choice between them is not influenced by any patient factors, but only by physician preference, randomization does not reduce individualization of patient care. Care remains personalized after randomization because the PCP manages the diuretic as usual. The patient’s needs are thus not subordinated to the needs of the

trial. Furthermore, because hypertension is a chronic condition, randomized patients will themselves be among those expected to benefit from the information gained from this study.

In their article "Randomized, controlled trials as minimal risk: an ethical analysis", Morris and Nelson (106) conclude that "A randomized, controlled trial poses no more than minimal risk only when all of the following five criteria are met: 1) genuine clinical equipoise exists; 2) all of the treatment options included in the research study fall within the current standard of care; 3) there is no currently available treatment with a more favorable risk-benefit profile than the treatments included in the research study; 4) the nontherapeutic components of the research are safely under the minimal risk threshold; and 5) the research protocol provides sufficient latitude for treating physicians to individualize care when appropriate." We have designed this study to meet all of these criteria.

We believe that the waiver of Documentation of Informed Consent that we are requesting is necessary to the successful completion of our study because obtaining the required sample size within the VA is only feasible if recruitment is maximally efficient, as described in the Feasibility section (XII.A). Published evidence demonstrates that requiring that written consent be returned by mail is likely to result in the loss of the great majority of patients who intend to consent (107-111). If requirement of returned written consent caused loss of most willing patients from our study, it is unlikely that it could be completed within the VA system.

Based on these considerations, we will request IRB approval of a Waiver of Documentation of Informed Consent. Under the terms we are requesting for the Waiver, patients would be recruited by mail, and the elements of consent obtained over the phone, a written summary of the consent will be sent to the patient, but a signed returned document would not be required.

Enrolled patients will be given study contact information for questions or withdrawal.

C. Engagement in Research

For this study, we consider the site Liaison and the PCP's to be not engaged in research. The site Liaison (sometimes called a "Champion" in other studies) serves to provide information to local site personnel about the study and relays information and questions back to the coordinating center, but in our view takes no action that qualifies as research. The PCPs facilitate implementation of the intervention (after patients provide consent and are randomized by study personnel) by signing an order sent to them by study personnel. The PCPs are themselves research subjects in whom we are studying the implementation of the protocol.

XVI. Quality Control Procedures

Data that is extracted for the DCP will be cleaned and managed according to a rigorous data management plan that will be written in conjunction with the statistical analysis operations plan and the study operations manual (for chairs office and CSPCC personnel). However, the data will also be subject to quality control procedures. As a first line effort to ensure the validity of the data, 100% of the data elements collected from the first 10 individuals from each medical center will be subject to source verification and validation through chart review that will be done by an experienced clinician and clinical applications coordinator. In addition, chart review done through the creation of the algorithms to identify outcomes for the DCP will also be used to perform QC procedures on the data.

In addition, the quality management system (QMS) in place at the CSPCC will ensure further quality control for the DCP. The Quality Assurance Department of the CSPCC will subject the data to risk based audits that will verify and validate data elements according to internal SOPs for conducting risk based monitoring and auditing. In brief, a sample of the data will be verified at routine intervals. If errors are identified they will be referred to the data management teams at the MAVERIC CSPCC or at VINCI for resolution. If the error level rises to a predefined threshold, then the entire record or data element type will be subjected to verification and validation. Further, the CSPCC will conduct internal audits to ensure the quality of the clinical trial processes and procedures. If deviations or non-conformances are identified they will be remedied through the internal corrective action/preventive action system of the QMS.

XVII.Study Monitoring Plan

A. Introduction

The safety issues related to hydrochlorothiazide and chlorthalidone are well established in the medical literature and both diuretics are accepted first-line treatments for hypertension. Based on this information the study poses minimal risks to participants beyond the expected adverse events (AE) associated with the administration of either drug as part of “usual” care.

Monitoring side effects and adverse events in the traditional manner of usual clinical trials is not feasible for Point of Care studies since there are no site personnel and all data is captured passively through the EMR. In addition, real-time monitoring will neither provide new information regarding the safety of these two treatments nor assure adequate (or timely) safety of human subjects beyond that already done by the medical staff as part of routine medical care. Accordingly, we propose an alternative safety reporting plan for the DCP study that ensures protection of the participants and that complies with VHA Policy (i.e., VHA Handbooks 1200.05 and 1058.01). In brief, health providers will identify, monitor, and treat (as necessary) adverse events that occur during the course of the study. The CSPCC will identify the events through the EMR and report them to the monitoring committees for the trial. The sections that follow describe in more detail the proposed safety monitoring and reporting plan for this trial.

B. Safety Monitoring Plan

The participant’s physicians, nurses, and other health providers will continue their usual monitoring of the subject throughout the course of his/her treatment. If any treatments are indicated, they will be provided by health providers as a part of the participant’s routine medical care. The CSPCC staff will collect safety data from the medical record from the time of consent through the end of the study period. The safety data will then be aggregated and classified according to ICD-9 codes.

Aggregated safety data will be reported to the Data Monitoring Committee, the study Executive Committee, and the VA Central IRB using the processes described in Section C. Please refer to Section XVIII Study Organization and Administration for a more detailed description of the oversight committees for the trial.

In addition to data culled from the EMR, the trial will allow for spontaneous reporting of events by study participants. The informed consent script and information sheet will include the contact information for the study coordinating center personnel should the participant wish to communicate safety concerns with the study team.

C. Safety Reporting

1. Adverse Events

Adverse events will be collected using the 21 CFR 312.32, International Conference on Harmonisation (ICH) for Clinical Safety Data Management (ICH-E2A), and CSP Global SOP 3.6 definitions. Adverse events (AEs) are defined by the 21 CFR 312.32 as "any untoward medical occurrence associated with the use of a drug in humans, whether or not considered drug related."

According to ICH-E2A,”an AE, therefore, can be any unfavorable or unintended sign (including an abnormal laboratory finding), symptom, or disease temporally associated with the study interventions.”

Expected adverse events of interest related to diuretics will be culled from the EMR as part of the outcome ascertainment activities of this protocol. The expected AEs of interest will be:

1. Hospitalization for primary diagnosis of hypokalemia, hyponatremia, or renal failure
2. Renal failure (dialysis, vascular access for dialysis, renal transplant)
3. Other recorded hypokalemia (< 3.1 mEq/L), or hyponatremia (< 130 mEq/L)
4. New diabetes, defined as first use of an outpatient medication for diabetes
5. Acute gout episodes
6. New allergic reaction to thiazide-type diuretic, defined as new entry in Allergies/Adverse reaction (ART) database

These safety events are monitored and treated as part of routine medical care. These events will be identified by the CSPCC through the electronic medical record (EMR). Informatics staff at the MAVERIC CSPCC will extract medical record data on all subjects monthly and identify adverse events through ICD-9 codes, laboratory values, and medication files. Expected adverse events will be reported to the DMC as secondary and tertiary outcomes in semi-annual reports. Data will be in the form of aggregated data tables detailing the frequencies of these events by blinded treatment group. These events will be reported to the Central IRB in blinded aggregate form at the time of continuing review.

Adverse events which develop into Serious Adverse Events, as defined below, will be reported as such.

2. <u>Expected Serious Adverse Events</u>

Serious adverse events (SAEs) are a subset of adverse events defined in 21 CFR 312.32(a) and VA Handbook 1058.01 paragraph 4(w), as follows:

<u>Definition of SAE from CFR 312.32 (a):</u> Serious adverse event. An adverse event or suspected adverse reaction is considered "serious" if, in the view of either the investigator or sponsor, it results in any of the following outcomes: Death, a life-threatening adverse event, inpatient hospitalization or prolongation of existing hospitalization, a persistent or significant incapacity or substantial disruption of the ability to conduct normal life functions, or a congenital

anomaly/birth defect. Important medical events that may not result in death, be life-threatening, or require hospitalization may be considered serious when, based upon appropriate medical judgment, they may jeopardize the patient or subject and may require medical or surgical intervention to prevent one of the outcomes listed in this definition.

Definition of SAE according to VA Handbook 1058.01: An SAE is an AE in human research that results in death, a life-threatening experience, inpatient hospitalization, prolongation of hospitalization, persistent or significant disability or incapacity, congenital anomaly, or birth defect. An AE is also considered serious when medical, surgical, behavioral, social, or other intervention is needed to prevent such an outcome.

The intervention in this study is the switch from hydrochlorothiazide to an equivalent dose of chlorthalidone, two widely-used diuretics with well-known risk profiles. There are no safety events that are unanticipated in regard to these two drugs. Thus, serious adverse events defined above that are feasibly identified through the medical record will be reported in aggregate to the DMC at 6 month intervals and to the Central IRB at continuing review.

Informatics staff at the MAVERIC CSPCC will extract medical record data on all subjects monthly and will identify adverse events through ICD-9 codes, laboratory values, medication files and the VA vital status files (i.e., BIRLS and the Master Death File). Expected serious adverse events will be reported to the IRB at the time of continuing review in aggregated data tables detailing the frequencies of these events. Study reports will also be circulated to appropriate members, including the study chairmen and DMC.

This study will not use MedDRA coding of AE and SAE data. The study team will define events using the data sources described above and categorize events by system organ class and assessment type for reporting.

3. Unanticipated Problems Involving Risks to Subjects and Others

Any unanticipated problems involving risks to subjects or others (UPRs), but not qualifying as a serious adverse event by definition (such as errant distribution of study medication), will also be reported. Unanticipated problems related to the study design will be reported to the IRB in an expedited fashion (within 5 business days of identifying the problem). Possible events include failure to distribute study drug, patient continuing previous prescription while taking study drug, or patient randomized without provider knowledge. Informatics staff at the MAVERIC CSPCC will extract medical record data on all subjects monthly allowing for the identification of UPRs at fixed intervals. Study reports will be circulated to appropriate members, including the study chairmen.

UPRs that result in an SAE, pursuant to the definition above, will be reported as an SAE to the VA Central IRB within 5 business days by the MAVERIC Boston CSP Coordinating Center after becoming aware of the event.

XVIII.Study Organization and Administration

A. Administration

The administrative structure of this study is similar to others in CSP and includes:

The Cooperative Studies Program (VA Central Office) establishes overall policies and procedures that are applied to all VA cooperative studies through the Study Chair’s office and the CSPCC.

The CSPCC and the Study Chair’s office jointly will perform the day-to-day scientific and administrative coordination of the study. These include developing and revising the study protocol; abstracting data from the national databases (CSPCC Only); ensuring the appropriate support for the participating centers; scheduling meetings and conference calls; answering questions about the protocol; conducting site visits; publishing newsletters. The CSPCC will also prepare interim and final progress reports; and archive study data at the end of the study. Study progress reports will be produced every 6 months. Patient accrual, patient safety, and data quality will be monitored closely by the CSPCC, the study executive committee, and the DMC to ensure that the study is progressing satisfactorily. Further delineation of responsibilities will be documented in communications with the Study Chair’s office.

The CSPCC will be responsible for monitoring and reporting the safety of trial participants through the review, assessment, and communication of adverse events and serious adverse events detected within the national data systems. The CSPCC will document trends and analyze safety data to prepare reports for various committees including the DMC, VA Central IRB (CIRB), Executive Committee(s), and Study Group meetings.

The Canandaigua VA Medical Center will serve as a centralized call center for this study. The CSPCC will work closely with Canandaigua to track eligible participants as they move through the recruitment and enrollment workflow. The primary responsibilities for the Canandaigua call center include contacting patients by telephone to obtain verbal informed consent and receiving incoming patient phone calls. .

The CSP Clinical Research Pharmacy Coordinating Center (CSPCRPCC) will provide advice and consultation about drug-related matters, review safety information, and serve as a regulatory affairs expert and liaison to the FDA for regulatory matters.

The Clinical Sciences Scientific Evaluation Committee (CSSEC) reviews the scientific merit of all new cooperative study proposals and all ongoing cooperative studies. The committee is composed of both VA and non-VA clinical research scientists, most of whom have had experience in managing their own cooperative studies.

The Study Group will be composed of the SLs from each participating center, the Study Chair, Study Director, and CSP staff (biostatistician, project manager, and others). The Study Chair will

head the group, which will meet once a year to discuss the progress of the study, any problems that the study team has encountered, and any suggestions for improving the study.

B. Monitoring

The following groups monitor the various aspects of the study. These committees will meet according to current Cooperative Studies Program guidelines.

The Executive Committee is responsible for the operations of the study, including protocol amendments, and overall management of the study. It will be headed by the Study Chair and Study Director and consist of the study biostatistician, study project manager, CSP Center Director, selected VA experts, and outside consultants as needed. This committee will meet regularly to review blinded data (not broken down by treatment group), decide upon changes in the study, determine the fate of hospitals whose performance is substandard, initiate any subprotocols, and discuss publication of the study results. This Committee must grant permission before any study data may be used for presentation or publication.

The Technical Committee will advise the Executive Committee on informatics and database related issues pertaining to study operations. This committee will serve as subject matter experts for the study database, web application, ETL procedures and primary data source, the Corporate Data Warehouse. It will consist of a VINCI representative, MAVERIC Director of Informatics, and other informaticians/SMEs as needed.

The Data Monitoring Committee (DMC) will review the progress of the study and will monitor patient intake, outcomes, adverse events, and other issues related to patient safety. Interim, independent, and unbiased reviews of the study's ongoing progress will be provided. The DMC will consist of experts in the study's subject matter field(s), clinical trials, biostatistics, and ethics. These individuals will not be participants in the trial and will not have participated in the planning of the protocol. The DMC will consider safety or other circumstances as grounds for early termination, including either compelling internal or external evidence of treatment differences or the unfeasibility of addressing the study hypothesis (e.g., poor patient intake, poor adherence to the protocol).

At each of its meetings during the study period, the DMC will review the randomization rates and assess the difference between the actual and the projected rates, as well as the impact of these assessments on overall trial size. If the study enrollment is inadequate, the reasons for exclusion may be scrutinized and actions may be suggested. An assessment of whether the trial should be continued will be made followed by recommendations, as appropriate. All serious adverse events will be reported regularly to the DMC for review. Unexpected, related serious adverse events will be reported to the DMC as soon as they become known based upon the consensus of the Study Chair, the study biostatistician, the Study Director. The study

biostatistician will provide the appropriate data to the DMC at specified intervals for this purpose. Conditional power estimates may be provided to the DMC to assist them in making

their decisions and recommendations at their request. To help them make their assessment, the Study Chair and study biostatistician will furnish the Data Monitoring Committee with appropriate monitoring data before each meeting. The DMC makes recommendations after each meeting to the Director of the Clinical Science Research and Development (CSRD) Service about whether the study should continue or be stopped.

The VA Central IRB will be the IRB of record for all VA sites. They will monitor the study’s serious adverse events on a continual basis. They will conduct annual reviews of the study. In addition, some study materials (such as subject correspondence and protocol changes) will have to be reviewed by the VA CIRB, and approved prior to implementation.

The CSPCC Human Rights Committee (HRC) is composed primarily of lay people and is responsible for ensuring that patients' rights and safety are upheld prior to study initiation and during the conduct of the study. The committee reviews all new protocols, periodically makes site visits to participating centers to monitor firsthand the progress of the study, and may be asked to review any ethical and human rights issues that arise during the conduct of the study.

XIX. Publications

A. Publication policy

It is the policy of the CSP that outcome data will not be revealed to the participating investigators until the data collection phase of the study is completed. This policy safeguards against possible biases affecting the data collection. All presentations and publications from this study will be done in accordance with current CSP Guidelines, including the Authorship Policy. The most current version of the Guidelines should be referenced when planning any study publication.

The presentation or publication of any or all data collected by participating investigators on patients entered into the VA Cooperative Study is under the direct control of the study's Executive Committee. No individual participating investigator has any inherent right to perform analyses or interpretations or to make public presentations or seek publication of any or all of the data other than under the auspices and approval of the Executive Committee.

The Executive Committee has the authority to establish one or more publication subgroups of investigators and members of the Executive Committee for producing scientific presentations and publications. Authors with VA appointments must list their VA affiliation first. The VA contributions to the research project should be acknowledged in all written and oral presentations of the research results, including scientific articles, news releases, news conferences, public lectures, and media interviews.

All study reports and journal manuscripts must be reviewed and approved by the MAVERIC CSPCC Director prior to submission for publication. After approval for submission is granted by the MAVERIC CSPCC Director, VA Central Office must be notified upon acceptance of any publications. This includes minor publications such as abstracts and poster presentations.

B. Planned Publications

A list of planned publications is below:

I. Editorial: The first large clinically integrated VA trial

II. Design of the DCP

III. Ascertainment of urgent revascularization from administrative data

IV. Ascertainment of acute heart failure episodes from administrative data

V. Main outcomes paper

VI. Blood pressure control and drug compliance in the DCP

VII. Blood chemistries in the DCP

XX. References

1. http://www.cdc.gov/bloodpressure/hypertension_iom.htm
2. http://www.webmd.com/news/20110420/the-10-most-prescribed-drugs
3. http://www.healthquality.va.gov/hypertension/htn04_pdf1.pdf
4. Chobanian AV, Bakris GL, Black HR, et al. The Seventh Report of the Joint National Committee on Prevention, Detection, Evaluation, and Treatment of High Blood Pressure: the JNC 7 report. JAMA 2003;289:2560-72.
5. Psaty BM, Lumley T, Furberg CD, Schellenbaum G, Pahor M, Alderman MH, Weiss NS. Health outcomes associated with various antihypertensive therapies used as first-line agents: a network meta-analysis. JAMA 2003;289:2534-44.
6. Messerli FH, Bangalore S. Half a century of hydrochlorothiazide: facts, fads, fiction, and follies. Am J Med 2011;124:896-9.
7. Ernst ME, Lund BC. Renewed interest in chlorthalidone: evidence from the Veterans Health Administration. J Clin Hypertens (Greenwich). 2010;12:927-34.
8. Kaplan NM. Chlorthalidone versus hydrochlorothiazide: a tale of tortoises and a hare. Hypertension 2011;58:994-5.
9. Mitka M. Experts argue not all diuretics the same. JAMA 2007;298:31.
10. Kaplan NM. The case for low dose diuretic therapy. Am J Hypertens 1991;4:970-1.
11. Materson BJ, Cushman WC, Goldstein G, Reda DJ, Freis ED, Ramirez EA, Talmers FN, White TJ, Nunn S, Chapman RH, et al. Treatment of hypertension in the elderly: I. Blood pressure and clinical changes. Results of a Department of Veterans Affairs Cooperative Study. Hypertension 1990;15:348-60.
12. Cushman WC, Khatri I, Materson BJ, Reda DJ, Freis ED, Goldstein G, Ramirez EA, Talmers FN, White TJ, Nunn S, et al. Treatment of hypertension in the elderly. III. Response of isolated systolic hypertension to various doses of hydrochlorothiazide: results of a Department of Veterans Affairs cooperative study. Department of Veterans Affairs Cooperative Study Group on Antihypertensive Agents. Arch Intern Med 1991;151:1954-60.
13. Materson BJ, Oster JR, Michael UF, Bolton SM, Burton ZC, Stambaugh JE, Morledge J. Dose response to chlorthalidone in patients with mild hypertension. Efficacy of a lower dose. Clin Pharmacol Ther 1978;24:192-8.

14. Morledge JH, Ettinger B, Aranda J, McBarron F, Barra P, Gorwit J, Davidov M. Isolated systolic hypertension in the elderly. A placebo-controlled, dose-response evaluation of chlorthalidone. J Am Geriatr Soc 1986;34:199-206.

15. Neff KM, Nawarskas JJ. Hydrochlorothiazide versus chlorthalidone in the management of hypertension. Cardiol Rev 2010;18:51-6.

16. James PA, Oparil S, Carter BL, et al. 2014 evidence-based guideline for the management of high blood pressure in adults: report from the panel members appointed to the Eighth Joint National Committee (JNC 8). JAMA 2014;311:507-20.

17. Dorsch MP, Gillespie BW, Erickson SR, Bleske BE, Weder AB. Chlorthalidone reduces cardiovascular events compared with hydrochlorothiazide: a retrospective cohort analysis. Hypertension 2011;57:689-94.

18. Ernst ME, Neaton JD, Grimm RH Jr, Collins G, Thomas W, Soliman EZ, Prineas RJ; Multiple Risk Factor Intervention Trial Research Group. Long-term effects of chlorthalidone versus hydrochlorothiazide on electrocardiographic left ventricular hypertrophy in the multiple risk factor intervention trial. Hypertension 2011;58:1001-7.

19. Wing LM, Reid CM, Ryan P, Beilin LJ, Brown MA, Jennings GL, Johnston CI, McNeil JJ, Macdonald GJ, Marley JE, Morgan TO, West MJ; Second Australian National Blood Pressure Study Group. A comparison of outcomes with angiotensin-converting--enzyme inhibitors and diuretics for hypertension in the elderly. N Engl J Med 2003;348:583-92.

20. ALLHAT Collaborative Research Group. Major outcomes in high-risk hypertensive patients randomized to angiotensin-converting enzyme inhibitor or calcium channel blocker vs diuretic: The Antihypertensive and Lipid-Lowering Treatment to Prevent Heart Attack Trial (ALLHAT). JAMA. 2002;288:2981-97.

21. Roush GC, Holford TR, Guddati AK. Chlorthalidone compared with hydrochlorothiazide in reducing cardiovascular events: systematic review and network meta-analyses. Hypertension 2012;59:1110-7.

22. Carter BL, Ernst ME, Cohen JD. Hydrochlorothiazide versus chlorthalidone: evidence supporting their interchangeability. Hypertension 2004;43:4-9.

23. Ernst ME, Carter BL, Goerdt CJ, Steffensmeier JJ, Phillips BB, Zimmerman MB, Bergus GR. Comparative antihypertensive effects of hydrochlorothiazide and chlorthalidone on ambulatory and office blood pressure. Hypertension 2006;47:352-8.

24. Kwon BJ, Jang SW, Choi KY, Kim DB, Cho EJ, Ihm SH, Youn HJ, Kim JH. Comparison of the efficacy between hydrochlorothiazide and chlorthalidone on central aortic pressure

when added on to candesartan in treatment-naïve patients of hypertension. Hypertens Res. 2013;36:79-84.

25. https://vaww.cmopnational.va.gov/cmop/PBM/Clinical Guidance/Archived Criteria, Guidelines and Reviews/Clinical Recommendations (Archive)/Thiazides in Hypertension, Review of Recent Evidence (Archived Dec 2014).doc

26. Ernst ME, Carter BL, Zheng S, Grimm RH Jr. Meta-analysis of dose-response characteristics of hydrochlorothiazide and chlorthalidone: effects on systolic blood pressure and potassium. Am J Hypertens 2010;23:440-6.

27. Peterzan MA, Hardy R, Chaturvedi N, Hughes AD. Meta-analysis of dose-response relationships for hydrochlorothiazide, chlorthalidone, and bendroflumethiazide on blood pressure, serum potassium, and urate. Hypertension 2012;59:1104-9.

28. Lund BC, Ernst ME. The comparative effectiveness of hydrochlorothiazide and chlorthalidone in an observational cohort of veterans. J Clin Hypertens (Greenwich). 2012 Sep;14(9):623-9.

29. Dhalla IA, Gomes T, Yao Z, Nagge J, Persaud N, Hellings C, Mamdani MM, Juurlink DN. Chlorthalidone versus hydrochlorothiazide for the treatment of hypertension in older adults: a population-based cohort study. Ann Intern Med 2013;158:447-55.

30. Bakris GL, Sica D, White WB, Cushman WC, Weber MA, Handley A, Song E, Kupfer S. Antihypertensive efficacy of hydrochlorothiazide vs chlorthalidone combined with azilsartan medoxomil. Am J Med. 2012;125:1229.e1-1229.e10.

31. Cushman WC, Bakris GL, White WB, Weber MA, Sica D, Roberts A, Lloyd E, Kupfer S. Azilsartan medoxomil plus chlorthalidone reduces blood pressure more effectively than olmesartan plus hydrochlorothiazide in stage 2 systolic hypertension. Hypertension. 2012;60:310-8.

32. Ernst ME, Moser M. Use of diuretics in patients with hypertension. N Engl J Med. 2009 Nov 26;361(22):2153-64.

33. Dolan E, Stanton A, Thijs L, Hinedi K, Atkins N, McClory S, Den Hond E, McCormack P, Staessen JA, O'Brien E. Superiority of ambulatory over clinic blood pressure measurement in predicting mortality: the Dublin outcome study. Hypertension. 2005 Jul;46(1):156-61.

34. Fagard RH, Celis H, Thijs L, Staessen JA, Clement DL, De Buyzere ML, De Bacquer DA. Daytime and nighttime blood pressure as predictors of death and cause-specific cardiovascular events in hypertension. Hypertension. 2008 Jan;51(1):55-61.

35. Woodman R, Brown C, Lockette W. Chlorthalidone decreases platelet aggregation and vascular permeability and promotes angiogenesis. Hypertension. 2010 Sep;56(3):463-70.

36. Kurtz TW. Chlorthalidone: don't call it "thiazide-like" anymore. Hypertension 2010;56:335-7.

37. Rodenburg EM, Hoorn EJ, Ruiter R, Lous JJ, Hofman A, Uitterlinden AG, Stricker BH, Visser LE. Thiazide-Associated Hyponatremia: A Population-Based Study. Am J Kidney Dis. 2013 Apr 18. [Epub ahead of print]

38. Alderman MH, Piller LB, Ford CE, et al; Antihypertensive and Lipid-Lowering Treatment to Prevent Heart Attack Trial Collaborative Research Group. Clinical significance of incident hypokalemia and hyperkalemia in treated hypertensive patients in the antihypertensive and lipid-lowering treatment to prevent heart attack trial. Hypertension. 2012;59(5):926-33.

39. Barzilay JI, Davis BR, Cutler JA, Pressel SL, Whelton PK, Basile J, Margolis KL, Ong ST, Sadler LS, Summerson J; ALLHAT Collaborative Research Group. Fasting glucose levels and incident diabetes mellitus in older nondiabetic adults randomized to receive 3 different classes of antihypertensive treatment: a report from the Antihypertensive and Lipid-Lowering Treatment to Prevent Heart Attack Trial (ALLHAT). Arch Intern Med. 2006 Nov 13;166(20):2191-201.

40. Barzilay JI, Davis BR, Pressel SL, Cutler JA, Einhorn PT, Black HR, Cushman WC, Ford CE, Margolis KL, Moloo J, Oparil S, Piller LB, Simmons DL, Sweeney ME, Whelton PK, Wong ND, Wright JT Jr; ALLHAT Collaborative Research Group. Long-term effects of incident diabetes mellitus on cardiovascular outcomes in people treated for hypertension: the ALLHAT Diabetes Extension Study. Circ Cardiovasc Qual Outcomes. 2012 Mar 1;5(2):153-62.

41. McAdams DeMarco MA, Maynard JW, Baer AN, Gelber AC, Young JH, Alonso A, Coresh J. Diuretic use, increased serum urate levels, and risk of incident gout in a population-based study of adults with hypertension: the Atherosclerosis Risk in Communities cohort study. Arthritis Rheum. 2012 Jan;64(1):121-9.

42. Siegel D, Hulley SB, Black DM, Cheitlin MD, Sebastian A, Seeley DG, Hearst N, Fine R. Diuretics, serum and intracellular electrolyte levels, and ventricular arrhythmias in hypertensive men. JAMA 1992;267:1083-9.

43. Mount DB, et al. Clinical manifestations and treatment of hypokalemia. UpToDate. Accessed July 2013.

44. Roush GC, Buddharaju V, Ernst ME, Holford TR. Chlorthalidone: Mechanisms of Action and Effect on Cardiovascular Events. Curr Hypertens Rep. 2013 Jul 10. [Epub ahead of print]

45. Rahman M, Pressel S, Davis BR, et al. Renal outcomes in high-risk hypertensive patients treated with an angiotensin-converting enzyme inhibitor or a calcium channel blocker vs a diuretic: a report from the Antihypertensive and Lipid-Lowering Treatment to Prevent Heart Attack Trial (ALLHAT). Arch Intern Med. 2005 Apr 25;165(8):936-46.

46. Rahman M, Ford CE, Cutler JA, et al; ALLHAT Collaborative Research Group. Long-term renal and cardiovascular outcomes in Antihypertensive and Lipid-Lowering Treatment to Prevent Heart Attack Trial (ALLHAT) participants by baseline estimated GFR. Clin J Am Soc Nephrol. 2012 Jun;7(6):989-1002.

47. Prevention of stroke by antihypertensive drug treatment in older persons with isolated systolic hypertension. Final results of the Systolic Hypertension in the Elderly Program (SHEP). SHEP Cooperative Research Group. JAMA 1991;265:3255-64.

48. Hueskes BA, Roovers EA, Mantel-Teeuwisse AK, Janssens HJ, van de Lisdonk EH, Janssen M. Use of diuretics and the risk of gouty arthritis: a systematic review. Semin Arthritis Rheum. 2012 Jun;41(6):879-89.

49. Mortality findings for stepped-care and referred-care participants in the hypertension detection and follow-up program, stratified by other risk factors. The Hypertension Detection and Follow-up Program Cooperative Research Group. Prev Med 1985;14:312-35.

50. Wilson L, Nair KV, Saseen JJ. Comparison of New-Onset Gout in Adults Prescribed Chlorthalidone vs Hydrochlorothiazide for Hypertension. J Clin Hypertens. 2014 Sep 25. doi: 10.1111/jch.12413. [Epub ahead of print]

51. Khosla N, Chua DY, Elliott WJ, Bakris GL. Are chlorthalidone and hydrochlorothiazide equivalent blood-pressure-lowering medications? J Clin Hypertens 2005;7:354-6.

52. Matthews KA, Brenner MJ, Brenner AC. Evaluation of the Efficacy and Safety of a Hydrochlorothiazide to Chlorthalidone Medication Change in Veterans With Hypertension. Clin Ther. 2013 Aug 28.[Epub ahead of print]

53. van Blijderveen JC, Straus SM, Rodenburg EM, Zietse R, Stricker BH, Sturkenboom MC, Verhamme KM. Risk of hyponatremia with diuretics: chlorthalidone versus hydrochlorothiazide. Am J Med 2014;127:763-71.

54. Roush GC, Buddharaju V, Ernst ME. Is chlorthalidone better than hydrochlorothiazide in reducing cardiovascular events in hypertensives? Curr Opin Cardiol 2013;28:426-32.

55. Dhalla IA, Mamdani MM, Juurlink DN. Chlorthalidone versus hydrochlorothiazide. Ann Intern Med. 2013;158:923-4.

56. Einhorn PT, Cushman WC, Whelton PK; ALLHAT Collaborative Research Group. Chlorthalidone versus hydrochlorothiazide. Ann Intern Med 2013;158:922-3.

57. Asche C, Yang J, Yu S, Hagan M. A profiling of hypertension patients treated with chlorthalidone or hydrochlorothiazide. Value Health. 2013 May;16(3):A295.

58. Krause T, Lovibond K, Caulfield M, McCormack T, Williams B; Guideline Development Group. Management of hypertension: summary of NICE guidance. BMJ 2011;343:d4891.

59. Mancia G, Fagard R, Narkiewicz K, et al; Task Force Members. 2013 ESH/ESC Guidelines for the management of arterial hypertension: the Task Force for the management of arterial hypertension of the European Society of Hypertension (ESH) and of the European Society of Cardiology (ESC). J Hypertens 2013;31:1281-357.

60. Floyd JS, Psaty BM. Observational Comparative Effectiveness Studies of Drug Therapies: High-Quality Answers or Important Clinical Questions? Comment on "Comparative Effectiveness of 2 β-Blockers in Hypertensive Patients". Arch Intern Med 2012:172: 1412-4.

61. Lauer MS, D'Agostino RB. The randomized registry trial — the next disruptive technology in clinical research? N Engl J Med 2013; 369:1579-1581.

62. Jansen JP, Fleurence R, Devine B, Itzler R, Barrett A, Hawkins N, Lee K, Boersma C, Annemans L, Cappelleri JC. Interpreting indirect treatment comparisons and network meta-analysis for health-care decision making: report of the ISPOR Task Force on Indirect Treatment Comparisons Good Research Practices: part 1. Value Health 2011;14:417-28.

63. Pletcher MJ, Lo B, Grady D. Informed consent in randomized quality improvement trials: a critical barrier for learning health systems. JAMA Intern Med 2014;174:668-70.

64. Peto R, Collins R, Gray R. Large-scale randomized evidence: large, simple trials and overviews of trials. J Clin Epidemiol. 1995 Jan;48(1):23-40.

65. Vickers AJ, Scardino PT. The clinically-integrated randomized trial: proposed novel method for conducting large trials at low cost. Trials. 2009;10:14.

66. van Staa TP, Goldacre B, Gulliford M, Cassell J, Pirmohamed M, Taweel A, Delaney B, Smeeth L. Pragmatic randomised trials using routine electronic health records: putting them to the test. BMJ. 2012 Feb 7;344:e55.

67. Fiore LD, Brophy M, Ferguson RE, D'Avolio L, Hermos JA, Lew RA, Doros G, Conrad CH, O'Neil JA Jr, Sabin TP, Kaufman J, Swartz SL, Lawler E, Liang MH, Gaziano JM, Lavori PW. A point-of-care clinical trial comparing insulin administered using a sliding scale versus a weight-based regimen. Clin Trials. 2011;8:183-95.

68. D'Avolio L, Ferguson R, Goryachev S, Woods P, Sabin T, O'Neil J, Conrad C, Gillon J, Escalera J, Brophy M, Lavori P, Fiore L. Implementation of the Department of Veterans Affairs' first point-of-care clinical trial. J Am Med Inform Assoc. 2012 Jun 1;19(e1):e170-e176. Epub 2012 Feb 24.

69. IOM Roundtable on Evidence-Based Medicine. Appendix A. Learning what works best: the nation's need for evidence on comparative effectiveness in health care: an issue overview. In: Learning What Works: Infrastructure Required for Comparative Effectiveness Research: Workshop Summary. Institute of Medicine (US) Roundtable on Value & Science-Driven Health Care. Washington (DC): National Academies Press (US); 2011. http://www.ncbi.nlm.nih.gov/books/NBK64784/

70. Eapen ZJ, Lauer MS, Temple RJ. The imperative of overcoming barriers to the conduct of large, simple trials. JAMA 2014;311:1397-8.

71. Smith DH, Neutel JM, Lacourcière Y, Kempthorne-Rawson J. Prospective, randomized, open-label, blinded-endpoint (PROBE) designed trials yield the same results as double-blind, placebo-controlled trials with respect to ABPM measurements. J Hypertens. 2003 Jul;21(7):1291-8.

72. Kerr EA, Lucatorto MA, Holleman R, Hogan MM, Klamerus ML, Hofer TP; VA Diabetes Quality Enhancement Research Initiative (QUERI) Workgroup on Clinical Action Measures. Monitoring performance for blood pressure management among patients with diabetes mellitus: too much of a good thing? Arch Intern Med. 2012;172:938-45.

73. Berry SD, Kiel DP. Treating hypertension in the elderly: should the risk of falls be part of the equation? JAMA Intern Med 2014;174:596-7.

74. Nord J, Stults B, Rose R, Underwood AE, Williams T, West G, Huhtala T, Milne CK. Optimizing office blood pressure measurement at a VAMC. Fed Pract 2012;29(5):35-39.

75. Kim JW, Bosworth HB, Voils CI, Olsen M, Dudley T, Gribbin M, Adams M, Oddone EZ. How well do clinic-based blood pressure measurements agree with the mercury standard? J Gen Intern Med 2005;20:647-9.

76. Freeman MK, White W, Iranikhah M. Tablet splitting: a review of the clinical and economic outcomes and patient acceptance. Consult Pharm 2012;27:421-30.

77. Kalsbeek WD, Botman SL, Massey JT, Liu PW. Cost-Efficiency and the Number of Allowable Call Attempts in the National Health Interview Survey. Journal of Official Statistics 1994;10(2): 133–52

78. Hörngren J, Lundquist P, Westling S. Effects of number of call attempts on nonresponse rates and nonresponse bias – result from some case studies at Statistics Sweden. Statistics

Canada's International Symposium Series: Proceedings, 2009. http://www.statcan.gc.ca/pub/11-522-x/2008000/article/10999-eng.pdf

79. Fowler FJ, Jr., Gallagher PM, Stringfellow VL, Zaslavsky AM, Thompson JW, Cleary PD. Using telephone interviews to reduce nonresponse bias to mail surveys of health plan members. Med Care. 2002;40:190-200.

80. Fu SS, van Ryn M, Burgess DJ, Nelson D, Clothier B, Thomas JL, Nyman JA, Joseph AM. Proactive tobacco treatment for low income smokers: study protocol of a randomized controlled trial. BMC Public Health 2014;14:337.

81. Koepsell T, McGuire V, Longstreth W, Nelson L, Belle G. Randomized trial of leaving messages on telephone answering machines for control recruitment in an epidemiologic study. Am J Epidemiol. 1996;144:704-706.

82. Andrade SE, Kahler KH, Frech F, Chan KA. Methods for evaluation of medication adherence and persistence using automated databases. Pharmacoepidemiol Drug Saf. 2006;15:565-74.

83. Thygesen K, Alpert JS, Jaffe AS, Simoons ML, Chaitman BR, White HD; Joint ESC/ACCF/AHA/WHF Task Force for the Universal Definition of Myocardial Infarction, et al. Third universal definition of myocardial infarction. Circulation. 2012;126:2020-35.

84. Systolic Blood Pressure Intervention Trial (SPRINT) Protocol

85. Braunwald E, Domanski MJ, Fowler SE, et al; PEACE Trial Investigators. Angiotensin-converting-enzyme inhibition in stable coronary artery disease. N Engl J Med 2004;351:2058-68.

86. Morrow DA, Braunwald E, Bonaca MP, et al; TRA 2P–TIMI 50 Steering Committee and Investigators. Vorapaxar in the secondary prevention of atherothrombotic events. N Engl J Med 2012;366:1404-13.

87. Tricoci P, Huang Z, Held C, et al; TRACER Investigators. Thrombin-receptor antagonist vorapaxar in acute coronary syndromes. N Engl J Med 2012;366:20-33.

88. White WB, Bakris GL, Bergenstal RM, Cannon CP, Cushman WC, et al. EXamination of cArdiovascular outcoMes with alogliptIN versus standard of carE in patients with type 2 diabetes mellitus and acute coronary syndrome (EXAMINE): a cardiovascular safety study of the dipeptidyl peptidase 4 inhibitor alogliptin in patients with type 2 diabetes with acute coronary syndrome. Am Heart J 2011;162:620-6.

89. Einhorn PT, Davis BR, Massie BM, Cushman WC, Piller LB, Simpson LM, Levy D, Nwachuku CE, Black HR; ALLHAT Collaborative Research Group. The Antihypertensive

and Lipid Lowering Treatment to Prevent Heart Attack Trial (ALLHAT) Heart Failure Validation Study: diagnosis and prognosis. Am Heart J. 2007 Jan;153(1):42-53.

90. Go AS, Mozaffarian D, Roger VL, et al; AHA Statistics Committee and Stroke Statistics Subcommittee. Heart disease and stroke statistics--2013 update: a report from the American Heart Association. Circulation 2013;127:e6-e245.

91. Kannel WB. Blood pressure as a cardiovascular risk factor: prevention and treatment. JAMA 1996;275:1571-6.

92. Roger VL, Weston SA, Redfield MM, Hellermann-Homan JP, Killian J, Yawn BP, Jacobsen SJ. Trends in heart failure incidence and survival in a community based population. JAMA 2004;292:344-50.

93. Hicks KA, Hung HMJ, Mahaffey KW, et al. Standardized definitions for cardiovascular and stroke end point events in clinical trials. FDA draft report, 2012.

94. Ron E, Carter R, Jablon S, Mabuchi K. Agreement between death certificate and autopsy diagnoses among atomic bomb survivors. Epidemiology. 1994 Jan;5(1):48-56.

95. Sesso HD, Gaziano JM, Glynn RJ, Buring JE. Value of an Endpoints Committee versus the use of nosologists for validating cause of death. Contemp Clin Trials.2006 Aug;27(4): 333-9.

96. German RR, Fink AK, Heron M, et al. The accuracy of cancer mortality statistics based on death certificates in the United States. Cancer epidemiology 2011;35(2):126-31.

97. Prieto-Merino D, Smeeth L, Staa TP, Roberts I. Dangers of non-specific composite outcome measures in clinical trials. BMJ 2013;347:f6782.

98. McKee PA, Castelli WP, McNamara PM, Kannel WB. The natural history of congestive heart failure: the Framingham study. N Engl J Med. 1971;285:1441–6.

99. Sohn MW, Arnold N, Maynard C, Hynes DM. Accuracy and completeness of mortality data in the Department of Veterans Affairs. Popul Health Metr. 2006 Apr 10;4:2.

100. Fletcher RD, Amdur RL, Kolodner R, McManus C, Jones R, Faselis C, Kokkinos P, Singh S, Papademetriou V. Blood pressure control among US veterans: a large multiyear analysis of blood pressure data from the Veterans Administration health data repository. Circulation 2012;125:2462-8.

101. ACCORD Study Group, Cushman WC, Evans GW, Byington RP, et al. Effects of intensive blood-pressure control in type 2 diabetes mellitus. N Engl J Med. 2010;362:1575-85(+ online supplement).

102. Jennison, C and Turnbull, B. Group Sequential Methods with Applications to Clinical Trials. Boca Raton, Fl: Chapman & Hall/CRC Interdisciplinary Statistics, 2000.

103. Daniels MJ and Hogan JW. Missing Data in Longitudinal Studies: Strategies for Bayesian Modeling and Sensitivity Analysis. Boca Raton, Fl: Chapman and Hall/CRC, 2008.

104. Beyersmann J, Allignol A, and Schumacher M. Competing Risks and Multistate Models with R. New York, NY: Springer, 2012.

105. http://answers.hhs.gov/ohrp/questions/7276

106. Morris MC; Nelson RM. Randomized, controlled trials as minimal risk: An ethical analysis. Crit Care Med 2007; 35:940–4.

107. Nelson K, Garcia RE, Brown J, Mangione CM, Louis TA, Keeler E, Cretin S. Do patient consent procedures affect participation rates in health services research? Med Care 2002;40:283-8.

108. Cann CI, Rothman KJ. IRBs and epidemiologic research: how inappropriate restrictions hamper studies. IRB 1984;6(4):5-7.

109. Armstrong D, Kline-Rogers E, Jani SM, Goldman EB, Fang J, Mukherjee D, Nallamothu BK, Eagle KA. Potential impact of the HIPAA privacy rule on data collection in a registry of patients with acute coronary syndrome. Arch Intern Med 2005;165:1125-9.

110. Kaiser J. Patient privacy. Rule to protect records may doom long-term heart study. Science 2006;311:1547-8.

111. Ellickson PL, Hawes JA. An assessment of active versus passive methods for obtaining parental consent. Eval Rev 1989;13:45-55.

Department of Veterans Affairs Cooperative Studies Program

CSP#597: Diuretic Comparison Project (DCP)

A proposal from the Point of Care Program

Study Chair: Areef Ishani, MD

Version 4.4

This document contains confidential material that is privileged. Please do not disseminate this document, or sections thereof, beyond its limited original distribution

VA Central IRB

Table of Contents

I. Abbreviations and Acronyms 4

II. Study Question 7

III. Background and Rationale 7

IV. The Point of Care Program within the VA's Office of Research and Development 14

V. Relevance of our trial design to current needs of VA healthcare 16

VI. Study Design 18

VII. Study Population 18

VIII. Study Intervention 19

IX. Study Procedures 19

X. Outcome Measures 24

XI. Biostatistical Considerations 31

XII. Feasibility and Recruitment Plan 37

XIII. Data Collection and Data Sources 38

XIV. Data Management and Data Security Plans 38

XV. Human Subjects 41

XVI. Quality Control Procedures 43

XVII. Study Monitoring Plan 44

XVIII. Study Organization and Administration 46

XIX. Publications 49

XX. References 51

A. **Executive Summary**

Thiazide-type diuretics have been in use for more than 50 years and are considered in JNC-7 and VA guidelines to be the first-line treatment for hypertension. Of the more than 1 million veterans prescribed a thiazide-type diuretic each year, more than 95% receive hydrochlorothiazide, and fewer than 2.5% receive chlorthalidone. However, indirect evidence has been accumulating for many years that chlorthalidone may be more effective than hydrochlorothiazide at preventing cardiovascular events, by about 20% according to a recent network meta-analysis. Possible mechanisms for such an effect include longer duration of action, better nighttime blood pressure control, and pleiotropic effects of chlorthalidone. A randomized trial comparing the effect of the two drugs on cardiovascular outcomes has never been conducted, primarily for reasons of cost.

We are proposing a new type of efficient, less expensive randomized trial (termed a “clinically integrated” or “point of care” trial) to answer the question of whether chlorthalidone is more effective than hydrochlorothiazide at preventing cardiovascular outcomes in older patients with hypertension. Our primary outcome will be a composite consisting of: stroke, myocardial infarction, non-cancer death, urgent coronary revascularization, and hospitalization for acute congestive heart failure. We plan to enroll patients over age 65 years currently prescribed hydrochlorothiazide 25 or 50 mg daily with no recent systolic blood pressure below 120 mm Hg, and randomize them to either continue on hydrochlorothiazide or receive open-label chlorthalidone at suggested doses of 12.5 or 25 mg, respectively.

To have a 90% power with 2-sided $\alpha = 0.05$ to detect a 17.5% reduction in the expected 3% per year primary outcome occurrence rate in the hydrochlorothiazide group, we plan to randomize 13,700patients over 3 years and follow them for a mean of 3 years, for a total study duration of 4.5 years.

The key feature of our design is that, instead of employing local investigators, we substitute centralized study processes and rely on usual primary care. Specifically, this involves: 1) identification of eligible patients using the VA electronic medical record system (EMR), 2) centralized recruitment and enrollment, involving permission from the patient’s primary care provider, a patient recruitment letter, and informed consent obtained by telephone, 3) centralized placement of notes and orders using the VA EMR, 4) all patient care including the study drug to be managed by the primary care provider, and 5) centralized passive collection of outcomes and process variables using the VA EMR, Medicare, and other national VA and non-VA databases.

I. Abbreviations and Acronyms

ACCOMPLISH	Avoiding Cardiovascular Events in Combination Therapy in Patients Living with Systolic Hypertension
ACCORD	Action to Control Cardiovascular Risk in Diabetes Trial
ACE	Angiotensin Converting Enzyme
ACEI	Angiotensin Converting Enzyme Inhibitors
ACME	Automated Classification of Medical Entities
AHA	American Heart Association
ALLHAT	Antihypertensive and Lipid-Lowering Therapy to Prevent Heart Attack Trial
ANBP2	Second Australian National Blood Pressure Study
ARB	Angiotensin Receptor Blocker
ARIC	Atherosclerosis Risk in Communities
BIRLS	Beneficiary Identification and Records Locator Subsystem database
CAC	Clinical Application Coordinator
CBOC	Community Based Outpatient Clinics
CDW	Corporate Data Warehouse
CFR	Code of Federal Regulations
CHD	Coronary Heart Disease
CHF	Congestive Heart Failure
CI	Confidence Interval
CPRS	VA Computerized Patient Record System
CPT	Current Procedural Terminology
CSP	Cooperative Studies Program
CSPCC	Cooperative Studies Program Coordinating Center
CSSEC	Clinical Sciences Scientific Evaluation Committee
CVD	Cardiovascular Disease

DM	Diabetes Mellitus
DMC	Data Monitoring Committee
DoD	US Department of Defense
ECG	Electrocardiography
EMR	Electronic Medical Record
EHR	Electronic Health Record
EXAMINE	EXamination of cArdiovascular outcoMes with alogliptIN versus standard of carE in patients with type 2 diabetes mellitus and acute coronary syndrome
FDA	US Food and Drug Administration
HCTZ	Hydrochlorothiazide
HDFP	Hypertension Detection and Follow-up Program
HF	Heart Failure
HIPAA	Health Insurance Portability and Accountability Act
HR	Hazard Ratio
ICD-9	International Classification of Disease, 9th edition
ICD-10	International Classification of Disease, 10th edition
ICD	Internal Cardiac Defibrillator
IRM	Information Resource Management
ISO	Information Security Officer
JNC-7	Seventh Report of the Joint Committee on Prevention, Detection, Evaluation and Treatment of High Blood Pressure
meq/L	Milli-equivalents per liter
mg	Milligram
MI	Myocardial Infarction
mmH	Millimeters of Mercury
MRFIT	Multiple Risk Factor Intervention Trial

NHLBI	National Heart, Lung, Blood Institute
NIH	National Institutes of Health
PACT	Patient Aligned Care Team
PBM	VA Pharmacy Benefits Management
PCP	Primary Care Provider
PEACE Trial	Prevention of Events with Angiotensin-Converting Enzyme Inhibition
PHI	Protected Health Information
POC-CT	Point of Care Clinical Trial
PROBE	Prospective, Randomized, Open-label, Blinded-Endpoint Trial
SBP	Systolic Blood Pressure
SHEP	Systolic Hypertension in the Elderly Population Trial
SPRINT	Systolic Blood Pressure Intervention Trial
SOP	Standard Operating Procedure
SME	Subject Matter Expert
TOMHS-T	Treatment of Mild Hypertension Study
TRACER	Thrombin-Receptor Antagonist Vorapaxar in Acute Coronary Syndromes
(TRA2°P)-TIMI 50	Thrombin Receptor Antagonist in Secondary Prevention of Atherothrombotic Ischemic Events Trial
UK NICE	United Kingdom National Institute for Health and Care Excellence
VA	US Department of Veterans Affairs
VAMC	VA Medical Center
VIREC	VA Information Resource Center
VISN	Veterans Integrated Service Network
WHO	World Health Organization

II. Study Question

Does treatment with chlorthalidone reduce cardiovascular outcomes compared with hydrochlorothiazide in older patients with hypertension?

III. Background and Rationale

A. Diuretics for hypertension

Hypertension is the most common primary diagnosis in America (1), and 3 of the 10 most commonly prescribed drugs in the US are antihypertensive agents (2). Thiazide-type diuretics are recommended as first line therapy by VA/DoD hypertension guidelines (3) and by the Seventh Report of the Joint Committee on Prevention, Detection, Evaluation, and Treatment of High Blood Pressure (JNC-7). JNC-7 noted that “diuretics have been virtually unsurpassed in preventing complications of hypertension” and as a result “should be used in drug treatment for most patients with uncomplicated hypertension” (4). In a network meta-analysis of 42 trials involving 192,478 patients randomized to active drug treatment for hypertension vs. placebo, low-dose diuretics (usually hydrochlorothiazide or the thiazide-type diuretic chlorthalidone) were “the most effective first-line treatment for preventing the occurrence of cardiovascular disease morbidity and mortality” (5).

B. Hydrochlorothiazide and chlorthalidone

Nearly all thiazide-type diuretic prescriptions in the U.S. are for hydrochlorothiazide. Hydrochlorothiazide is among the top 10 most commonly prescribed drugs in the US (2), with 135 million prescriptions written annually (6). In the VA national outpatient prescription database, of the more than 1 million veterans prescribed a thiazide-type diuretic each year from 2003-8, more than 95% received hydrochlorothiazide, and fewer than 2.5% received chlorthalidone (7). From our own search of the subsequent 3-year period 2009-11, 1.5 million veterans received hydrochlorothiazide from the VA and 50,000 received chlorthalidone.

The nearly universal use of hydrochlorothiazide has been attributed to a variety of factors, including 1) early aggressive marketing by its manufacturer (Merck) using “the largest pharmaceutical sales force in the world” (8), 2) its use in the early landmark VA hypertension treatment trials, 3) its early and frequent inclusion into combination pills, numbering at least 28 preparations (8), and 4) the ease of abbreviation to “HCTZ”, which may influence physician preference (9).

Both drugs have been approved by the FDA and in use for more than 50 years, have long been available as generics, and are included in the VA national formulary in the same drug class: USP code CV 701, thiazide-related diuretics. There is no patient characteristic that influences the choice between these two drugs - it is based solely on physician preference.

C. Drug dosages

Both drugs were once commonly used at doses of 100 mg per day and higher, but by 1990, concern over the lack of reduction in coronary events from blood pressure reduction by thiazides (despite proven

efficacy for stroke), and further concern that this might be due to competing harms (particularly ventricular arrhythmias) from thiazide-induced metabolic abnormalities, led to recommendations to use lower doses of diuretics, including suggestions to use 12.5 mg of hydrochlorothiazide (10).

In the early 1990's, publications of randomized dosing trials from the VA cooperative study group concluded that the doses of 25-50 mg of hydrochlorothiazide were nearly as effective as higher doses at controlling blood pressure with fewer adverse metabolic effects, favoring the use of 25 mg and a maximum dose of 50 mg (11,12). Several randomized dosing trials of chlorthalidone found 12.5 mg per day to be effective, and found 25 mg per day to be nearly as effective as higher doses with fewer adverse metabolic effects (13,14). Since then, doses of 12.5-25 mg per day have been widely used (15) and rarely exceeded for both drugs, with higher doses accounting for only 7% of VA prescriptions in 2008 (7). However, several authors have pointed out that hydrochlorothiazide doses lower than 25 mg have not been shown to reduce cardiovascular outcomes and have performed poorly in randomized trials (6). As a result, JNC-8 recommends target doses of 25-50 mg per day for hydrochlorothiazide and 12.5-25 mg per day for chlorthalidone (16).

D. MRFIT – the first evidence that chlorthalidone may be more effective

Despite the near universal use of hydrochlorothiazide in the U.S., evidence has accumulated over the past 30 years suggesting that chlorthalidone may be more effective at reducing cardiovascular outcomes. The first indication was from the Multiple Risk Factor Intervention Trial (MRFIT), a large randomized trial of a multi-component 'special intervention' to prevent cardiovascular events in which either hydrochlorothiazide or chlorthalidone could be used as first-line treatment of hypertension in the special intervention arm. During the study, clinics that used hydrochlorothiazide were noted to have 44% more coronary heart disease deaths than those using chlorthalidone. In 1980, the MRFIT Policy Advisory Board changed the protocol, recommending chlorthalidone over hydrochlorothiazide for initial therapy, and lowered the maximum dose to 50 mg. Mortality in the former hydrochlorothiazide clinics subsequently dropped 28% (which could, of course, partially reflect regression to the mean). A recent analysis of the use of chlorthalidone and hydrochlorothiazide within MRFIT reported significantly fewer cardiovascular events with chlorthalidone, though the findings of this non-randomized comparison are confounded by large differences in dosage, randomized group, and lipid lowering (17). A separate non-randomized analysis of MRFIT data concluded that chlorthalidone was associated with less left ventricular hypertrophy than hydrochlorothiazide (18).

E. Indirect comparisons of randomized trial data

Because of the MRFIT observations, most subsequent NIH-funded blood pressure trials have used chlorthalidone, including the Hypertension Detection and Follow-up Program (HDFP), the Systolic Hypertension in the Elderly Program (SHEP), the Treatment of Mild Hypertension Study (TOMHS), and the Antihypertensive and Lipid-Lowering Treatment to Prevent Heart Attack Trial (ALLHAT), and in the ongoing Systolic Blood Pressure Intervention Trial (SPRINT), it is 'preferred'. The use of chlorthalidone in these trials and of hydrochlorothiazide in many other trials has enabled indirect comparisons of the effects of the two drugs against a third drug or class. Thus, hydrochlorothiazide resulted in worse

outcomes than an ACE inhibitor (enalapril) in men in the Second Australian National Blood Pressure Study (ANBP2) (19), and worse outcomes than amlodipine (with all patients receiving benazepril) in the Avoiding Cardiovascular Events in Combination Therapy in Patients Living with Systolic Hypertension (ACCOMPLISH) trial (6), whereas in ALLHAT, chlorthalidone was found to be superior to an ACE inhibitor (lisinopril) or amlodipine “in preventing 1 or more major forms of CVD” (though there was no difference in the primary outcome) (20). These and other indirect comparisons have recently been combined into a focused network meta-analysis that estimated a 21% risk reduction ($p < .0001$) in cardiovascular events with chlorthalidone relative to hydrochlorothiazide that persisted (as 18%, $p=.024$) in an analysis adjusted for attained blood pressure (21).

F. Relative Potency

Several studies have found chlorthalidone to have about twice the potency of hydrochlorothiazide (22,23,24) and this is reflected in the VA Pharmacy Benefits Management (PBM) 2009 evidence review (25). Two indirect meta-analyses have reported similar conclusions. Ernst et al (26) found that at identical doses, chlorthalidone had a greater effect than hydrochlorothiazide on lowering blood pressure. Pederzan et al (27) considered the 2 drugs to have equivalent effects at equally potent doses, which they considered to be 3 to 1. Despite these indications of greater potency, in practice chlorthalidone is not used in lower doses than hydrochlorothiazide (7,28,29). This may reflect greater awareness of appropriate thiazide dosing by the small proportion of prescribers who use chlorthalidone, rather than widespread belief that the potencies are equivalent. There is essentially universal agreement among experts that chlorthalidone at 12.5 or 25 mg is equipotent to hydrochlorothiazide at 25 or 50 mg (respectively).

G. Possible differences between the two diuretics

No randomized trials have been conducted that directly compare clinical (e.g., cardiovascular) outcomes of chlorthalidone and hydrochlorothiazide, but several short-term randomized trials have examined blood pressure and metabolic effects. Two recent short-term (10 and 12 weeks) randomized double-blind trials of 609 and 1071 patients (respectively) compared blood pressure control and adverse effects when taking chlorthalidone 25 mg or hydrochlorothiazide 25 mg in patients also taking an angiotensin receptor blocker (ARB) (30,31). In both studies, chlorthalidone resulted in lower systolic blood pressure by at least 5 mmHg in clinic and, in the larger study, by 24 hour ambulatory recording.

Chlorthalidone is known to have a longer duration of action than hydrochlorothiazide. The elimination half-life of chlorthalidone is 50-60 hours compared with 9-10 hours for hydrochlorothiazide (32). Ernst et al (23) randomized 30 patients to either chlorthalidone 12.5mg/day, titrated to 25 mg/day or hydrochlorothiazide 25 mg/day, titrated to 50mg/day. After 8 weeks, there was no significant difference in office systolic blood pressure (SBP), but 24-h ambulatory SBP favored chlorthalidone over hydrochlorothiazide: -12.4 (± 1.8) vs. -7.4 (± 1.7) mmHg, respectively, P = 0.054, an effect primarily driven by the lower nighttime SBP for chlorthalidone compared with hydrochlorothiazide: -13.5 (± 1.9) and -6.4 (± 1.8) mmHg, respectively, P = 0.009. Of note in this regard, large observational studies have

found nighttime blood pressure to be a better predictor of cardiovascular outcomes than daytime blood pressure (33,34).

A recent in vitro study reported that chlorthalidone reduced epinephrine-induced platelet aggregation and increased angiogenesis more than the thiazide bendroflumethiazide (35). There have been no reports of any related clinical effects (e.g., increased bleeding), but these mechanisms could help to explain differences between thiazides in reducing vascular events (36).

In summary, given the longer duration of action, better nighttime blood pressure control, and possible pleiotropic effects of chlorthalidone, it is possible that chlorthalidone and hydrochlorothiazide could have different long-term effects on cardiovascular outcomes even at doses that result in similar office blood pressures.

H. Drug costs

Although both drugs are inexpensive generic products, chlorthalidone costs the VA seven times as much as hydrochlorothiazide. According to VA Pharmacy Benefits Management (PBM), the cost to the VA for hydrochlorothiazide 50 mg is 1.6¢ per tablet and for chlorthalidone 25mg is 11¢ per tablet (with half doses costing half as much). Thus if the approximately 1 million VA patients using hydrochlorothiazide (nearly all on 12.5 or 25 mg) were switched over to chlorthalidone 12.5 mg at an additional cost of 5¢ per day, the total increased cost would be about $18 million per year.

I. Comparative metabolic effects

Thiazide diuretics have a variety of metabolic effects. They generally lower serum sodium (36) and potassium (27,38) levels and increase blood sugar (39,40) and uric acid (27,41) levels. In a small randomized trial (42), hydrochlorothiazide 50 mg per day lowered potassium by a mean of 0.44 meq/L more than placebo, regardless of whether potassium supplements or triamterene were added. Perhaps the best source of information on the effect of thiazides comes from ALLHAT, which randomized 33,357 patients with hypertension: 15,255 to chlorthalidone and 9000 each to amlodipine and lisinopril. Overall, chlorthalidone was considered superior to the other drugs in preventing cardiovascular events (20). The year-1 incidence of hypokalemia (<3.5 meq/L) was higher with chlorthalidone (12.9%) than with lisinopril (1.0%) or amlodipine (2.1%), but only 3.5% of the chlorthalidone group had a level <3.2 meq/L, and, in the chlorthalidone group, hypokalemia was associated with fewer cardiovascular outcomes than was normokalemia (38).

According to UpToDate (43): "The severity of the manifestations of hypokalemia tends to be proportionate to the degree and duration of the reduction in serum potassium. Symptoms generally do not become manifest until the serum potassium is below 3.0 meq/L, unless the serum potassium falls rapidly or the patient has a potentiating factor, such as a predisposition to arrhythmia due to the use of digitalis. Symptoms usually resolve with correction of the hypokalemia. … Muscle weakness usually does not occur at serum potassium concentrations above 2.5 meq/L if the hypokalemia develops slowly. … In addition to causing muscle weakness, severe potassium depletion (serum potassium less than 2.5 meq/L) can lead to muscle cramps, rhabdomyolysis, and myoglobinuria." In one study, occurrence of

premature ventricular contractions was twice as common when serum potassium was below 3.0 meq/L (43).

In ALLHAT (39,40), chlorthalidone was also associated with more incident diabetes (defined as any fasting blood sugar >125 mg/dL) than were the other drugs (chlorthalidone: 14%, amlodipine: 11.1%, lisinopril: 9.5%). While overall, those with incident diabetes had more cardiovascular deaths, incident diabetes in the chlorthalidone group had lower cardiovascular deaths than incident diabetes in other groups, leading the ALLHAT investigators to conclude that “there is no conclusive or consistent evidence that this diuretic-associated increase in DM risk increases the risk of clinical events” (39), so “concerns regarding potential adverse diabetic effects associated with thiazide-type diuretic therapy should not inhibit its use” (40). Roush et al (44) recently reviewed this literature and concluded that “Chlorthalidone-induced diabetes mellitus (DM) is “chemical diabetes” rather than DM leading to cardiovascular pathology.”

Also in ALLHAT, chlorthalidone did not increase the rate of development of either end-stage renal disease or of a 50% or greater decrease in glomerular filtration rate compared with lisinopril or amlodipine (45,46).

Sodium, uric acid, and gout were not followed in ALLHAT. Thiazides have been associated with hyponatremia in observational studies. In the Rotterdam study, about 50 of 3400 patients treated with thiazides over 6 years developed Na < 130 meq/L, 4.5 times as many as controls (36). However, in SHEP, patients randomized to chlorthalidone did not differ from those receiving placebo in sodium levels after 1 year (47).

Similarly, in the observational Atherosclerosis Risk in Communities (ARIC) study, thiazides were associated with an increased risk of incident gout mediated by increased uric acid levels (41). A recent systematic review concluded “There is a trend toward a higher risk for acute gouty arthritis attacks in patients on loop and thiazide diuretics, but the magnitude and independence is not consistent. Therefore, stopping these useful drugs in patients who develop gouty arthritis is not supported by the results of this review” (48). In HDFP, patients randomized to chlorthalidone had reduced mortality compared with usual care regardless of baseline uric acid level (49). In a matched sample comparison of national pharmacy records, new onset gout episodes occurred with similar frequency in the year following prescription for chlorthalidone or hydrochlorothiazide, despite the 2 drugs being used in equal milligram doses (50).

The above comparisons involving thiazide vs. no thiazide demonstrate effects that are minor and of uncertain clinical importance. In addition, two studies from university (51) and VA (52) settings changed 19 and 40 patients (respectively) on a stable dose of hydrochlorothiazide to an equal milligram dose of chlorthalidone. Both reported reduced blood pressure with no significant metabolic effects except for one instance of hypokalemia in the university study.

Changing from hydrochlorothiazide to a roughly equipotent half dose of chlorthalidone (the intervention proposed in this study) should result in even smaller effects. In the randomized study by Ernst et al (23), hypokalemia < 3.5 meq/L occurred in nearly identical proportions at 2:1 doses: 50% of patients on

hydrochlorothiazide 25/50mg and 46% on chlorthalidone 12.5/25mg. Within the commonly used dosing range of 12.5-25 mg per day, potassium reduction was found to be equivalent for the 2 drugs in one meta-analysis (26), whereas the other found it to mirror the potency results, i.e. greater for chlorthalidone at equal milligram doses (27). In a Dutch population-based case-control study, hyponatremia was twice as common with chlorthalidone compared with hydrochlorothiazide at equal doses, but no difference was observed comparing 2:1 dosing (53). In the short-term randomized trials of 609 and 1071 patients that compared equal doses (25 mg) of the 2 drugs in patients also taking an angiotensin receptor blocker, hypokalemia was rare, occurring in 1-2% (30,31). Summarizing the metabolic data available for the 2 drugs, a recent review (54) concluded that “factors such as serum potassium, glucose, lipids, endothelial function, and oxidative status” “are either favorable to chlorthalidone or are neutral in arriving at a decision as to which drug is superior.”

More recently, a population-based observational study from Ontario compared effects of starting treatment with chlorthalidone (10,384 patients) vs. hydrochlorothiazide (propensity matched sample of 19489 patients) with a mean follow-up of about one year (29). Patients treated with chlorthalidone received higher doses (despite its greater potency), and were less likely to also be treated with an ACEI or ARB (drugs that raise potassium levels). Chlorthalidone was associated with a small non-significant reduction in the composite cardiovascular outcome, from 3.4 to 3.2 per 100 patient-years (adjusted HR 0.93, CI: 0.81-1.06). However, treatment with chlorthalidone was associated with significantly more hospitalizations with (not necessarily “for”) hypokalemia (0.69 vs 0.27 events per 100 patient-years, adjusted HR 3.06, CI: 2.04-4.58) and hyponatremia (0.69 vs 0.49 events per 100 patient-years, adjusted HR 1.68, CI: 1.24-2.28). The authors included hospitalizations that listed electrolyte abnormalities as secondary diagnoses noted during hospitalizations for other reasons. Hypokalemia and hyponatremia were each recorded as a secondary outcome noted during hospitalization less than once per 100 patient-years. In response to a letter suggesting that the analysis should be restricted to hospitalizations “for” hypokalemia (as primary diagnosis), the authors responded that doing so would result in so few hospitalizations that “such an analysis would be severely underpowered” (55). So while it is not known whether chlorthalidone caused more hospitalizations “for” hypokalemia, it is clear that such hospitalizations were rare.

The Ontario study had the advantages of large size and direct comparison of the two drugs in a population. The principal disadvantages were the observational design and the very small (and therefore potentially quite different) proportion taking chlorthalidone. Incomplete adjustment for known confounders (e.g., for dose and co-treatment) or from unrecognized confounders in the treated populations could have influenced the findings, as noted in a letter by the ALLHAT investigators (56). For example, chlorthalidone is likely used more often by hypertension specialists who might have been more attentive to recording electrolyte abnormalities on discharge summaries. Review of US data indicates that chlorthalidone is used in patients with more severe co-morbidities than those given hydrochlorothiazide (57). The Ontario study nevertheless raises questions regarding the possible superiority of chlorthalidone. In the correspondence following its publication, both the ALLHAT investigators and the Ontario authors stress the need for a randomized trial, such as the one we are proposing, to resolve this uncertainty (55,56).

In summary, the metabolic effects of thiazides are minor and have little or no clinical effect. Evidence from a variety of studies indicates that substitution of an equipotent dose of chlorthalidone for hydrochlorothiazide can be expected to have no more metabolic effect than might occur if the patient remained on hydrochlorothiazide without the substitution. **This is the basis for our assertion that this substitution constitutes minimal risk**.

J. Expert views on the choice of drugs and the need for a randomized trial

The evidence summarized above is consistent with a substantial benefit from using chlorthalidone rather than hydrochlorothiazide, but is not compelling, as the Ontario study illustrates. Many of the studies describing a possible advantage of chlorthalidone were conducted by Ernst and colleagues at the University of Iowa (7,18,22,23,26,28), but those authors have stated that "we do not believe there is strong evidence to support the use of chlorthalidone over HCTZ" (22). Ernst and Marvin Moser (who pioneered thiazide use in the 1950's) wrote in 2009: "Whether hydrochlorothiazide and chlorthalidone are interchangeable in reducing the risk of cardiovascular events is questionable"(32). On the other hand, Messerli et al reviewed the literature and concluded that "there is no evidence showing that HCTZ in the dose of 12.5-25 mg reduces myocardial infarction, stroke, or death" and argue that "if a thiazide-type diuretic is indicated, either chlorthalidone or indapamide should be selected" (6). The 2011 UK National Institute for Health and Care Excellence (NICE) guidelines for hypertension (58) recommend "a thiazide like diuretic, such as chlorthalidone…, in preference to a conventional thiazide diuretic such as bendroflumethiazide or hydrochlorothiazide", whereas the 2013 European Society guidelines dispute this and conclude that "no recommendation can be given to favor a particular diuretic agent" (59).

A recent review from investigators at the New Mexico VA (15) concludes: "The available evidence therefore supports both HCTZ and chlorthalidone as safe and effective drugs for treating hypertension. Although there are favorable trends both in terms of antihypertensive efficacy as well as clinical outcomes data with chlorthalidone compared with HCTZ, the results are not conclusive, and as such may not be enough to shift the treatment paradigm in favor of chlorthalidone, given the comfort level that most prescribers have with HCTZ. A head-to-head study looking at hard clinical outcomes, which may or may not ever be performed, may be the only way to resolve the ongoing debate as to which is the preferred thiazide for treating hypertension." Floyd and Psaty noted in 2012 (60) that "In the area of pharmacological drug treatment for high blood pressure, the current question of primary interest is whether health outcomes associated with the use of hydrochlorothiazide and chlorthalidone may differ. …Reliable and valid comparisons between hydrochlorothiazide and chlorthalidone will require a large, long-term clinical trial."

As suggested by the New Mexico authors, it is extremely unlikely that a randomized clinical outcomes trial of hydrochlorothiazide vs. chlorthalidone will ever be undertaken except by the inexpensive methodology we are proposing. Neaton and Grimm, University of Minnesota Professors who participated in MRFIT, SHEP, and subsequent NIH hypertension trials, have been lobbying for such a trial since the end of SHEP more than 20 years ago. They have discussed the idea with the NHLBI project office numerous times over conference calls and had a meeting in Bethesda about this issue. According to Dr. Grimm, NHLBI project officers have maintained that they will only consider a 5 year proposal

capped at $1.5 million a year. The Director of the NHLBI Cardiovascular division has recently written that "we can no longer afford to undertake randomized effectiveness trials that cost tens or hundreds of millions of dollars" (61). Apart from a trial design such as we are proposing, which may be unique to the VA system, these investigators found it impossible to design a study for an amount close to this budget. Richard Grimm concluded a 7/29/13 email to the principal proponent (FAL) with: "It looks like the last chance of getting a definitive answer for this incredibly important question is your VA proposal."

K. Summary of evidence and potential impact of the proposed trial

Direct evidence shows chlorthalidone to be more potent and longer-lasting than hydrochlorothiazide, and indirect interventional evidence from the Roush network meta-analysis (21) suggests that chlorthalidone may have a more beneficial effect on cardiovascular events, whereas the large Ontario observational study (29) did not find a significant reduction in adverse cardiovascular events. In comparing these 2 methodologies, the International Society for Pharmacoeconomics and Outcomes Research Indirect Treatment Comparisons Good Research Practices Task Force concluded that "a network meta-analysis must be considered observational evidence, but is arguably less prone to confounding bias than an observational comparative (prospective) cohort study" (62).

Currently available data thus favor a likely substantial benefit from chlorthalidone compared with hydrochlorothiazide, but these data have had little effect on prescribing, which continues to overwhelmingly favor hydrochlorothiazide.

If cardiovascular events were reduced by even a small amount by chlorthalidone, the public health effect would be considerable because of the large number of patients who take diuretics. For the VA, it would likely justify the effort and cost of implementing a national policy to change drugs. However, the evidence is not yet persuasive enough to justify active measures directed at increasing chlorthalidone use. A randomized head-to-head comparison of the effectiveness of these two drugs at reducing cardiovascular events is clearly necessary to determine whether chlorthalidone is superior and if so, to justify efforts to change practice.

IV. The Point of Care Program within the VA's Office of Research and Development

A. Program Background

Medical decision making is informed by clinical trials and observational studies. While clinical trials are the gold standard in clinical research the high cost of conducting these studies combines with issues of generalizability to limit their contribution to changes in medical practice. Observational studies are far less expensive and thus more numerous, addressing a broader scope of clinical issues. However, their primary failing is that the lack of randomization often leaves open the possibility of bias due to selection by indication, and residual confounding of results due to unobserved prognostic factors that influence treatment decisions.

Point of care (POC) randomization represents an intermediate strategy between these two approaches. The intent is to introduce the opportunity to randomize patients at decision points in clinical care where

two or more alternatives are considered equivalent on average by the medical community (that is, clinical equipoise exists). Patients who agree to be randomly assigned to treatment options will become subjects in the clinical experiment.

POC randomization preserves the experimental quality of clinical trials without the cost of the clinical trial apparatus; recruitment and randomization is done at the point of care with minimal perturbation of work flow, and outcomes are assessed by automated extraction of data from the medical record. Reduction of the need for research staff interaction with potential subjects (limited to obtaining informed consent) greatly reduces cost and generates data that reflects the *effectiveness* (rather than efficacy) of experimental interventions in clinical care.

To be effective, the additional burden on patients and health care providers imposed by POC randomization must be minimal. The VA electronic medical record system (VistA) can be customized to identify, enroll and randomize patients and serve as the source of outcomes data and is thus well suited for such a trial design. Subject recruitment and enrollment will be accomplished by embedding processes within the VistA system through a series of dialog boxes. Furthermore, an external system will extract consent and randomization data from the databases supporting VistA.

As noted in a recent editorial (63), "With optimal use of EMRs, the administrative costs of a trial need not increase with the sample size; this decoupling of costs and size facilitates large, simple, and inexpensive trials that have the potential to transform health systems into entities that learn and continuously improve."

As the VA system continues to lead in the development of EMR that support increasingly sophisticated monitoring for outcome-based evaluation of care, we anticipate that the methods we test using POC randomization will have even greater scope for application. The methods are also a natural fit for testing personalized and precision medicine strategies and for experimental comparative effectiveness research.

B. POC Pilot Study

The first implementation of POC randomization is a pilot study that was conducted at 3 VAMC across 2 VISNs. The goal of the pilot was fivefold:

1. To test the feasibility of the method for modification of VistA/CPRS screens and the ability to randomize within the system;
2. To assess patient and provider acceptance of the new methodology;
3. To assess the regulatory acceptance of:
    a. Informed consent procedures;
    b. Safety monitoring;
    c. Ethical considerations;
4. To test the method for data extraction and passive collection of endpoint data using the EMR;
5. To apply Informatics techniques to refine and improve efficiencies in the identification of endpoints, etc.

The pilot is a comparison of two standard strategies of insulin administration for hospitalized patients and is designed as an open-label, randomized trial comparing sliding scale regular insulin (ssRI) to a weight based regimen for control of hyperglycemia in non-ICU inpatients. The strategy is to enroll patients into the study directly from the point of contact with clinical care within all inpatient facilities. All non-ICU patients who require in-hospital insulin therapy are eligible for this study. Clinicians decide at the time of care (through the VistA order entry screen for insulin) whether or not they will allow their patient to be contacted by POC study staff. If the treating clinician agrees, consenting patients are randomized to treatment arms and treated by their clinicians according to usual practices. All technical modifications necessary are executed within the "Clinical Alert" package. Consenting patients are then followed through VistA from randomization until 30 days post discharge. Comparisons of effectiveness will be formally conducted using length of hospital stay measured in days as our primary outcome measure.

The pilot has demonstrated the feasibility of the POC method as an alternative design to traditional trials. To date, 92% of eligible patients have accepted participation and have been consented into the pilot protocol. Additionally, 71% of all clinicians that are able to order medications have accepted randomization within the clinic and have referred their patients to the protocol. All patient data has been extracted from the EMR and has been validated with chart review by a qualified clinician. There have been no significant safety events and no findings from regulatory audits or the local institutional review boards.

C. The POC National Program

The goal of POC program is to deliver state of the art treatments to patients simultaneously with enrolling them as subjects to redefine that care. By institutionalizing a process of statistically sound and efficient learning, and by integrating that learning with automatic implementation of best practice, the participating VA health care systems will accelerate improvements in the effectiveness of care for veterans. With this goal in mind, Point of Care Research has been designated as one of the Secretary's (SECVA) Transformational Initiatives within the VA and within the Office of Research and Development (ORD). Accordingly, this initiative has a national scope and an operating budget of approximately $10.2Million (exclusive of study budgets).

The Boston CSPCC has been tasked with leading the effort to implement POC randomization within the VA. To that end, our mission is to build the infrastructure necessary to support POC research; to educate providers, veterans and investigators on the initiative; to build consensus for support of the initiative among the VA community as a whole; and to explore the ethical, scientific, and regulatory aspects of the POC method itself. In order to fulfill its mission, the Boston CSPCC collaborates with national leaders in the fields of medical ethics and pragmatic clinical trials to produce scholarly works and it will conduct focus groups of veterans, veteran service organizations, and providers in order to develop a national educational campaign for all VA stakeholders.

V. Relevance of our trial design to current needs of VA healthcare

The most reliable way to learn if medical interventions provide more benefit than harm for our patients is through large randomized trials (64). However, large randomized trials that enroll thousands of patients can cost hundreds of millions of dollars, placing them out of range for most payers, including VA. As a result, many important questions remain unanswered.

We are proposing herein a ‘clinically integrated’ study design (65,66) that will incorporate and extend previously described VA ‘Point of Care’ Clinical Trial methodology (67,68). This is an efficient and inexpensive design, that will rely on a centralized processes involving mail, phone, and data extraction from the VA electronic medical records (EMR) , assisted by a designated study champion at each site (the Site Liaison) and by local Clinical Application Coordinators (CACs). These methods will allow us to avoid having to employ study personnel at each site to manage patients and collect data. As a result, the infrastructure costs typically dedicated to these activities can be reinvested to answer other questions for the healthcare system. The goal, as Lauer and D’Agostino suggest, is “to design and conduct megatrials with what we have: bigger data and smaller budgets” (61).

The following quotes, excerpted from the 2011 Institute of Medicine report “Learning what works best - the nation’s need for evidence on comparative effectiveness in health care”(69), provide support for our study objectives and design:

“A core objective for the nation is achieving the best health outcome for every patient. This objective simply cannot be accomplished until we have better evidence on which to base healthcare decisions”; “the most rapidly growing problem is that of our inability to produce the needed evidence in a timely fashion”; “Estimates of the proportion of medical care in the United States that is based on, or supported by, adequate evidence range widely. However, given concerns about the extent to which this information may be generalized, and the quality of the evidence which is used, some place this figure at well below half.”; “Within the overall umbrella of clinical effectiveness research, the most practical need is for studies of comparative effectiveness, the comparison of one diagnostic or treatment option to one or more others.”; “issues in need of additional systematic evaluation … include issues related to the comparative evaluation of different drugs within a single class”; “A learning healthcare system is one in which the clinical research paradigm depends more judiciously on the serial conduct of randomized controlled trials—important, but often too expensive, untimely, and of limited applicability—and draws more heavily on electronic health records (EHRs) to generate evidence as a natural by-product of the clinical experience.”

More recently, the FDA has launched a Clinical Trials Transformation Initiative intended to promote and enable the conduct of larger, simpler, less expensive randomized trials using streamlining methodologies such as use of electronic health records (70).

In addition to addressing an important clinical question, our proposed trial is responsive to all these needs. This trial represents a new efficient methodology designed to provide reliable answers to practical clinical questions at a greatly reduced cost.

VI. Study Design

The study design is a multicenter clinically integrated (65) [or "point of care" (67,68)] prospective randomized open-label blinded-endpoint (PROBE) trial (71).

VII. Study Population

A. Inclusion Criteria

Eligible patients are those (including women and minorities) who:

1. Are over age 65 years
2. Are receiving hydrochlorothiazide from the VA pharmacy at a daily dose of 25 or 50 mg
3. Have a most recent SBP in EHR ≥ 120 mm Hg, with no SBP < 120 mm Hg recorded in EHR in the previous 90 days

B. Exclusion Criteria

Patients will be excluded if they are known to have any of the following:

1. Impaired decision-making capacity rendering the patient unable to provide informed consent (i.e., if there is any question during the nurse's EHR chart review that the individual does not have the ability to make an autonomous decision or the PCP declines permission to randomize)
2. Death expected within 6 months (inferred by PCP permission to randomize)
3. K< 3.1 meq/L (or 3.5 meq/L if on digoxin) in the past 90 days (assessed by EHR review)
4. Na < 130 meq/L in the past 90 days (assessed by EHR review)
5. Known to be enrolled in Medicare Part C (assessed through administrative data or on consent phone call). This exclusion will only be employed if we determine that we cannot obtain sufficient information from Part C data (see below under Rationale).

Enrollment in another CSP interventional study is not an exclusion criteria for DCP. Dual enrollment is addressed in **Section IX. Study Procedures**.

C. Rationale

We limit to age over 65 years to allow data collection through Medicare (which is only widely available starting at this age) [and will exclude known Part C enrollees (those enrolled in Health Maintenance Organizations), for whom usual encounter data is not available through Medicare if we determine that we cannot obtain sufficient information from Part C data].

We limit randomization to patients receiving hydrochlorothiazide because a) 95% of thiazide-type diuretic prescriptions are for hydrochlorothiazide, b) there is little evidence to suggest that chlorthalidone is inferior to hydrochlorothiazide, and c) the few PCPs who have deliberately chosen to use chlorthalidone over the much more commonly used hydrochlorothiazide are less likely to be willing to change drugs.

We limit to hydrochlorothiazide doses of 25 or 50 mg because lower doses may not be effective and cannot be easily converted to chlorthalidone (which is available only in 25mg tablets), and because higher doses are not recommended and are rarely used.

We limit to SBP ≥ 120 mmHg primarily to minimize risk from hypotension, and also to avoid enrolling patients whose blood pressure is already low enough that a potentially more effective drug would not be expected to add benefit (72, 73). The cutoff point of 120 mmHg was selected, in part, because we are using routine clinic blood pressures recorded in EHR to determine study eligibility. These are obtained using a less rigorous measurement protocol than is normally used in randomized trials and that tends to overestimate blood pressure (74), in one study by a mean of 8 mmHg (75).

VIII. We will attempt to exclude patients in Part C as long as it appears that we will not be able to obtain adequate data. Part C data have not been available for research purposes in the past, but CMS plans to make the Healthcare Effectiveness Data and Information Set (HEDIS) for the years 2006-2011 available sometime in 2015. We plan to check with CMS periodically, and if, before or during enrollment, adequate Part C data appear to be accessible, we will discontinue the exclusion and attempt to enroll these patients. Study Intervention

Participants will be randomly assigned to remain on their current dose of hydrochlorothiazide (25 or 50 mg), or to replace it with half that dose of chlorthalidone (12.5 or 25 mg, respectively), both changeable by the PCP. Chlorthalidone 12.5 mg will require tablet splitting, and a splitter will be mailed with the prescription. [Tablet splitting is a common procedure in VA pharmacy, sometimes including the splitting of hydrochlorothiazide 50 mg to get 25 mg. A recent review (76) concluded that "Tablet splitting does not seem to significantly affect clinical outcomes related to management of hypertension, cholesterol, or psychiatric disorders, nor influence overall patient adherence."]

IX. <u>Rationale for the default use of 1:2 chlorthalidone dose</u>: Because chlorthalidone is not used at lower doses than hydrochlorothiazide in practice (7,28,29), it could be argued that the most appropriate pragmatic comparison would be a 1:1 substitution. We selected the 1:2 dose default for 3 reasons: 1) as noted above, the use of similar doses in practice probably reflects greater awareness of appropriate thiazide dosing by the small proportion of prescribers who use chlorthalidone, rather than belief that the potencies are equivalent, 2) a 1:1 design would change the study question from assessment of a possible inherent difference between the two diuretics to an assessment whether low doses of hydrochlorothiazide are less effective, whereas our interest is in the former question , and 3) an equal dose of chlorthalidone would represent an intensification of diuretic therapy, resulting in increased effects on blood pressure and blood chemistries (51,52,53), whereas the 1:2 design results in virtually no

change in blood pressure or metabolic effects (see Background and Rationale, parts F and I).Study Procedures

A. Procedures prior to patient enrollment

We will attempt to identify a “Liaison” at each site, usually a physician in primary care and/or hypertension management whose role will be limited to identifying key personnel at the site and introducing the study by giving presentations to local Primary Care Providers (PCPS), pharmacy, the Information Resource Management service (IRM), Clinical Application Coordinators (CACs), and Information Security Officers (ISOs) and subsequently referring all questions and comments to Boston CSPCC. The Liaison will not be involved in recruitment or have patient interaction related to the study. Because we are introducing a new design and intend to evaluate its success, PCPs are also considered to be research subjects (see XI. Biostatistical Considerations, F. Secondary Data Analysis, 4. Primary Care Provider Metrics of Interest).

Our procedures are summarized in a flow diagram (**Appendix C**). Working through the VA national ISO structure and local CAC’s and pharmacy personnel, we will obtain permission for Boston CSPCC to have the necessary access to local EHR systems to enter notes and post orders as View Alerts to PCPs and to collect the study data on enrolled patients. Research staff at the Boston CSPCC will be responsible for communicating with local PCPs, coordinating and implementing all patient recruitment activities and completing the enrollment process including randomization and placing assigned treatment orders for signature by the PCP in EHR. The Boston CSPCC will partner with the Minneapolis VA Medical Centers to conduct centralized calling activities, including contacting potential participants and obtaining informed consent by telephone.

We plan to roll out the study in blocks of sites. When a site starts up, the Boston CSPCC will generate a local list of PCPs along with their eligible patients. We will also identify local PCP and pharmacy email groups and obtain the PCPs’ and pharmacists VA Outlook email addresses. The Boston CSPCC will send an introductory letter (by mail and email) to local PCPs signed by the Study Chair providing information about the study (**Appendix A.1, A.7**). Introductory emails (**Appendix A.7)** will be sent by the study team via ProjectFlow, while letters will be sent via external mail contractor. The Site Liaison or study team will present information about the study at primary care staff meetings and other forums (individual PACT meetings, video staff meetings for Community-Based Outpatient Clinics, etc.). If no meetings are available the study may use a video of slides accompanied by an audio track to conduct site education for providers. This video can also be accessed at any time by consented providers that would like to review the study information. To further provider education, a second reminder email with an attached summary document and a link to the video will be sent to providers prior to being contacted within the EHR system (**Appendix A.8**). The second email is to act as a brief reminder of the study to providers, not to act as an informed consent document.

After these activities are completed, a View Alert ‘testpatient’ order will be sent to each PCP identified by the method above. The testpatient order will accompany a progress note containing the text of the Provider Information Sheet, which will contain the elements of informed consent and detail the study

procedures (**Appendix B.3**). By signing the 'testpatient' view alert order, the PCP is agreeing to participate in the study as a research subject and allowing the recruitment letter (**Appendix A.2**) to be sent to eligible patients. Alternatively, the PCP can "discontinue" the order, in which case that PCP will not be enrolled in the study and his/her patients will not be contacted at this time. As some PCPs may discontinue the order because they did not recognize the purpose of the 'testpatient' order we may send a second View Alert 'testpatient' order approximately 8 weeks later to PCPs who decline to give the PCP the opportunity to participate and/or confirm their desire not to participate.

B. Enrollment procedures

The procedures described below are summarized in a flowchart (**Appendix C**). Provider and patient mail will be sent primarily through an external mail contractor and also by the study team, as needed. The external contracted mailing vendor will receive participants' names, addresses, information about treatment assignment, and other protocol procedures. No other patient health information will be sent to the mail vendor.

Using the VA electronic medical record, the Boston CSPCC will identify eligible patients of PCPs who signed the testpatient order. The mailing vendor or study staff will mail the study recruitment letter (**Appendix A.2**) with Informed Consent Information Sheet (**Appendix B.2)**, which contains the text of the consent script (**Appendix B.1**), to these patients. The study team will attempt to send the letter 3-4 months before the patient's next planned appointment with the PCP to make it easier for the PCP to obtain any follow up on blood pressure and laboratory tests that the PCP might want. The VA Advanced Clinical Access/Recall system may prevent early detection of these appointments. The study Invitation Letter and the Informed Consent Information Sheet will provide information about the study and the opportunity for the patient to opt out of future contact from the study. Patients can opt out by leaving a voicemail with their name, date of birth, and the last four digits of their social security number. Patients that leave an opt-out message will only be contacted in the rare case that the their identity cannot be confirmed. Additional Spanish letters will be sent to all patients in Puerto Rico and any patients that request a Spanish version. Any Spanish speaking Veteran will speak to a Spanish speaking study team member, contact a voicemail box recorded in Spanish, and will consent in Spanish.

If no 'opt out' call is received within 2 weeks after the Patient Information Letter is sent, the patient will be contacted to determine if they wish to participate. This will be done in majority by trained callers using a telephone line that displays the calling source as the VA on caller ID. Trained research staff may also consent patients and may also contact patients during follow-up calls. We will make multiple attempts to reach the patient, only leaving a maximum of 3 spaced out messages. More than 3 will only be left if the caller is returning the patient's call. The U.S. National Health Interview Survey used a maximum of 15 call attempts and a 1994 study confirmed this number to be optimal (77). In 2 surveys conducted by Statistics Sweden, nonresponse rates stabilized at 15 and 20 maximum call attempts (78). In the Consumer Assessment of Health Plans Study, "most hard-to reach non-respondents were called 10 or more times" (79). A recent study at the University of Minnesota (80) had an amendment approved by the UMN IRB in 2011 to increase the maximum number of call attempts from 6 to 12. Regarding left

messages, Koepsell (81) “found that leaving a brief message about the study and promising a call-back improved the response rate by nearly 20 percentage points”. If a Veteran expresses interest on the last

call or leaves a voicemail after 15 calls, 3 additional call attempts will be made. On the phone call, the information included in the letter will be reviewed and the patient's informed consent will be sought using a pre-approved Telephone Consent Script (**Appendix B.1**). Research staff and study nurses may occasionally call consented patients to evaluate consent call procedures, discuss any concerns the patient's raise, and to complete withdrawal procedures. Additional study information may be transmitted to Veterans, per their request, in the format that best suits their needs (**Appendix A. 12**). All email communication between the study team and Veterans will occur over My Healthy Vet. The mail vendor or study staff may also re-send introductory study materials to Veterans if they have not been reached after 5 calls. The mail vendor or study staff may also send a newsletters or reminder information to enrolled patients as needed (**Appendix A.15, A.10, A.11**). At the end of recruitment a letter (**Appendix A.20**) will be sent to any patient who has not been given a study status. Meaning they have not declined, agreed to participate, or were otherwise found to be ineligible in the study. This letter will not be sent to patients who have received 6 or more calls, to avoid confusion. Study data will be dispersed to all randomized patients via an end of recruitment letter (**Appendix A. 23**), consented PCPs will receive a the same information via email (**Appendix A.22**)

At the end of recruitment all consented providers will receive an email (**Appendix A.24**) inviting them to participate in a participation survey (**Appendix A.21**). In addition, Minneapolis providers will have the opportunity to take an in-person interview (Appendix A.25). The survey data will be aggregated to inform and improve future designs of pragmatic studies.

After telephone consent is obtained, a preliminary eligibility check will be performed to prevent sending randomization View Alerts to providers for ineligible patients. If a patient is deemed eligible, a View Alert order will be sent to the PCP which, if signed by the PCP, will indicate permission to randomize that particular patient. If a patient is deemed ineligible at that time, a determination will be made whether they are temporarily or permanently ineligible. Temporary ineligibility may be due to lab values or blood pressure measurements out of inclusion range that could change over the subsequent months. Temporarily ineligible patients may receive a letter indicating that they are not presently eligible, but will continue to be considered for randomization if their eligibility criteria change. Eligibility will be re-assessed for temporarily ineligible participants periodically. If a patient becomes eligible again six months or more after consent, a study team member will attempt to call the patient to reaffirm consent. After three attempts the study team will proceed with randomization. Randomization will yield a letter with study information allowing the Veteran to withdraw or contact the study if needed. Permanently ineligible patients will receive a letter indicating that they will no longer be considered for randomization. Alternatively, if the PCP believes that patient should be excluded for any reason (e.g., incompetence or short life expectancy), the order can be "discontinued" and the patient will then receive a letter saying that their PCP declined their participation in the study (**Appendix A.3**).

If an eligible patient is enrolled into another CSP interventional study prior to randomization in DCP, the DCP PI will initiate a conversation regarding dual enrollment with the other study's PI. If the investigators of both studies agree, they will sign a memo describing that agreement and any additional study activities or safety reporting requirements resulting from enrollment in both studies. Additionally, this memo must be signed by the studies' respective CSPCC directors prior to randomization into DCP.

DCP will dual enroll with CSP577: CONFIRM, CSP2005: VALOR, and CSP2016 per the guidance from the signed memos.

C. Randomization

After the PCP signs the order allowing the patient to be randomized, the patient will be randomized by the Boston CSPCC. Randomization will be to chlorthalidone or hydrochlorothiazide with equal probability. Randomized group will be open label, but allocation will be concealed before randomization and irrevocable afterwards. All randomized patients will be included in the analysis according to the intent-to-treat principle. Outcome assessment will be conducted by investigators blinded to treatment assignment.

We are thus proposing a "prospective randomized open-label blinded-endpoint" (PROBE) trial (71). The rationale for our open-label design is several fold: 1) since there is no local coordinator, it is essential that the PCP manage the diuretic therapy, and we are concerned that this may not always occur if we use a blinded study drug, 2) there is no local investigator to assist with emergency unblinding, 3) we believe that keeping patients on open label therapy, being more familiar and straightforward from the patient's perspective, will enhance recruitment, thereby improving both feasibility and generalizability, 4) local pharmacy management of blinded drug would require a level of effort and local engagement in research incompatible with our streamlined study structure, 5) the expense of producing a study preparation that is identical for the two drugs, and then labeling and tracking each patient's therapy, would greatly increase cost, defeating the purpose of our highly efficient clinically integrated design. A recent meta-analysis found PROBE trials to be comparable to blinded trials in terms of assessing antihypertensive drug effect on blood pressure measurements, though clinical outcomes were not examined (71).

At the time of randomization, a templated text order placed by the Boston CSPCC will appear as a View Alert to the PCP. The order will indicate randomized assignment and reference the associated progress note that provides the following reminders: 1) when resolving the view-alert orders, the PCP can accept the order by signing as is or change the dose, 2) the PCP can discontinue the order to continue their patient on his/her current diuretic, 3) the PCP may wish to order any desired laboratory tests or blood pressure checks, and 4) the PCP should manage the diuretic in the future according to the patient's needs.

Patients will be sent a letter informing them of their randomized group (**Appendix A.4**). Patients assigned to hydrochlorothiazide will simply remain on their current prescription. For patients randomized to chlorthalidone, the Boston CSPCC will also generate View Alert orders to the PCP cancelling the hydrochlorothiazide prescription and replacing it with chlorthalidone. Patients randomized to chlorthalidone will be instructed to discard their hydrochlorothiazide pills, and will be reimbursed for their co-pay on the discarded pills. The change from hydrochlorothiazide to chlorthalidone is a typical pharmacy action in usual care and will otherwise be handled with usual pharmacy procedures and information at that medical center.

In the unlikely event that the PCP, having very recently signed the "permission to randomize" view alert order, does not sign (i.e., "discontinues") the initial study drug order for chlorthalidone, the patient will still be analyzed in the randomized group according to intent to treat. In these instances, we may contact the PCP to ask the reason for the discontinuation of the order.

X. We will monitor drug prescribing for study patients throughout the study. If the study drug is discontinued, a prescription is written for the other diuretic (cross-over), or the prescription is not refilled for 90 days after expected, a View Alert may be sent to remind the PCP about the study. If the situation persists after 2 weeks, we will review the chart to determine if the lapse was intentional and if so, try to determine the reason. If questions remain after the review, we may query the PCP.Outcome Measures

A. Primary outcome

The primary outcome measure will be time to a major cardiovascular event, defined as a composite outcome comprised of the first occurrence (after randomization) of any of the following:

1. Stroke
2. Myocardial infarction
3. Urgent coronary revascularization (completed or attempted) because of unstable angina
4. Hospitalization for acute congestive heart failure
5. Non-cancer death

B. Secondary outcomes

Secondary outcomes will include:

1. All deaths
2. The composite outcome substituting all deaths for non-cancer deaths
3. "Possibly vascular deaths" defined as all deaths caused by vascular diseases, diabetes, external causes, and unknown causes
4. The composite outcome substituting "possibly vascular deaths" for non-cancer deaths
5. Each of the 5 components of the composite primary outcome
6. Any revascularization of any artery
7. Erectile dysfunction, defined as first prescription for PDE5 inhibitor or referral for ED

C. Process variables

1. Mean blood pressure during the study (outpatient clinics only; excludes inpatient, Emergency Department and Operating Room)

2. Time to discontinuation of the randomly assigned diuretic (defined as discontinue order or no prescription for ≥ 6 months at last observation during study period)

3. Mean compliance with study drug using "Medication Possession Ratio" (82) (used by VA Pharmacy Benefits Management Services)

4. Other antihypertensive drug use

D. Tertiary Outcomes

1. Hospitalization for primary diagnosis of hypokalemia, hyponatremia, or renal failure

2. Renal failure. Defined as dialysis or renal transplant. (Doubling of serum creatinine from baseline will also be recorded and reported in the final analyses).

3. Other recorded hypokalemia (< 3.1 meq/L), or hyponatremia (< 130 meq/L)

4. New diabetes, defined as first use of a medication for diabetes

5. Acute gout episodes

6. New allergic reaction to thiazide-type diuretic to be found in EMR allergy data

E. Rationale for elements of composite primary outcome

The elements of the composite primary outcome are intended to represent the clinically important effects of diuretic therapy for hypertension. Myocardial infarction and stroke are traditional outcomes for cardiovascular clinical trials and will not be further justified here.

Stroke. Stroke is defined from the American Heart Association/American Stroke Association updated definition of stroke for the 21st century (83) and includes central nervous system infarction (brain, spinal cord, or retinal) attributable to ischemia based on neuropathological, imaging, and/or clinical evidence; ischemic stroke accompanied by overt symptoms; and stroke caused by intracerebral hemorrhage, subarachnoid hemorrhage, or cerebral venous thrombosis. Stroke also includes any episode of acute neurological dysfunction presumed to be caused by ischemia or hemorrhage, persisting ≥24 hours or until death, but without sufficient evidence to be classified as one of the above. We exclude silent central nervous system (CNS) infarction, silent cerebral hemorrhage, major brain trauma including subdural hematoma or hemorrhaging, intracranial neoplasm or metastasis, coma due to metabolic disorders or disorders of fluid or electrolyte balance, peripheral neuropathy, or central nervous system infections.

Myocardial infarction. Myocardial infarction is defined from the third universal definition as evidence of myocardial necrosis in a clinical setting consistent with myocardial ischemia (84).

Urgent coronary revascularization. Urgent coronary revascularization is an important outcome because these events accurately reflect both the progression of underlying atherosclerotic vascular disease and represent an important contributor to cardiovascular morbidity and healthcare costs. Disease progression that formerly resulted in myocardial infarction now frequently results in revascularization that aborts the infarction. Furthermore, because of increasingly sensitive troponin assays, many patients having urgent coronary revascularization attributed to unstable angina actually have mild troponin positivity and would be classified as myocardial infarctions in a classic trial design with in-depth outcome review (Christopher P. Cannon, MD, personal communication, Aug 2013). Including urgent coronary revascularization in our study will prevent us from missing these infarctions. Urgent coronary revascularization is more restrictive than the revascularization outcome usually reported in previous trials and selects for the most clinically relevant events. For example, in the PEACE trial of patients with stable coronary artery disease and normal ejection fraction (85), “all coronary revascularizations” were more frequent than all the other events combined that make up our composite. On the other hand, in the (TRA2°P)-TIMI 50 trial of an antiplatelet agent in patients with vascular disease (86), urgent coronary revascularization because of unstable angina was less frequent than myocardial infarction and similar in frequency to cardiovascular death or stroke, and increased the composite rate in the placebo group from 10.5% for those 3 outcomes to 12.4% for those 3 outcomes plus urgent revascularization.

Reductions in urgent coronary revascularization represent an important contribution to the overall effectiveness of therapy. The observed reductions in urgent revascularizations in randomized trials are concordant with changes in other major outcomes. In (TRA2°P)-TIMI 50 (86), the hazard ratio for the primary composite outcome of cardiovascular death, myocardial infarction, or stroke was 0.87, and for urgent coronary revascularization was 0.88. Urgent coronary revascularization has also been used in TRACER (87) and is being used in EXAMINE (88). CSSEC recently approved a broader revascularization outcome for VA CSP #593, the VA Fenofibrate Intervention Trial (VA-FIT) (for which the indications included stable angina with a >50% target lesion).

In summary, urgent coronary revascularization is a significant contributor to cardiovascular morbidity and health care costs, its inclusion will allow assessment of the full impact of therapy, and its relative contribution to overall endpoints is not anticipated to be disproportionate to that of other study outcomes. Our definition of urgent coronary revascularization will consider completed or attempted coronary revascularization procedures performed because of unstable angina. Unstable angina is further defined as any increase in angina, and/or inadequate response to increased anti-anginal therapy cited as the reason(s) for the procedure, and with the medical record citing that increased angina and/or inadequate response to increased anti-anginal therapy occurred within 30 days before the inpatient procedure code for coronary revascularization.

Hospitalization for acute congestive heart failure. Hospitalization for acute congestive heart failure is another important component of our primary outcome. It will, in most instances, represent a new diagnosis of heart failure because most patients with established heart failure are maintained on a loop diuretic such as furosemide and are not treated with hydrochlorothiazide and thus would not be enrolled in our study. We will seek to identify clinical exacerbations of symptoms (e.g., not

hospitalization for ICD placement), which should be associated with intensification of treatment, which we will assess in our algorithm.

Heart failure is a major public health problem with a profound impact on prognosis and also on costs, and is the most frequent cause of hospitalization among people older than 65 years (89). Its impact is summarized in the AHA 2013 update (90): “HF incidence approaches 10 per 1000 population after 65 years of age”, …“approximately 50% of people diagnosed with HF will die within 5 years”, …“One in 9 deaths has HF mentioned on the death certificate”, …“In 2009, HF any-mention mortality was 274 601” and “HF was the underlying cause in 56 410 of those deaths”.

Heart failure and stroke are the two major cardiovascular outcomes most related to hypertension and most benefited by treatment of hypertension. Seventy-five percent of heart failure cases have antecedent hypertension (90). The Framingham Heart Study (91) found the highest risk ratio from hypertension to be for heart failure in men (Figure 1).

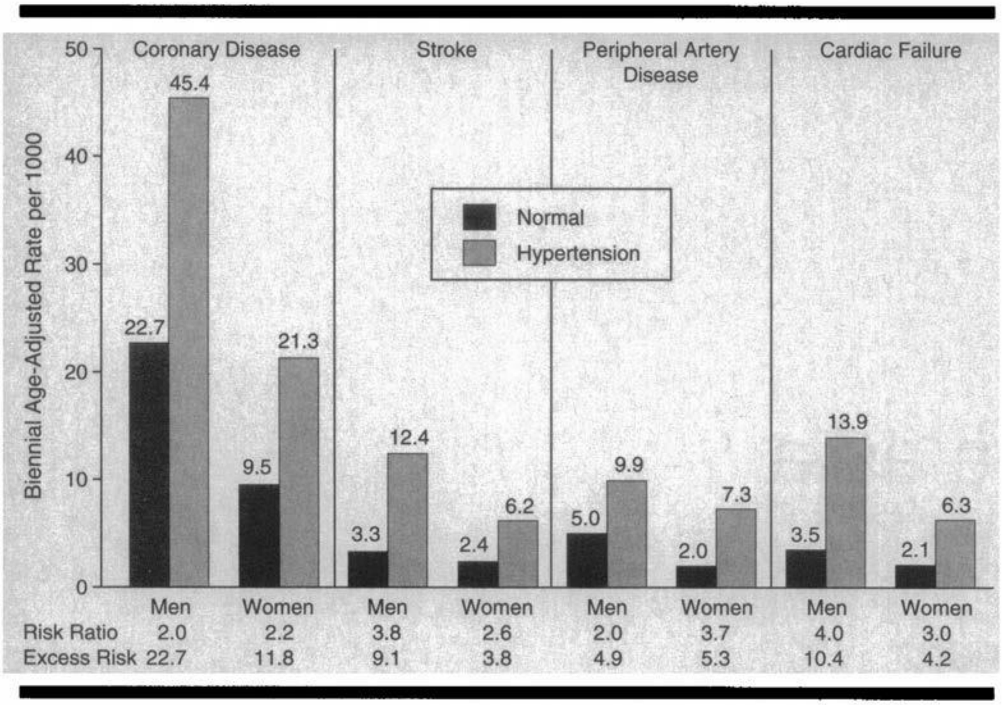

Figure 1. A prospective analysis of the 36-year follow-up data from the Framingham Heart Study (91) demonstrates that hypertension (blood pressure ≥ 140/90 mmHg) predisposes to all major atherosclerotic cardiovascular disease outcomes, but the largest risk ratios are for cardiac failure and stroke in men.

Diuretic-based treatment arms in outcome trials have reduced HF by an average of 50% over placebo (compared with 30-40% reduction for stroke), and more effectively than calcium channel blockers and ACE inhibitors in comparative trials (Figure 2) (5). In ALLHAT (89), the alpha blocker doxazosin, the calcium channel blocker amlodipine, and the ACE inhibitor lisinopril were associated with 80%, 38% and 19% higher risk of heart failure compared with chlorthalidone. Therefore, heart failure is likely to be one

of the most sensitive outcomes for detecting a true difference in reducing cardiovascular disease between the two diuretics in our study.

Figure 2. Network Meta-analysis of First-Line Treatment Strategies in Randomized Controlled Clinical Trials in Hypertension

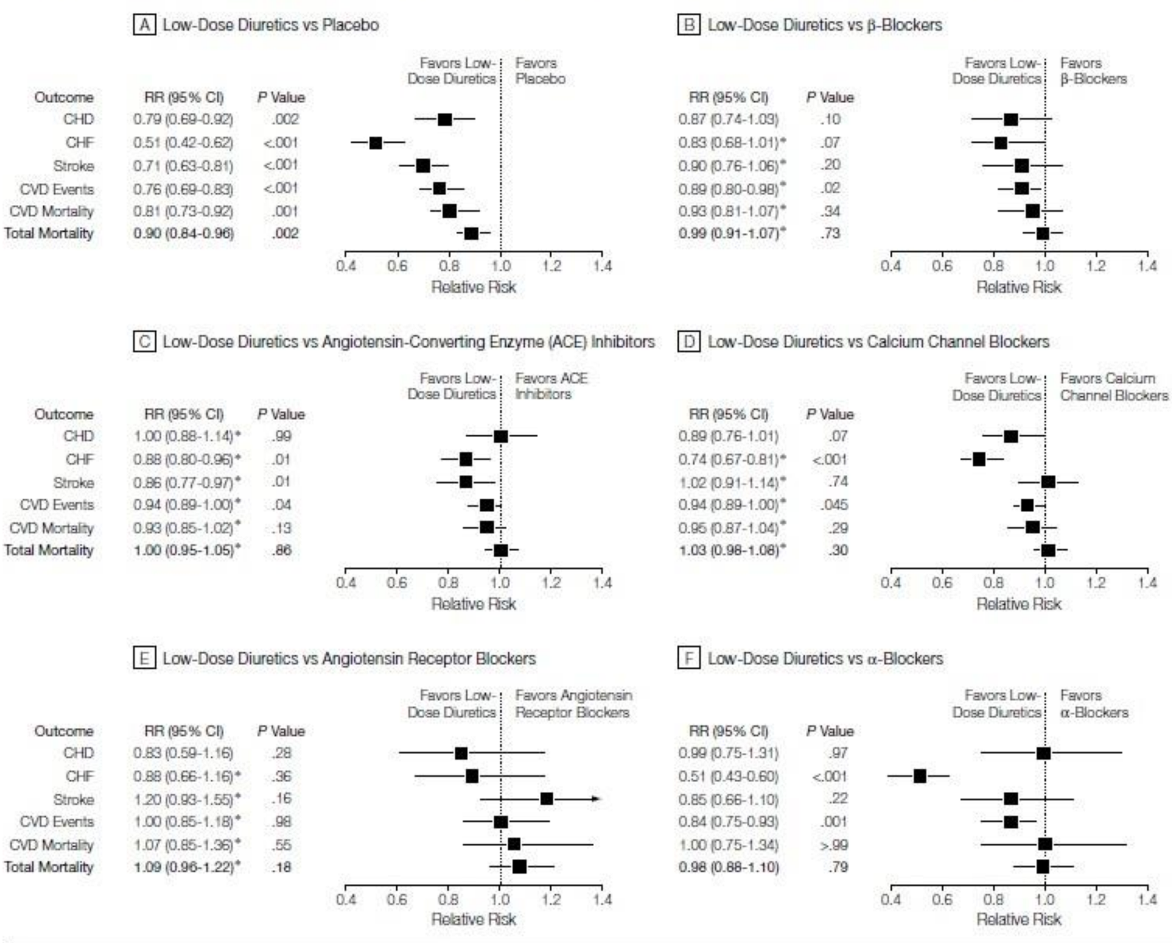

Figure 2. From a network meta-analysis of hypertension trials (5). Heart failure (CHF) can be seen to be a particularly effective outcome for discriminating between diuretics and other drugs. “CVD Events” include CHD, CHF, stroke and CVD deaths.

Hospitalized heart failure is not disproportionately common compared with our other outcomes. In the principal ALLHAT report (20), there were 885 non-fatal MIs, 724 hospitalized or fatal heart failure events, and 675 strokes. Hospitalized heart failure events in ALLHAT attracted close scrutiny because they were reported by site investigators and not adjudicated. The authors undertook a separate study in which they successfully validated these events (89), and from which they concluded “Heart failure proved to be a common outcome in ALLHAT and one that was affected differentially by the randomized treatment assignments. In addition, patients who developed HF had significantly poorer survival than those who did not. Whether this poor prognosis can be altered presents an important clinical and public health question. Thus, *in planning future hypertension treatment trials* (and perhaps also treatment trials in other populations at high risk of HF, such as diabetics and survivors of acute coronary

syndromes) *serious consideration should be given to including HF in the primary end point*, along with death, MI, and stroke" (italics added).

Heart failure is reasonably well identified using discharge diagnoses. In a population-based study of 4537 cases of heart failure from Olmsted County MN, ICD-9 code 428 constituted 80% of heart failure codes, and 82% of the cases coded as 428 met Framingham criteria for heart failure when records were reviewed by experienced abstractors (92).

Identification of clinically important episodes can be improved by looking for evidence of treatment intensification. The draft report of the FDA expert panel on Cardiovascular Endpoints in Clinical Trials (93) recommends inclusion of the following criteria for determining a heart failure hospitalization event: "The patient receives initiation or intensification of treatment specifically for HF, including at least one of the following: a. augmentation in oral diuretic therapy, b. intravenous diuretic, inotrope, or vasodilator therapy, or c. Mechanical or surgical intervention, such as i. mechanical circulatory support (e.g., intra-aortic balloon pump, ventricular assist device) or ii. mechanical fluid removal (e.g., ultrafiltration, hemofiltration, dialysis)." We will employ these criteria and others, such as exclusion of admissions for ICD placement, in our algorithms to maximize the likelihood that the events we capture are clinically relevant.

<u>Non-cancer deaths</u>. We include non-cancer deaths in our primary composite outcome as a compromise between total mortality, which includes many irrelevant events that dilute the effect of the intervention, and cardiovascular mortality, which is less accurately distinguished on death certificates and may miss relevant deaths. We exclude cancer deaths because they are numerous, relatively accurately identified on death certificates (94,95,96), and believed to be unrelated to diuretic use, so excluding them reduces "noise" relative to expected effects of the intervention. In the Physicians' Health Study (95), cancer deaths in patients older than 65 years were identified from death certificates using standard nosology protocols and the Automated Classification of Medical Entities (ACME) Decision Tables with specificity well over 99% compared with an adjudication committee, meaning that the deaths excluded from our primary outcome will almost certainly be due to cancer.

Prieto-Merino and colleagues (97) note: "If outcomes that are causally related to the trial treatment are combined with those that are not, the estimate of the treatment effect is diluted towards the null and we may fail to identify potentially important benefits or harms…. Because few treatments will be causally related to all causes of death, all-cause mortality is a composite outcome that combines causally related causes of death with those unrelated to the treatment." We did not restrict to cardiovascular deaths because some other deaths may be relevant, such as accidents due to syncope from hypotension and deaths from unknown causes (which could have been cardiovascular), and other deaths (e.g., pneumonia, COPD) that are not accurately distinguished from cardiovascular deaths by death certificate diagnoses. Competing risk from cancer deaths will be considered in a secondary analysis.

F. Ascertainment of Outcome Data

Data collection will be via electronic medical records, administrative data, Medicare and death records. We will use VA, CMS, and National Death Index data. Assessment of outcomes and relevant data

VA Central IRB

elements as well as adverse event is by passive collection of data in electronic health records; the study will not attempt to generate any additional tests or procedures. All outcome processing will be conducted by investigators at the Boston CSPCC unaware of treatment group. In this section we describe our approach to ascertainment and assessment of these outcomes.

The Boston CSP Coordinating Center has 15 years of experience in ascertainment and assessment of cardiovascular outcomes using the VA electronic health record (EHR) and CMS data. We will build on this extensive experience to refine a specific procedure for doing the same in this trial in order to accurately identify and assess outcomes. The process involves several clearly defined steps. First, we develop a method to screen the electronic medical records of all participants on a periodic basis for potential cases. This screening consists of comparing discharge diagnosis and procedure codes for enrolled patients to a list of outcome-relevant ICD 10 and CPT codes. Admissions that do not match codes on our list will be considered non-events. The development of the screening process and the algorithms for confirming cases follows a systematic approach that we have used at the Boston CSPCC for a number of years. The screening approach is used to identify all potential cases using key elements in the EHR. The screening tool varies for each outcome (i.e. outcome-specific administrative diagnosis codes).

Second, algorithms are developed to collect and analyze data elements that are used to confirm or refute the potential cases (e.g. presence of imaging during a stroke). The algorithms are based on elements of the clinical definitions of the outcomes, and are applied to potential cases to determine if the case is confirmed, disconfirmed or deemed indeterminate. Algorithms for cases are constructed using “gold standard” cases (identified by manual chart review) and data elements obtained from the medical record that are found in the outcome definitions, such as cardiac enzymes for MI. Once the algorithm for a specific case is defined, the algorithm’s accuracy is checked by manual review of cases identified by the algorithm.

Third, indeterminate cases will undergo manual adjudication. We expect to be left with a relatively small number of events that cannot be resolved by the algorithm, but all of these will be referred for adjudication to an outcomes committee. This will greatly lessen the workload for manual adjudication. All patients will be followed until death or the end of the study (even if the primary outcome is determined to have occurred) to collect secondary outcomes, including death.

A pilot study using VA EHR was conducted to determine the availability of potential cases and core data elements that would be needed to develop algorithms to assess the primary outcomes. A total of 150 medical records were reviewed, 30 in each category for stroke, myocardial infarction and urgent coronary revascularization and 60 records of patients with a diagnosis of acute congestive heart failure. A medical record abstraction form for each outcome was developed based on standard diagnostic definitions that included information on symptom presentation, physical findings, critical laboratory values, radiographic or imaging findings, electrocardiographic results, hemodynamic data and administration of medications and therapeutic interventions (84, 98) During this study these data will be augmented with CMS data for all participants. Below we describe the approach to ascertainment of

VA Central IRB

potential cases. Definition criteria and ICD diagnostic and procedure codes for each outcome can be found in Appendix F (Outcome Definitions)

G. Adjudication of events

In cases where the outcome diagnosis is not clear based on the screening mechanism and algorithms developed, we will resort to manual adjudication to determine the validity of the diagnosis. Based on our 150 chart review, it is expected that fewer than 300 to 400 cases will require this form of adjudication. The adjudication will consist of a chart review by qualified clinicians of medical records pertaining to the hospital admission for VA admissions and a chart review of VA inpatient and outpatient records as well as CMS data following the discharge date for non-VA hospitalizations. In some cases, we may query the PCP as to whether a possible event occurred.

Additionally, we will also manually adjudicate 10% of all outcomes confirmed or refuted by our algorithms to serve as a quality check on the algorithms. We will develop near-final algorithms during the study and will continue to refine the algorithms and retrospectively apply these refinements to all data. It is also important to note that between the time of study approval and study launch, the VA has transitioned to using the ICD-10 classification system. We will be mapping the ICD-9 codes described in this protocol to the equivalent ICD-10 codes as we continue to develop our algorithms.

H. Ascertainment of potential cases, adverse events and other case data

XI. Investigators at the Boston CSPCC unaware of treatment group will process potential cases and adverse events by collecting relevant codes, laboratory values, and prescription data from EHR, the VA electronic medical record system, and Medicare (e.g., acute gout episode = ICD10 M10.00). Blood pressures will be collected from routine clinic visits, and not from emergency room visits or inpatient stays, and only the reading with the lowest SBP of the day will be retained (99). Section XIII details the data sources and the data collection (i.e., data extraction) procedures that will be used. When the study drug is discontinued, we may query the PCP as to the reason if not clear from the charBiostatistical Considerations

A. Overview of the Study Design

The proposed study is a prospective randomized open-label blinded-endpoint, multicenter, two arm intervention trial testing the effectiveness of chlorthalidone for prevention of cardiovascular events and non-cancer death among patients currently receiving hydrochlorothiazide. The primary hypothesis is that chlorthalidone is superior to hydrochlorothiazide for the prevention of cardiovascular events and non-cancer death over time.

The primary outcome measure is time from enrollment in the study to the first occurrence of a cardiovascular event or non-cancer death. Cardiovascular (CVD) events are defined as stroke, myocardial infarction, urgent coronary revascularization, and hospitalization for acute congestive heart failure. The results for CVD and non-cancer death event-free survival will be analyzed by means of a two-sided log-rank test.

VA Central IRB

The study will have one interim analysis and one final analysis.

1. **Estimated Incidence of the Primary Endpoint**

This study will randomize up to 13,700 patients, in 50:50 allocation to hydrochlorothiazide and chlorthalidone, in order to achieve 1,055 primary outcome events. . All subjects will be followed through the end of the four and one-half (4.5) year study period yielding an estimated average follow-up time of three (3) years. We posit a four and one-half year rate of 13.5% of the composite outcome in the hydrochlorothiazide group and 11.1% in the chlorthalidone group. We utilized the VA National data from fiscal years 2010 to 2012 to identify a subgroup of subjects who would be potentially eligible for the proposed study. Details of the analysis can be found in **Section XII. Feasibility and Recruitment Plan**.

The cardiovascular event rate, using a composite similar to that proposed here, was 2% per year in ALLHAT (20) and ACCORD (101), and is projected as 2% for the ongoing SPRINT. Unlike those studies, we are limiting enrollment to patients over age 65 years and are not excluding very old or seriously ill patients (unless life expectancy is known to be less than 6 months), and we are including all non-cancer deaths rather than only cardiovascular deaths, all of which would be expected to increase the event rate. For patients 65-79 years old with no serious illness (and creatinine <1.5 mg/dL) in ACCORD, the composite event rate (stroke, myocardial infarction, and cardiovascular death) was 2.8% per year (96). Our event rate should be higher because we include urgent revascularization, acute heart failure, and non-cancer deaths. ANBP2 enrolled patients over 65 years but included all deaths in the composite, and observed an event rate of >4% per year (19).

We believe that the most relevant event rates for our study come from the Ontario observational study (29) comparing chlorthalidone with hydrochlorothiazide, discussed above. The Ontario study is both recent, published in 2013 and reporting data from 1993 to 2010, large (nearly 30,000 patients), and shares important features with our proposed trial, including: 1) study patients are aged 66 and older and taking diuretics for hypertension, 2) outcome data were collected passively from administrative databases, and 3) the primary outcome was a composite similar to the one we are proposing, with two differences: a) they did not include urgent revascularization, and b) they included all deaths whereas we do not include cancer deaths.

We expect these 2 differences to very nearly cancel each other’s effect. The rate for the Ontario composite outcome was 3.4% per year in the hydrochlorothiazide group. The rate for all deaths was 1.8% per year, or 45% of the total number of individual outcomes (some patients had more than one of the composite outcome elements). Many studies, such as the Physicians Health Study (95) have found that all deaths in this age group are comprised of roughly 1/3 each of deaths due to cardiovascular disease, cancer and other causes, suggesting that our exclusion of cancer deaths would reduce the composite rate by 15%. In the (TRA2°P)-TIMI 50 trial (86), urgent coronary revascularization was less frequent than myocardial infarction (0.82% in the Ontario study, or 20% of the total number of individual outcomes) and similar in frequency to stroke (0.46% in the Ontario study, or 11% of the total

VA Central IRB

number of individual outcomes), suggesting that our inclusion of this outcome will raise the composite by 10-15%.

These adjustments result in a best estimate for the expected composite rate of about 3.2% per year. For the proposed study, because event rates tend to decrease over time, we conservatively project a 3% per year event rate in the hydrochlorothiazide group.

2. **Effects of the Intervention**

Of 125,000 VA patients started on drug in routine practice in 2004, 72% of hydrochlorothiazide users and 62% of chlorthalidone users remained on the drug 1 year later (28). Onsite coordinators in standard trials may be able to maintain better drug adherence than occurs in usual practice. ACCORD (101) and SPRINT (84) considered a 20% relative reduction to be an appropriate minimum important difference for power calculations. Because the difference in outcomes in our study could be reduced by patients coming off drug more often than in previously published trials, we reduce the minimum important difference value to **17.5%**. A reduction in CVD events or non-cancer death of this magnitude or greater would be considered clinically significant.

B. The Primary Analysis

We posit a four and one-half event rate of 13.5% in the hydrochlorothiazide group and a 17.5% reduction of CVD events in the chlorthalidone group to inform our primary hypothesis.

Formal statement of the primary hypothesis

Under the null hypothesis:

The 4.5 year event rate will be 13.5% (or 578 primary events)

Under the alternative hypothesis for study participants treated with chlorthalidone:

The 4.5 year event rate will be 11.1% (or 477 primary events)

The reductions attributed to chlorthalidone may be viewed in several ways. The absolute reduction from 13.5% to 11.1% is 2.4%, the relative reduction is (13.5 – 11.1)/13.5 = 17.5%, and the hazard ration (chlorthalidone hazard rate/hydrochlorothiazide hazard rate) of 0.81 is approximately midway between the simple odds ratio, 11.1(100-13.5)/13.5(100-11.1) = 0.80 and the risk ratio 11.1/13.5 = 0.82.

Formally, the null hypothesis is that the two treatment groups do not differ in their time-to-event hazard rates. The alternative hypothesis is that chlorthalidone has a lower or higher hazard rate than hydrochlorothiazide therapy with a hazard ratio for chlorthalidone compared to hydrochlorothiazide less than 0.82 or greater than 1.22. We will test this hypothesis with a two-sided log-rank test.

C. Sample Size and Statistical Power Considerations for the Primary Hypothesis

The formal hypothesis test is two-sided allowing for chlorthalidone to be either more or less effective than hydrochlorothiazide. A significant difference showing that the intervention chlorthalidone compared to the control hydrochlorothiazide decreases (or increases) the hazard of a major cardiovascular event will be regarded a positive result. The results for the primary outcome measure will

VA Central IRB

be analyzed by means of the two-sided log-rank test to detect either a hazard ratio that exceeds 1.22 or is less than 0.82. The test will have a two-sided 4.9% type I error. The test has 90% power to detect a hazard ratio of 1.22 or larger or 0.82 or less with a total of 1,055 primary outcome events. The remaining Type I error of 0.1% is used for the interim analysis.

If the annual event rate is 2.5% (rather than the posited 3%), then the study has 84% power; if 2%, it has 75% power.

D. Primary Data Analysis

The primary outcome hypothesis will be a time-to-event analysis with the use of a two-sided log-rank test based on intention-to-treat principles. The model will not include any covariates. Analytic reports will include hazard rates, their ratio, and the 95% confidence interval about the ratio.

This will be followed by further refined covariate-adjusted exploratory analyses, using Cox proportional hazards regression modeling, controlling for baseline factors. Covariates will include demographic factors (e.g., age, sex, smoking status, education) and clinical factors (e.g., blood pressure, medications, comorbidities, history of disease, and BMI). We will test the proportional hazards assumption by including a time-treatment interaction term in the model.

E. Interim Analysis

We will perform one interim analysis when the 500th event occurs, approximately 3.5 years after initiation of enrollment. Assuming a uniform rate of enrollment over time, the first patient entered will potentially have 3.5 years of follow-up and the last patient entered will potentially have 0.5 years of follow-up. Thus, we will have an average follow-up time of approximately 2 years on 13,500 subjects.

Using the O-Brien Fleming procedure, this interim analysis will have a type I error of 0.1%, which negligibly decreases the overall type I error and has virtually no effect on the power to show that chlorthalidone is different from hydrochlorothiazide. The sample size of 13,532 accounts for the interim analysis with a corresponding inflation factor, for increase in sample size of 1.001, resulting in14 more subjects per arm (102). We will confer with the Data Monitoring Committee (DMC) members and the program leadership for potential stopping guidelines based on findings from the interim analysis.

F. Secondary Data Analysis

1. Treatment Effect in Subgroups
   The Cox regression modeling will explore the possibilities of treatment variation across pre-specified subgroups based on status at time of enrollment including:
   a) gender,
   b) age (dichotomized at median),
   c) baseline SBP (dichotomized at median),
   d) history of MI or stroke,
   e) black race vs. not,
   f) diabetes vs. not,

VA Central IRB

g) eGFR < 60, and
h) good compliance (medication possession ratio ≥ 80%) with hydrochlorothiazide over the year before randomization.

2. Individual Components of Primary Outcome
In addition to the subgroup analyses listed above, each component of the composite primary outcome measure (stroke, myocardial infarction, urgent coronary revascularization, hospitalization for acute congestive heart failure, and non-cancer death) will be separately analyzed using log-rank and Cox proportional hazards models to compare chlorthalidone and hydrochlorothiazide. The Cox analyses will include the covariates considered in the primary data analysis.

3. Additional Outcomes of Interest (Analysis of Secondary and Tertiary Objectives)
In addition we will run time-to-event analyses comparing the treatment effect on:
a) all-cause mortality,
b) the composite outcome substituting all deaths for non-cancer deaths,
c) vascular deaths defined as all deaths caused by diabetes, vascular diseases, external causes, and unknown causes,
d) the composite outcome substituting vascular deaths for non-cancer deaths,
e) any revascularization of an artery,
f) hospitalization for primary diagnosis of hypokalemia, hyponatremia, or renal failure,
g) other hypokalemia (< 3.5 meq/L), hyponatremia (< 130 meq/L), or renal failure,
h) new diabetes, requiring medications, defined as first use of medication for diabetes,
i) acute gout episode,
j) erectile dysfunction (ED), defined as first prescription for PDE5 inhibitor or referral for ED, and
k) new allergic reaction to thiazide-like diuretic, defined as new flag warning in EHR

This may include characterizing healthcare utilization associated with these outcomes, evaluation of incidence of the conditions, and defining these conditions with algorithms using administrative healthcare data.

4. Primary Care Provider Metrics of Interest.
   a) percent of patients approved
   b) percent of approved patients for whom order signed
   c) reasons order not signed
   d) rate of discontinuation of both drugs
   e) reasons for discontinuation of both drugs

5. Medication Compliance
We will compare treatments with respect to overall compliance (adherence) to the randomly assigned medication, indirectly measured by the medication possession ratio (MPR) and average daily dose (ADD). The comparison will be made using a GEE analysis to account for varying

VA Central IRB

periods of follow-up. The potential period ends if the subject dies, has an outcome event, or changes medication. Subjects categorized as medication compliant will make up the per-protocol subgroup of the study cohort.

6. Non-acute Outcomes
The repeated measures of systolic blood pressure during the disease-free intervals (free of component events) will be compared by treatment. We will use a mixed effects repeated measures model allowing for irregular time intervals, the spatial power law extension of AR(1) covariance option where r(t1,t2) = $r(t_1, t_2) = \exp(|t_1 - t_2|)$. In each model we will assume linear growth over time. A random effect will be included for both intercept and linear time. The hypotheses will test if SBP increases/decreases over time for subjects receiving chlorthalidone, if SBP increases/decreases over time for subjects receiving hydrochlorothiazide, and if both have non-zero slopes, a test of whether they differ or are equal.

We will conduct a time-to-event analysis comparing treatments for the outcome of time to first discontinuation of assigned diuretic (defined as no prescription for ≥ 3 months at last observation during study period) and time to first protocol deviation from assigned diuretic.

G. Exploratory Objectives

1. Per-protocol analysis
The primary intent to treat analyses of randomly assigned treatments takes no account of post-baseline changes such as protocol deviations and treatment switches from chlorthalidone to hydrochlorothiazide (and vice versa). Thus, exploratory analyses will model such changes to assess robustness of conclusions.

The per-protocol analysis will determine if chlorthalidone and hydrochlorothiazide differ among the subset of protocol compliant subjects. We will run other analyses that include all subjects and attempt to model the time-dependent effects of protocol deviations such as medication changes and levels of compliance. Frailty analyses will assess center effects. We will explore censoring patterns such as models that assume not missing-at-random censoring (103).

A change in medication may alter the subsequent risk of a cardiovascular event. First, we will add time-dependent covariates to a Cox model, such as binary indicators of a switch from chlorthalidone to hydrochlorothiazide, switch from hydrochlorothiazide to chlorthalidone, and the start of new medication that interacts with chlorthalidone or hydrochlorothiazide. To directly estimate subsequent risk, we will use a multistage model (MSM) to assess the hazard rate of a major cardiovascular event after a switch (104). MSM models extend the time-to-first-event models to second, third, and more events. This extension of the Cox model allows direct estimation of the hazard rate associated with any transition adjusted for previous history and baseline characteristics. Within MSM we will carry out a competing risks analysis to assess the effect of cancer deaths.

2. COVID-19

VA Central IRB

COVID-19 is understood to more severely impact older patients with pre-existing conditions. There is also evidence from the Centers for Disease Control and Prevention that many patients with major medical events are not seeking the care they require during the COVID-19 pandemic.

The DCP patient population is 65 or older with known baseline cardiovascular diseae, and many overweight with diabetes. With a primary outcome consisting of components that often require immediate medical attention, there is a possibility that the observed rate of the study's primary outcome will be impacted by the pandemic. We will characterize event rates throughout the study to assess the impact of COVID-19. We will also assess primary outcome event rates in COVID-positive patients and in geographic locations with particularly high rates of infection.

H. Randomization

To control for potential imbalance in randomization, both stratification and blocking will be employed. The randomization scheme will be stratified by participating site to account for possible regional differences in clinical practice. Participants will be randomized to either hydrochlorothiazide or chlorthalidone within blocks of size 6.

Enrollment and randomization of subjects will occur in accordance with **Section IX. Study Procedures** and **Appendix C. Recruitment Flowchart**.

XII. Feasibility and Recruitment Plan

A. Feasibility

In 2012, 69 VA medical centers prescribed single-agent hydrochlorothiazide 25 mg or 50 mg to at least 2000 patients over age 65 (**Appendix E.1**). In VISN1, 80% of these had SBP ≥120 mm Hg at last measurement with no SBP measurement below 120 mm Hg in the previous 90 days (**Appendix E.2**). Applying these regional SBP data to the national medical center data, in 2012 there would have been 76 VA medical centers with ≥1500 eligible patients and 104 centers with ≥1000 eligible patients.

Based on experience with the CONFIRM study (CSP #577) and the Million Veteran Program and other sources, we estimate that: 15% of PCPs will opt out; PCPs who don't opt out will exclude 5% of their patients; 5% of patients mailed the initial letter will opt out; we will reach 65% of patients we attempt to call; 60% of those we reach by phone will agree to be randomized; 2% of those who agree by phone will opt out or be removed by their PCP before randomization. Combining these rates: (.85)(.95)(.95)(.65)(.6)(.98) = .29, suggesting we should expect to enroll about 30% of eligible patients identified.

Based on these considerations, randomization of 13,700 patients (270 per site at 50 sites) should be feasible. We anticipate that this process will take 3 years, a duration that could be adjusted by the level of staffing of the central call center. If recruitment falls short of our expectations, our primary strategy will be to add additional sites as needed.

VA Central IRB

B. Recruitment Plans

As previously described the primary recruitment plan will be to enroll patients who have been identified through the VA Corporate Data Warehouse as being eligible and cleared by their PCP through the direct mailing of an information letter followed by telephone based consent. Enrollment will be initiated in 3 VISNs at the start of the study. This will allow the DCP team to learn about the feasibility of recruitment, to identify issues with provider participation and EHR use, and to refine the primary recruitment plans based on the experience from the vanguard sites. Patients will also be allowed to self-refer to the study call center as well. In this regard, the DCP will allow all patients fitting the eligibility criteria to be enrolled regardless of how they were identified.

XIII. In addition, we will initiate 20% more sites (i.e., 10 additional sites) than what is needed for successful enrollment into the study. As sites fail at recruitment or as the potential patient pool decreases at the vanguard sites, enrollment into the DCP will be started at the 10 additional sites. Data Collection and Data Sources

Data for this study will be obtained from the medical and administrative data that are collected and maintained by the United States Department of Veterans Affairs (VA) Corporate Data Warehouse (CDW). This database covers the entire veteran population that utilizes the VA and contains individual information on demographic factors, medical history, key laboratory values, procedure codes, and diagnoses (inpatient and outpatient) coded with the ICD-9-CM and ICD-10 classification systems. Healthcare encounters outside of the VA system will be captured using Medicare data that will be obtained from VIReC for this project. Data from the various VA databases (including COVID-19 data from the COVID-19 Shared Data Resource) will be linked together using a unique veteran identification number that is assigned to each veteran at entry into the system.

Vital status will be ascertained from the VA Vital Status File. This file allows for complete ascertainment of death as it pulls data from multiple sources, including: the Beneficiary Identification and Records Locator Subsystem database; the Death Master File from the Social Security Administration; and the National Patient Care Database. Ascertainment of death with this method has demonstrated 98% sensitivity and 98% agreement with the National Death Index. Cause of death will be collected from the National Death Index, as the VA Vital Status File does not aggregate cause of death across the aforementioned data sources.

We will monitor for discontinuation or expiration of the assigned diuretic, detection of which will prompt queries to the PCP asking the reason. No effort will be made by the study to influence blood pressure goals or the prescribing of other drugs.

XIV. Data Management and Data Security Plans

The Boston CSPCC will create and maintain an electronic study database to manage the trial data. All study data will be collected electronically from EHR and a study-specific web application by the Coordinating Center throughout the duration of the study. There will be limited paper-based study documents.

VA Central IRB

Study data is housed on secure VA servers, encrypted and protected in accordance with VA policies compliant with FDA requirements, Federal Information Security Management Act and the HIPAA Privacy and Security rules. Data for this study will be stored in two locations: 1) on a VINCI-hosted server and 2) on a Boston CSPCC-hosted server. Boston CSPCC personnel manage the data access request process for the electronic systems to ensure that data access is appropriate for each individual and the level of the individual access. Study project management will manage the Data Access Request Tracker (DART) activities associated with granting VINCI access to study databases. VA's Office of Information & Technology (OI&T) is responsible for managing other VA system access and ensuring the security and integrity of VA information systems, including the databases and servers housing study data. In accordance with VA Handbooks and Directives, OI&T is responsible for ensuring that appropriate firewalls and data security is implemented and maintained, that data backups are performed and that data may be restored in the event of a system malfunction.

Data security incidents will be reported according to 1058.01. All Boston CSPCC staff will be expected to report data security incidents to the responsible authority as they become aware of the breach. Whenever possible, the reporting of data security incidents will be handled by the Boston CSPCC Associate Center Director for Quality Assurance (ACDQA). This will be done to facilitate communication between the center and the oversight bodies. In the event that an incident must be reported by a staff member other than the ACDQA, all communication after the initial report will be handled by the Boston CSPCC Center Director, the Boston CSPCC ACDPOC, or the Boston CSPCC ACDQA. [Note: all new and current Boston CSPCC personnel will be trained on reporting data security incidents.]

All local data security incidents will be reported in accordance with VA policy within one hour of discovering the incident to:

1. The Boston Information Security Officer (ISO)
2. The Boston Privacy Officer (PO)
3. The Boston ACOS for Research
4. The Boston CSPCC Quality Assurance department

Data security incidents will be treated as unanticipated problems by the Boston CSPCC and reported to the VA Central IRB according to the procedure detailed below for unanticipated problems (Section XVII.C.3).

Study data will be coded and stored using a unique study identifier for each participant. Identifiable information will be collected for patient tracking and safety purposes, and kept in an encrypted, password protected file to which a small number of people will have access. Access to the cross-walk file linking the participant's identifiers and their study data will be restricted to the approved personnel at the CSP coordinating center. At the end of the study, study data will be stored according to CSP guidelines and procedures. Retention of data will be conducted according to CSP operating procedures and federal and local VA regulations. This file will be destroyed according to CSP policy well after the close of the study.

VA Central IRB

Access to the study data is restricted to individuals with CSP approval. Study team members must be properly credentialed research staff and must be compliant with VA security trainings (e.g. HIPAA, Rules of Behavior, and Good Clinical Practices). Once formal training is completed, user accounts for a study-specific web application utilizing a URL specific to the study to access and use the system and enter patient data will be activated for project management, study nursing staff, and call center staff. Accounts will be password protected and unique to each user. The account permissions will correspond with the users' functional study group. Furthermore, the permissions of the electronic systems are heavily restricted. The site Liaisons will not have access to study data. Only properly approved Coordinating Center personnel will have the ability to copy and export data. These individuals have received training on the local standard operating procedure (SOP) governing their permissions. Access to protected health information (PHI) will be restricted to individuals approved by CSP to have access to the data, including the study's mailing vendor.

At the Boston CSPCC the following staff will have access to all forms of PHI:

1. Center Director
2. Study Director
3. Project Management
4. Study Nurses
5. Data Management
6. Biostatisticians
7. Quality Assurance Officer
8. SAS/Database Programmer
9. Research Assistant
10. Clinical Applications Coordinator
11. Informatics Team

Periodic access control assessments will be made by Coordinating Center Quality Assurance personnel to verify that access is controlled and appropriate for personnel. In addition, the CSPCC QA group will provide continuing education on good clinical practices compliance and will evaluate clinical site operations for violations of VA policies including VA data security policies and GCP.

At the end of the study, the data for DCP will remain property of the Cooperative Studies Program and be stored and shared according to CSP guidelines and procedures. Retention and destruction of data will be conducted according to CSP operating procedures and federal and local VA regulations. This will include electronic data stored at the Boston CSPCC, and at the VA facility housing our servers.

VA Central IRB

Identifiable data will be kept according to CSP policy as outlined in the "CSP Guidelines for the Planning and Conduct of Cooperative Studies”.

XV. Human Subjects

A. Waiver of HIPAA authorization

A waiver of HIPAA authorization to use VA data to determine eligibility will be requested because the research could not practicably be conducted without access to and use of this information. In order to conduct the study, it is necessary to first be able to identify eligible patients so that the recruitment letter can be mailed to them.

We will also request IRB approval for a waiver of HIPAA authorization to collect data prospectively. Unlike traditional randomized clinical trials, a feature of the POC methodology is that data collection is performed by passive data capture. All data elements will be collected electronically from EHR/VISTA using both the Veteran’s Information and Computing Infrastructure (VINCI), a collaborative effort between the VA Office of Information Technology and the VA Office of Research and Development, and the Office of Information Technology’s Corporate Data Warehouse (CDW). Healthcare encounters outside of the VA will be captured using Medicare data. Data from the various VA databases will be linked together and maintained using a unique veteran identification number (not SSN). Access to the cross-walk file linking the participant’s identifiers and their study data will be restricted to approved personnel at the CSP coordinating center.

Because data abstraction is done electronically and not by staff perusing the electronic medical record we believe that a waiver of HIPAA authorization is justified and involves no more than minimal risk to the privacy of individuals. All protected health information, except participant address and treatment assignment, will remain within the VA. The information will only be shared with the mailing contractor using secure methods in alignment with VA policies. Moreover, it would be impractical to obtain a signed authorization from patients in this study for use of their health information. The PCP’s cannot obtain a written HIPAA authorization for research purposes from patients who are subjects in this study because they will not see the patient until weeks or months after the patient has been on the randomized treatment. The PCPs are not study team members. We plan to identify and disclose to participants in our information statement the health information to be collected and the specific databases from which it will be obtained.

In both instances, the patient eligibility screening and the prospective collection of patient data, the use of the requested information involves no more than minimal risk to the privacy of individuals based on the security measures used by the Boston CSPCC to protect the identifiers from improper use and disclosure, and to destroy the identifiers at the earliest opportunity consistent with conduct of the research. The requested information will not be reused or disclosed to any other person or entity, except as required by law, for authorized oversight of the research study, or for other research for which the use or disclosure of the requested information would be permitted by the Privacy Rule.

B. Basis for Waiver of Documentation of Informed Consent

VA Central IRB

Though we are not planning to obtain written informed consent, multiple measures are in place to ensure that all subjects are given all of the information they need in a manner that is understandable before they consent and throughout the study. Primary care providers will receive information through site liaisons as well as an introductory letter that is both mailed and emailed to their VA addresses prior to receiving the initial testpatient order in EHR. The initial testpatient order will be sent together with a progress note containing the Provider Information Sheet and by signing the testpatient order, the providers are consenting to participate. Provider participation is completely voluntary and minimal risk, facts that will be reiterated throughout this process.

Patients are sent a transcript of the consent and an explanatory letter with study contact information before the first phone call, full consent is obtained on the call, and they are informed of the randomized group after the call.

In this study, the relevant risks involved are those of changing therapy of half of the patients from hydrochlorothiazide to the equivalent dose of chlorthalidone, a very similar drug with the same indications and metabolic effects, plus the theoretical risk to confidentiality from compiling individual data.

Regarding the former, both drugs have been in use for more than 50 years, have long been available as generics, and are included in the VA national formulary in the same drug class: USP code CV 701, thiazide-type diuretics. The VA frequently selects one member of such a class to make available "on formulary" based on cost or other considerations, and VA Pharmacy and Therapeutics Committees will direct VA pharmacies to substitute drugs within a class when one drug becomes unavailable, so it is not uncommon that VA physicians and patients have limited choice within a class.

While thiazide-type diuretics affect some blood chemical levels as discussed above, these effects have not demonstrated adverse clinical impact that would affect their being the preferred treatment for hypertension. Furthermore, our plan to substitute an equivalent dose of chlorthalidone for the current dose of hydrochlorothiazide, i.e. half the number of milligrams, would be expected to have an effect on blood pressure and on blood chemical levels, small enough to be virtually indistinguishable from the variation in these parameters that would ensue with remaining on the prescribed hydrochlorothiazide (see Background and Rationale, parts F and I). We therefore propose that the study intervention qualifies as minimal risk. Because the two drugs are used interchangeably, and the choice between them is not influenced by any patient factors, but only by physician preference, randomization does not reduce individualization of patient care. Care remains personalized after randomization because the PCP manages the diuretic as usual. The patient's needs are thus not subordinated to the needs of the trial. Furthermore, because hypertension is a chronic condition, randomized patients will themselves be among those expected to benefit from the information gained from this study.

In their article "Randomized, controlled trials as minimal risk: an ethical analysis", Morris and Nelson (106) conclude that "A randomized, controlled trial poses no more than minimal risk only when all of the following five criteria are met: 1) genuine clinical equipoise exists; 2) all of the treatment options included in the research study fall within the current standard of care; 3) there is no currently available

VA Central IRB

treatment with a more favorable risk-benefit profile than the treatments included in the research study; 4) the nontherapeutic components of the research are safely under the minimal risk threshold; and 5) the research protocol provides sufficient latitude for treating physicians to individualize care when appropriate." We have designed this study to meet all of these criteria.

We believe that the Waiver of Documentation of Informed Consent that we are requesting is necessary to the successful completion of our study because obtaining the required sample size within the VA is only feasible if recruitment is maximally efficient, as described in the Feasibility section (XII.A). Published evidence demonstrates that requiring that written consent be returned by mail is likely to result in the loss of the great majority of patients who intend to consent (107-111). If requirement of returned written consent caused loss of most willing patients from our study, it is unlikely that it could be completed within the VA system.

Based on these considerations, we will request IRB approval of a Waiver of Documentation of Informed Consent. Under the terms we are requesting for the Waiver, patients would be recruited by mail, and the elements of consent obtained over the phone, a written summary of the consent will be sent to the patient, but a signed returned document would not be required.

Enrolled patients will be given study contact information for questions or withdrawal.

C. Engagement in Research

XVI. For this study, we consider the Site Liaison and the PCP's to be not engaged in research. The Site Liaison (sometimes called a "Champion" in other studies) serves to provide information to local site personnel about the study and relays information and questions back to the coordinating center, but in our view takes no action that qualifies as research. The PCPs facilitate implementation of the intervention (after patients provide consent and are randomized by study personnel) by signing an order sent to them by study personnel. The PCPs are themselves research subjects in whom we are studying the implementation of the protocol. Quality Control Procedures

Data that is extracted for the DCP will be cleaned and managed according to a rigorous data management plan that will be written in conjunction with the statistical analysis plan and the study operations manual (for chairs office and CSPCC personnel). However, the data will also be subject to quality control procedures. As a first line effort to ensure the validity of the data, 100% of the data elements collected from the first 10 individuals from each medical center will be subject to source verification and validation through chart review that will be done by an experienced clinician and clinical applications coordinator. In addition, chart review done through the creation of the algorithms to identify outcomes for the DCP will also be used to perform QC procedures on the data. In brief, a sample of the data will be verified periodically by the data management and clinical operations teams. If errors are identified they will be referred to the data management teams at the Boston CSPCC or at VINCI for resolution.

VA Central IRB

In addition, the quality management system (QMS) in place at the CSPCC will ensure further quality control for the DCP. The Quality Assurance Department of the CSPCC will subject the study to risk based audits according to internal SOPs for conducting risk based monitoring and auditing. These audits will evaluate the studys' compliance with QMS processes and procedures. If deviations or non-conformances are identified they will be remedied through the internal corrective action/preventive action system of the QMS.

XVII. Study Monitoring Plan

A. Introduction

The safety issues related to hydrochlorothiazide and chlorthalidone are well established in the medical literature and both diuretics are accepted first-line treatments for hypertension. Based on this information the study poses minimal risks to participants beyond the expected adverse events (AE) associated with the administration of either drug as part of "usual" care.

Monitoring side effects and adverse events in the traditional manner of usual clinical trials is not feasible for Point of Care studies since there are no site personnel and all data is captured passively through the EMR. In addition, real-time monitoring will neither provide new information regarding the safety of these two treatments nor assure adequate (or timely) safety of human subjects beyond that already done by the medical staff as part of routine medical care. Accordingly, we propose an alternative safety reporting plan for the DCP study that ensures protection of the participants and that complies with VHA Policy (i.e., VHA Handbooks 1200.05 and 1058.01). In brief, health providers will identify, monitor, and treat (as necessary) adverse events that occur during the course of the study. The CSPCC will identify expected adverse events (as described below) through the EMR and report them to the monitoring committees for the trial. The sections that follow describe in more detail the proposed safety monitoring and reporting plan for this trial.

B. Safety Monitoring Plan

The participant's physicians, nurses, and other health providers will continue their usual monitoring of the subject throughout the course of his/her treatment. If any treatments are indicated, they will be provided by health providers as a part of the participant's routine medical care. The CSPCC staff will collect safety data from the medical record from the time of consent through the end of the study period. The safety data will then be aggregated and classified according to ICD-10 codes.

Aggregated safety data will be reported to the Data Monitoring Committee, the study Executive Committee, and the VA Central IRB using the processes described in Section C. Please refer to Section XVIII Study Organization and Administration for a more detailed description of the oversight committees for the trial.

VA Central IRB

In addition to data culled from the EMR, the trial will allow for spontaneous reporting of events by study participants. The informed consent script and information sheet will include the contact information for the study call center should the participant wish to communicate safety concerns with the study team.

C. Safety Reporting

1. Adverse Events

Adverse events will be collected using the 21 CFR 312.32, International Conference on Harmonisation (ICH) for Clinical Safety Data Management (ICH-E2A), and CSP Global SOP 3.6 definitions. Adverse events (AEs) are defined by the 21 CFR 312.32 as "any untoward medical occurrence associated with the use of a drug in humans, whether or not considered drug related."

According to ICH-E2A,"an AE, therefore, can be any unfavorable or unintended sign (including an abnormal laboratory finding), symptom, or disease temporally associated with the study interventions."

Expected adverse events of interest related to diuretics will be culled from the EMR as part of the outcome ascertainment activities of this protocol. The expected AEs of interest will be:

1. Hospitalization for primary diagnosis of hypokalemia, hyponatremia, or renal failure
2. Renal failure (dialysis or renal transplant)
3. Other recorded hypokalemia (< 3.1 mEq/L), or hyponatremia (< 130 mEq/L)
4. New diabetes, defined as first use of an outpatient medication for diabetes
5. Acute gout episodes
6. New allergic reaction to thiazide-type diuretic to be found in EMR allergy data

These safety events are monitored and treated as part of routine medical care. These events will be identified by the CSPCC through the electronic medical record (EMR). Informatics staff at the Boston CSPCC will extract medical record data on all subjects and identify adverse events through ICD-10 codes, laboratory values, and medication files. Expected adverse events will be reported to the DMC in semi-annual reports. The DMC also reserves the right to request reporting on additional safety data not specified above, as relevant to the study. Data will be in the form of aggregated data tables detailing the frequencies of these events by blinded treatment group. These events will be reported to the Central IRB in blinded aggregate form at the time of continuing review.

Adverse events which develop into Serious Adverse Events, as defined below, will be reported as such.

2. Expected Serious Adverse Events

Serious adverse events (SAEs) are a subset of adverse events defined in 21 CFR 312.32(a) and VA Handbook 1058.01 paragraph 4(w), as follows:

Definition of SAE from CFR 312.32 (a): Serious adverse event. An adverse event or suspected adverse reaction is considered "serious" if, in the view of either the investigator or sponsor, it results in any of the following outcomes: death, a life-threatening adverse event, inpatient hospitalization or

VA Central IRB

prolongation of existing hospitalization, a persistent or significant incapacity or substantial disruption of the ability to conduct normal life functions, or a congenital anomaly/birth defect. Important medical events that may not result in death, be life-threatening, or require hospitalization may be considered serious when, based upon appropriate medical judgment, they may jeopardize the patient or subject and may require medical or surgical intervention to prevent one of the outcomes listed in this definition.

Definition of SAE according to VA Handbook 1058.01: An SAE is an AE in human research that results in death, a life-threatening experience, inpatient hospitalization, prolongation of hospitalization, persistent or significant disability or incapacity, congenital anomaly, or birth defect. An AE is also considered serious when medical, surgical, behavioral, social, or other intervention is needed to prevent such an outcome.

The intervention in this study is the switch from hydrochlorothiazide to an equivalent dose of chlorthalidone, two widely-used diuretics with well-known risk profiles. There are no safety events that are unanticipated in regard to these two drugs. Thus, serious adverse events defined above that are feasibly identified through the medical record will be reported in aggregate to the DMC at 6 month intervals and to the Central IRB at continuing review.

Expected serious adverse events will be reported to the IRB at the time of continuing review in aggregated data tables detailing the frequencies of these events. Study reports will also be circulated to appropriate members, including the study chairmen and DMC.

This study will not use MedDRA coding of AE and SAE data. The study team will define events using the data sources described above and categorize events by system organ class, major diagnostic category, or assessment type for reporting.

3. Unanticipated Problems Involving Risks to Subjects and Others

Any unanticipated problems involving risks to subjects or others (UPRs), but not qualifying as a serious adverse event by definition (such as errant distribution of study medication), will also be reported. Unanticipated problems related to the study design will be reported to the IRB in an expedited fashion (within 5 business days of identifying the problem). Possible events include failure to distribute study drug, patient continuing previous prescription while taking study drug, or patient randomized without provider knowledge. Informatics staff at the Boston CSPCC will extract medical record data on all subjects regularly allowing for the identification of UPRs at fixed intervals. Study reports will be circulated to appropriate members, including the study chairmen.

UPRs that result in an SAE, pursuant to the definition above, will be reported as an SAE to the VA Central IRB within 5 business days by the Boston CSP Coordinating Center after becoming aware of the event.

XVIII. Study Organization and Administration

A. Administration

The administrative structure of this study is similar to others in CSP and includes:

VA Central IRB

The Cooperative Studies Program (VA Central Office) establishes overall policies and procedures that are applied to all VA cooperative studies through the Study Chair's office and the CSPCC.

The CSPCC and the Study Chair's office jointly will perform the day-to-day scientific and administrative coordination of the study. These include developing and revising the study protocol; abstracting data from the national databases (CSPCC Only); ensuring the appropriate support for the participating centers; scheduling meetings and conference calls; answering questions about the protocol. The CSPCC will also prepare interim and final progress reports; and archive study data at the end of the study. Study progress reports will be produced every 6 months. Patient accrual, patient safety, and data quality will be monitored closely by the CSPCC, the study executive committee, and the DMC to ensure that the study is progressing satisfactorily. Further delineation of responsibilities will be documented in communications with the Study Chair's office.

The CSPCC will be responsible for monitoring and reporting the safety of trial participants through the review, assessment, and communication of adverse events and serious adverse events detected within the national data systems. The CSPCC will document trends and analyze safety data to prepare reports for various committees including the DMC, VA Central IRB (CIRB), Executive Committee(s), and Study Group meetings.

The CSPCC will work closely with the Minneapolis VAMC to track eligible participants as they move through the recruitment and enrollment workflow. The primary responsibilities for the callers include contacting patients by telephone to obtain verbal informed consent and receiving incoming patient phone calls.

The external mailing contractor will be primarily responsible for sending study communications to providers and patients. The mailing contractor will be sent regular mailing files compiled by the ProjectFlow application with participant name, address, and specific correspondence to be sent. PHI will be sent to the mailing contractor using secure methods in alignment with VA policies.

The CSP Clinical Research Pharmacy Coordinating Center (CSPCRPCC) will provide advice and consultation about drug-related matters, review safety information, and serve as a regulatory affairs expert and liaison to the FDA for regulatory matters.

The Clinical Sciences Scientific Evaluation Committee (CSSEC) reviews the scientific merit of all new cooperative study proposals and all ongoing cooperative studies. The committee is composed of both VA and non-VA clinical research scientists, most of whom have had experience in managing their own cooperative studies.

B. Monitoring

The following groups monitor the various aspects of the study. These committees will meet according to current Cooperative Studies Program guidelines.

The Executive Committee is responsible for the operations of the study, including protocol amendments, and overall management of the study. It will be headed by the Study Chair and Study Director and

VA Central IRB

consist of the study biostatistician, study project manager, CSP Center Director, selected VA experts, and outside consultants as needed. This committee will meet regularly to review blinded data (not broken down by treatment group), decide upon changes in the study, determine the fate of hospitals whose performance is substandard, initiate any subprotocols, and discuss publication of the study results. This Committee must grant permission before any study data may be used for presentation or publication.

The Technical Committee will advise the Executive Committee on informatics and database related issues pertaining to study operations. This committee will serve as subject matter experts (SMEs) for the study database, web application, ETL procedures and primary data source, the Corporate Data Warehouse. It will consist of a VINCI representative, Boston CSPCC Director of Informatics, and other informaticians/SMEs as needed.

The Data Monitoring Committee (DMC) will review the progress of the study and will monitor patient intake, outcomes, adverse events, and other issues related to patient safety. Interim, independent, and unbiased reviews of the study's ongoing progress will be provided. The DMC will consist of experts in the study's subject matter field(s), clinical trials, biostatistics, and ethics. These individuals will not be participants in the trial and will not have participated in the planning of the protocol. The DMC will consider safety or other circumstances as grounds for early termination, including either compelling internal or external evidence of treatment differences or the unfeasibility of addressing the study hypothesis (e.g., poor patient intake, poor adherence to the protocol).

At each of its meetings during the study period, the DMC will review the randomization rates and assess the difference between the actual and the projected rates, as well as the impact of these assessments on overall trial size. If the study enrollment is inadequate, the reasons for exclusion may be scrutinized and actions may be suggested. An assessment of whether the trial should be continued will be made followed by recommendations, as appropriate. All serious adverse events will be reported regularly to the DMC for review. Unexpected, related serious adverse events will be reported to the DMC as soon as they become known based upon the consensus of the Study Chair, the study biostatistician, the Study Director. The study biostatistician will provide the appropriate data to the DMC at specified intervals for this purpose. Conditional power estimates may be provided to the DMC to assist them in making their decisions and recommendations at their request. To help them make their assessment, the Study Chair and study biostatistician will furnish the Data Monitoring Committee with appropriate monitoring data before each meeting. The DMC makes recommendations after each meeting to the Director of the Cooperative Studies Program (CSP)Service about whether the study should continue or be stopped.

The VA Central IRB will be the IRB of record for all VA sites. They will monitor the study's serious adverse events on a continual basis. They will conduct annual reviews of the study. In addition, some study materials (such as subject correspondence and protocol changes) will have to be reviewed by the VA CIRB, and approved prior to implementation.

XIX. The CSPCC Human Rights Committee (HRC) is composed primarily of lay people and is responsible for ensuring that patients' rights and safety are upheld prior to study initiation and during the conduct of the study. The committee reviews all new protocols, periodically

makes site visits to participating centers to monitor firsthand the progress of the study, and may be asked to review any ethical and human rights issues that arise during the conduct of the study. Publications

A. Publication policy

It is the policy of the CSP that outcome data will not be revealed to the participating investigators until the data collection phase of the study is completed. This policy safeguards against possible biases affecting the data collection. All presentations and publications from this study will be done in accordance with current CSP Guidelines, including the Authorship Policy. The most current version of the Guidelines should be referenced when planning any study publication.

The presentation or publication of any or all data collected by participating investigators on patients entered into the VA Cooperative Study is under the direct control of the study's Executive Committee. No individual participating investigator has any inherent right to perform analyses or interpretations or to make public presentations or seek publication of any or all of the data other than under the auspices and approval of the Executive Committee.

The Executive Committee has the authority to establish one or more publication subgroups of investigators and members of the Executive Committee for producing scientific presentations and publications. Authors with VA appointments must list their VA affiliation first. The VA contributions to the research project should be acknowledged in all written and oral presentations of the research results, including scientific articles, news releases, news conferences, public lectures, and media interviews.

All study reports and journal manuscripts must be reviewed and approved by the Boston CSPCC Director prior to submission for publication. After approval for submission is granted by the Boston CSPCC Director, VA Central Office must be notified upon acceptance of any publications. This includes minor publications such as abstracts and poster presentations.

B. Planned Publications

A list of planned publications is below:

I. Editorial: The first large clinically integrated VA trial

II. Design of the DCP

III. Ascertainment of urgent revascularization from administrative data

IV. Ascertainment of acute heart failure episodes from administrative data

V. Ascertainment of stroke episodes from administrative data

VI. Use of and experiences with telephone informed consent

VII. Challenges of embedding DCP into the electronic medical record

VA Central IRB

VIII. Main outcomes paper

IX. Blood pressure control and drug compliance in the DCP

X. Blood chemistries in the DCP

VA Central IRB

XX. Provider Experience Survey

In order to improve the design of future point-of-care trials, a survey was designed to query providers that consented to participate in the CSP 597 study about their experience with the study (Appendix 21). The questions were designed to assess if education about the study was adequate and delivered in a convenient format, how interested providers are in the study question, time burden of study participation, and level of comfort with a pragmatic trial design. Demographic information is also requested. Survey results will be captured in a VA approved platform.. Completion of the survey is voluntary. The study will be reviewed by CIRB and the Organizational Assessment Committee (OAC) before being distributed to consented CSP 597 providers. Provider survey results will be made available through clinicaltrials.gov. Qualitative analysis will be performed for open text responses and sub-questions. Dichotomous responses and survey responses on a Likert scale will be analyzed using quantitative methods.

An additional interview will be made available to consented providers at Minneapolis VAHCS (Appendix 25). This interview will take place in person or via teleconference. Interview questions will focus on the providers individual experience throughout DCP and allow providers to give less structured feedback on the consent and randomization workflows. The aim is to further help structure future POC studies. All results will de-identified and made available on clinicaltrials.gov

XX. References

1. http://www.cdc.gov/bloodpressure/hypertension_iom.htm

2. http://www.webmd.com/news/20110420/the-10-most-prescribed-drugs

3. http://www.healthquality.va.gov/hypertension/htn04_pdf1.pdf

4. Chobanian AV, Bakris GL, Black HR, et al. The Seventh Report of the Joint National Committee on Prevention, Detection, Evaluation, and Treatment of High Blood Pressure: the JNC 7 report. JAMA 2003;289:2560-72.

5. Psaty BM, Lumley T, Furberg CD, Schellenbaum G, Pahor M, Alderman MH, Weiss NS. Health outcomes associated with various antihypertensive therapies used as first-line agents: a network meta-analysis. JAMA 2003;289:2534-44.

6. Messerli FH, Bangalore S. Half a century of hydrochlorothiazide: facts, fads, fiction, and follies. Am J Med 2011;124:896-9.

7. Ernst ME, Lund BC. Renewed interest in chlorthalidone: evidence from the Veterans Health Administration. J Clin Hypertens (Greenwich). 2010;12:927-34.

8. Kaplan NM. Chlorthalidone versus hydrochlorothiazide: a tale of tortoises and a hare. Hypertension 2011;58:994-5.

9. Mitka M. Experts argue not all diuretics the same. JAMA 2007;298:31.

VA Central IRB

10. Kaplan NM. The case for low dose diuretic therapy. Am J Hypertens 1991;4:970-1.

11. Materson BJ, Cushman WC, Goldstein G, Reda DJ, Freis ED, Ramirez EA, Talmers FN, White TJ, Nunn S, Chapman RH, et al. Treatment of hypertension in the elderly: I. Blood pressure and clinical changes. Results of a Department of Veterans Affairs Cooperative Study. Hypertension 1990;15:348-60.

12. Cushman WC, Khatri I, Materson BJ, Reda DJ, Freis ED, Goldstein G, Ramirez EA, Talmers FN, White TJ, Nunn S, et al. Treatment of hypertension in the elderly. III. Response of isolated systolic hypertension to various doses of hydrochlorothiazide: results of a Department of Veterans Affairs cooperative study. Department of Veterans Affairs Cooperative Study Group on Antihypertensive Agents. Arch Intern Med 1991;151:1954-60.

13. Materson BJ, Oster JR, Michael UF, Bolton SM, Burton ZC, Stambaugh JE, Morledge J. Dose response to chlorthalidone in patients with mild hypertension. Efficacy of a lower dose. Clin Pharmacol Ther 1978;24:192-8.

14. Morledge JH, Ettinger B, Aranda J, McBarron F, Barra P, Gorwit J, Davidov M. Isolated systolic hypertension in the elderly. A placebo-controlled, dose-response evaluation of chlorthalidone. J Am Geriatr Soc 1986;34:199-206.

15. Neff KM, Nawarskas JJ. Hydrochlorothiazide versus chlorthalidone in the management of hypertension. Cardiol Rev 2010;18:51-6.

16. James PA, Oparil S, Carter BL, et al. 2014 evidence-based guideline for the management of high blood pressure in adults: report from the panel members appointed to the Eighth Joint National Committee (JNC 8). JAMA 2014;311:507-20.

17. Dorsch MP, Gillespie BW, Erickson SR, Bleske BE, Weder AB. Chlorthalidone reduces cardiovascular events compared with hydrochlorothiazide: a retrospective cohort analysis. Hypertension 2011;57:689-94.

18. Ernst ME, Neaton JD, Grimm RH Jr, Collins G, Thomas W, Soliman EZ, Prineas RJ; Multiple Risk Factor Intervention Trial Research Group. Long-term effects of chlorthalidone versus hydrochlorothiazide on electrocardiographic left ventricular hypertrophy in the multiple risk factor intervention trial. Hypertension 2011;58:1001-7.

19. Wing LM, Reid CM, Ryan P, Beilin LJ, Brown MA, Jennings GL, Johnston CI, McNeil JJ, Macdonald GJ, Marley JE, Morgan TO, West MJ; Second Australian National Blood Pressure Study Group. A comparison of outcomes with angiotensin-converting--enzyme inhibitors and diuretics for hypertension in the elderly. N Engl J Med 2003;348:583-92.

20. ALLHAT Collaborative Research Group. Major outcomes in high-risk hypertensive patients randomized to angiotensin-converting enzyme inhibitor or calcium channel blocker vs diuretic:

VA Central IRB

The Antihypertensive and Lipid-Lowering Treatment to Prevent Heart Attack Trial (ALLHAT). JAMA. 2002;288:2981-97.

21. Roush GC, Holford TR, Guddati AK. Chlorthalidone compared with hydrochlorothiazide in reducing cardiovascular events: systematic review and network meta-analyses. Hypertension 2012;59:1110-7.

22. Carter BL, Ernst ME, Cohen JD. Hydrochlorothiazide versus chlorthalidone: evidence supporting their interchangeability. Hypertension 2004;43:4-9.

23. Ernst ME, Carter BL, Goerdt CJ, Steffensmeier JJ, Phillips BB, Zimmerman MB, Bergus GR. Comparative antihypertensive effects of hydrochlorothiazide and chlorthalidone on ambulatory and office blood pressure. Hypertension 2006;47:352-8.

24. Kwon BJ, Jang SW, Choi KY, Kim DB, Cho EJ, Ihm SH, Youn HJ, Kim JH. Comparison of the efficacy between hydrochlorothiazide and chlorthalidone on central aortic pressure when added on to candesartan in treatment-naïve patients of hypertension. Hypertens Res. 2013;36:79-84.

25. https://vaww.cmopnational.va.gov/cmop/PBM/Clinical Guidance/Archived Criteria, Guidelines and Reviews/Clinical Recommendations (Archive)/Thiazides in Hypertension, Review of Recent Evidence (Archived Dec 2014).doc

26. Ernst ME, Carter BL, Zheng S, Grimm RH Jr. Meta-analysis of dose-response characteristics of hydrochlorothiazide and chlorthalidone: effects on systolic blood pressure and potassium. Am J Hypertens 2010;23:440-6.

27. Peterzan MA, Hardy R, Chaturvedi N, Hughes AD. Meta-analysis of dose-response relationships for hydrochlorothiazide, chlorthalidone, and bendroflumethiazide on blood pressure, serum potassium, and urate. Hypertension 2012;59:1104-9.

28. Lund BC, Ernst ME. The comparative effectiveness of hydrochlorothiazide and chlorthalidone in an observational cohort of veterans. J Clin Hypertens (Greenwich). 2012 Sep;14(9):623-9.

29. Dhalla IA, Gomes T, Yao Z, Nagge J, Persaud N, Hellings C, Mamdani MM, Juurlink DN. Chlorthalidone versus hydrochlorothiazide for the treatment of hypertension in older adults: a population-based cohort study. Ann Intern Med 2013;158:447-55.

30. Bakris GL, Sica D, White WB, Cushman WC, Weber MA, Handley A, Song E, Kupfer S. Antihypertensive efficacy of hydrochlorothiazide vs chlorthalidone combined with azilsartan medoxomil. Am J Med. 2012;125:1229.e1-1229.e10.

31. Cushman WC, Bakris GL, White WB, Weber MA, Sica D, Roberts A, Lloyd E, Kupfer S. Azilsartan medoxomil plus chlorthalidone reduces blood pressure more effectively than olmesartan plus hydrochlorothiazide in stage 2 systolic hypertension. Hypertension. 2012;60:310-8.

VA Central IRB

32. Ernst ME, Moser M. Use of diuretics in patients with hypertension. N Engl J Med. 2009 Nov 26;361(22):2153-64.

33. Dolan E, Stanton A, Thijs L, Hinedi K, Atkins N, McClory S, Den Hond E, McCormack P, Staessen JA, O'Brien E. Superiority of ambulatory over clinic blood pressure measurement in predicting mortality: the Dublin outcome study. Hypertension. 2005 Jul;46(1):156-61.

34. Fagard RH, Celis H, Thijs L, Staessen JA, Clement DL, De Buyzere ML, De Bacquer DA. Daytime and nighttime blood pressure as predictors of death and cause-specific cardiovascular events in hypertension. Hypertension. 2008 Jan;51(1):55-61.

35. Woodman R, Brown C, Lockette W. Chlorthalidone decreases platelet aggregation and vascular permeability and promotes angiogenesis. Hypertension. 2010 Sep;56(3):463-70.

36. Kurtz TW. Chlorthalidone: don't call it "thiazide-like" anymore. Hypertension 2010;56:335-7.

37. Rodenburg EM, Hoorn EJ, Ruiter R, Lous JJ, Hofman A, Uitterlinden AG, Stricker BH, Visser LE. Thiazide-Associated Hyponatremia: A Population-Based Study. Am J Kidney Dis. 2013 Apr 18. [Epub ahead of print]

38. Alderman MH, Piller LB, Ford CE, et al; Antihypertensive and Lipid-Lowering Treatment to Prevent Heart Attack Trial Collaborative Research Group. Clinical significance of incident hypokalemia and hyperkalemia in treated hypertensive patients in the antihypertensive and lipid-lowering treatment to prevent heart attack trial. Hypertension. 2012;59(5):926-33.

39. Barzilay JI, Davis BR, Cutler JA, Pressel SL, Whelton PK, Basile J, Margolis KL, Ong ST, Sadler LS, Summerson J; ALLHAT Collaborative Research Group. Fasting glucose levels and incident diabetes mellitus in older nondiabetic adults randomized to receive 3 different classes of antihypertensive treatment: a report from the Antihypertensive and Lipid-Lowering Treatment to Prevent Heart Attack Trial (ALLHAT). Arch Intern Med. 2006 Nov 13;166(20):2191-201.

40. Barzilay JI, Davis BR, Pressel SL, Cutler JA, Einhorn PT, Black HR, Cushman WC, Ford CE, Margolis KL, Moloo J, Oparil S, Piller LB, Simmons DL, Sweeney ME, Whelton PK, Wong ND, Wright JT Jr; ALLHAT Collaborative Research Group. Long-term effects of incident diabetes mellitus on cardiovascular outcomes in people treated for hypertension: the ALLHAT Diabetes Extension Study. Circ Cardiovasc Qual Outcomes. 2012 Mar 1;5(2):153-62.

41. McAdams DeMarco MA, Maynard JW, Baer AN, Gelber AC, Young JH, Alonso A, Coresh J. Diuretic use, increased serum urate levels, and risk of incident gout in a population-based study of adults with hypertension: the Atherosclerosis Risk in Communities cohort study. Arthritis Rheum. 2012 Jan;64(1):121-9.

42. Siegel D, Hulley SB, Black DM, Cheitlin MD, Sebastian A, Seeley DG, Hearst N, Fine R. Diuretics, serum and intracellular electrolyte levels, and ventricular arrhythmias in hypertensive men. JAMA 1992;267:1083-9.

VA Central IRB

43. Mount DB, et al. Clinical manifestations and treatment of hypokalemia. UpToDate. Accessed July 2013.

44. Roush GC, Buddharaju V, Ernst ME, Holford TR. Chlorthalidone: Mechanisms of Action and Effect on Cardiovascular Events. Curr Hypertens Rep. 2013 Jul 10. [Epub ahead of print]

45. Rahman M, Pressel S, Davis BR, et al. Renal outcomes in high-risk hypertensive patients treated with an angiotensin-converting enzyme inhibitor or a calcium channel blocker vs a diuretic: a report from the Antihypertensive and Lipid-Lowering Treatment to Prevent Heart Attack Trial (ALLHAT). Arch Intern Med. 2005 Apr 25;165(8):936-46.

46. Rahman M, Ford CE, Cutler JA, et al; ALLHAT Collaborative Research Group. Long-term renal and cardiovascular outcomes in Antihypertensive and Lipid-Lowering Treatment to Prevent Heart Attack Trial (ALLHAT) participants by baseline estimated GFR. Clin J Am Soc Nephrol. 2012 Jun;7(6):989-1002.

47. Prevention of stroke by antihypertensive drug treatment in older persons with isolated systolic hypertension. Final results of the Systolic Hypertension in the Elderly Program (SHEP). SHEP Cooperative Research Group. JAMA 1991;265:3255-64.

48. Hueskes BA, Roovers EA, Mantel-Teeuwisse AK, Janssens HJ, van de Lisdonk EH, Janssen M. Use of diuretics and the risk of gouty arthritis: a systematic review. Semin Arthritis Rheum. 2012 Jun;41(6):879-89.

49. Mortality findings for stepped-care and referred-care participants in the hypertension detection and follow-up program, stratified by other risk factors. The Hypertension Detection and Follow-up Program Cooperative Research Group. Prev Med 1985;14:312-35.

50. Wilson L, Nair KV, Saseen JJ. Comparison of New-Onset Gout in Adults Prescribed Chlorthalidone vs Hydrochlorothiazide for Hypertension. J Clin Hypertens. 2014 Sep 25. doi: 10.1111/jch.12413. [Epub ahead of print]

51. Khosla N, Chua DY, Elliott WJ, Bakris GL. Are chlorthalidone and hydrochlorothiazide equivalent blood-pressure-lowering medications? J Clin Hypertens 2005;7:354-6.

52. Matthews KA, Brenner MJ, Brenner AC. Evaluation of the Efficacy and Safety of a Hydrochlorothiazide to Chlorthalidone Medication Change in Veterans With Hypertension. Clin Ther. 2013 Aug 28.[Epub ahead of print]

53. van Blijderveen JC, Straus SM, Rodenburg EM, Zietse R, Stricker BH, Sturkenboom MC, Verhamme KM. Risk of hyponatremia with diuretics: chlorthalidone versus hydrochlorothiazide. Am J Med 2014;127:763-71.

54. Roush GC, Buddharaju V, Ernst ME. Is chlorthalidone better than hydrochlorothiazide in reducing cardiovascular events in hypertensives? Curr Opin Cardiol 2013;28:426-32.

VA Central IRB

55. Dhalla IA, Mamdani MM, Juurlink DN. Chlorthalidone versus hydrochlorothiazide. Ann Intern Med. 2013;158:923-4.

56. Einhorn PT, Cushman WC, Whelton PK; ALLHAT Collaborative Research Group. Chlorthalidone versus hydrochlorothiazide. Ann Intern Med 2013;158:922-3.

57. Asche C, Yang J, Yu S, Hagan M. A profiling of hypertension patients treated with chlorthalidone or hydrochlorothiazide. Value Health. 2013 May;16(3):A295.

58. Krause T, Lovibond K, Caulfield M, McCormack T, Williams B; Guideline Development Group. Management of hypertension: summary of NICE guidance. BMJ 2011;343:d4891.

59. Mancia G, Fagard R, Narkiewicz K, et al; Task Force Members. 2013 ESH/ESC Guidelines for the management of arterial hypertension: the Task Force for the management of arterial hypertension of the European Society of Hypertension (ESH) and of the European Society of Cardiology (ESC). J Hypertens 2013;31:1281-357.

60. Floyd JS, Psaty BM. Observational Comparative Effectiveness Studies of Drug Therapies: High-Quality Answers or Important Clinical Questions? Comment on "Comparative Effectiveness of 2 β-Blockers in Hypertensive Patients". Arch Intern Med 2012:172:1412-4.

61. Lauer MS, D'Agostino RB. The randomized registry trial — the next disruptive technology in clinical research? N Engl J Med 2013; 369:1579-1581.

62. Jansen JP, Fleurence R, Devine B, Itzler R, Barrett A, Hawkins N, Lee K, Boersma C, Annemans L, Cappelleri JC. Interpreting indirect treatment comparisons and network meta-analysis for health-care decision making: report of the ISPOR Task Force on Indirect Treatment Comparisons Good Research Practices: part 1. Value Health 2011;14:417-28.

63. Pletcher MJ, Lo B, Grady D. Informed consent in randomized quality improvement trials: a critical barrier for learning health systems. JAMA Intern Med 2014;174:668-70.

64. Peto R, Collins R, Gray R. Large-scale randomized evidence: large, simple trials and overviews of trials. J Clin Epidemiol. 1995 Jan;48(1):23-40.

65. Vickers AJ, Scardino PT. The clinically-integrated randomized trial: proposed novel method for conducting large trials at low cost. Trials. 2009;10:14.

66. van Staa TP, Goldacre B, Gulliford M, Cassell J, Pirmohamed M, Taweel A, Delaney B, Smeeth L. Pragmatic randomised trials using routine electronic health records: putting them to the test. BMJ. 2012 Feb 7;344:e55.

67. Fiore LD, Brophy M, Ferguson RE, D'Avolio L, Hermos JA, Lew RA, Doros G, Conrad CH, O'Neil JA Jr, Sabin TP, Kaufman J, Swartz SL, Lawler E, Liang MH, Gaziano JM, Lavori PW. A point-of-care clinical trial comparing insulin administered using a sliding scale versus a weight-based regimen. Clin Trials. 2011;8:183-95.

VA Central IRB

68. D'Avolio L, Ferguson R, Goryachev S, Woods P, Sabin T, O'Neil J, Conrad C, Gillon J, Escalera J, Brophy M, Lavori P, Fiore L. Implementation of the Department of Veterans Affairs' first point-of-care clinical trial. J Am Med Inform Assoc. 2012 Jun 1;19(e1):e170-e176. Epub 2012 Feb 24.

69. IOM Roundtable on Evidence-Based Medicine. Appendix A. Learning what works best: the nation's need for evidence on comparative effectiveness in health care: an issue overview. In: Learning What Works: Infrastructure Required for Comparative Effectiveness Research: Workshop Summary. Institute of Medicine (US) Roundtable on Value & Science-Driven Health Care. Washington (DC): National Academies Press (US); 2011. http://www.ncbi.nlm.nih.gov/books/NBK64784/

70. Eapen ZJ, Lauer MS, Temple RJ. The imperative of overcoming barriers to the conduct of large, simple trials. JAMA 2014;311:1397-8.

71. Smith DH, Neutel JM, Lacourcière Y, Kempthorne-Rawson J. Prospective, randomized, open-label, blinded-endpoint (PROBE) designed trials yield the same results as double-blind, placebo-controlled trials with respect to ABPM measurements. J Hypertens. 2003 Jul;21(7):1291-8.

72. Kerr EA, Lucatorto MA, Holleman R, Hogan MM, Klamerus ML, Hofer TP; VA Diabetes Quality Enhancement Research Initiative (QUERI) Workgroup on Clinical Action Measures. Monitoring performance for blood pressure management among patients with diabetes mellitus: too much of a good thing? Arch Intern Med. 2012;172:938-45.

73. Berry SD, Kiel DP. Treating hypertension in the elderly: should the risk of falls be part of the equation? JAMA Intern Med 2014;174:596-7.

74. Nord J, Stults B, Rose R, Underwood AE, Williams T, West G, Huhtala T, Milne CK. Optimizing office blood pressure measurement at a VAMC. Fed Pract 2012;29(5):35-39.

75. Kim JW, Bosworth HB, Voils CI, Olsen M, Dudley T, Gribbin M, Adams M, Oddone EZ. How well do clinic-based blood pressure measurements agree with the mercury standard? J Gen Intern Med 2005;20:647-9.

76. Freeman MK, White W, Iranikhah M. Tablet splitting: a review of the clinical and economic outcomes and patient acceptance. Consult Pharm 2012;27:421-30.

77. Kalsbeek WD, Botman SL, Massey JT, Liu PW. Cost-Efficiency and the Number of Allowable Call Attempts in the National Health Interview Survey. Journal of Official Statistics 1994;10(2): 133–52

78. Hörngren J, Lundquist P, Westling S. Effects of number of call attempts on nonresponse rates and nonresponse bias – result from some case studies at Statistics Sweden. Statistics Canada’s International Symposium Series: Proceedings, 2009. http://www.statcan.gc.ca/pub/11-522-x/2008000/article/10999-eng.pdf

VA Central IRB

79. Fowler FJ, Jr., Gallagher PM, Stringfellow VL, Zaslavsky AM, Thompson JW, Cleary PD. Using telephone interviews to reduce nonresponse bias to mail surveys of health plan members. Med Care. 2002;40:190-200.

80. Fu SS, van Ryn M, Burgess DJ, Nelson D, Clothier B, Thomas JL, Nyman JA, Joseph AM. Proactive tobacco treatment for low income smokers: study protocol of a randomized controlled trial. BMC Public Health 2014;14:337.

81. Koepsell T, McGuire V, Longstreth W, Nelson L, Belle G. Randomized trial of leaving messages on telephone answering machines for control recruitment in an epidemiologic study. Am J Epidemiol. 1996;144:704-706.

82. Andrade SE, Kahler KH, Frech F, Chan KA. Methods for evaluation of medication adherence and persistence using automated databases. Pharmacoepidemiol Drug Saf. 2006;15:565-74.

83. Sacco RL, Kasner SE, Broderick JP, Caplan LR, Connors JJ, Culebras A, Elkind MSV, George MG, Hamdan AD, Higashida RT, Hoh BL, Janis LS, Kase CS, Kleindorfer DO, Lee J-M, Moseley ME, Peterson ED, Turan TN, Valderrama AL, Vinters HV. An updated definition of stroke for the 21st century: a statement for healthcare professionals from the American Heart Association/American Stroke Association. Stroke. 2013; 44:2064-2089.

84. Thygesen K, Alpert JS, Jaffe AS, Simoons ML, Chaitman BR, White HD; Joint ESC/ACCF/AHA/WHF Task Force for the Universal Definition of Myocardial Infarction, et al. Third universal definition of myocardial infarction. Circulation. 2012;126:2020-35.

85. Braunwald E, Domanski MJ, Fowler SE, et al; PEACE Trial Investigators. Angiotensin-converting-enzyme inhibition in stable coronary artery disease. N Engl J Med 2004;351:2058-68.

86. Morrow DA, Braunwald E, Bonaca MP, et al; TRA 2P–TIMI 50 Steering Committee and Investigators. Vorapaxar in the secondary prevention of atherothrombotic events. N Engl J Med 2012;366:1404-13.

87. Tricoci P, Huang Z, Held C, et al; TRACER Investigators. Thrombin-receptor antagonist vorapaxar in acute coronary syndromes. N Engl J Med 2012;366:20-33.

88. White WB, Bakris GL, Bergenstal RM, Cannon CP, Cushman WC, et al. EXamination of cArdiovascular outcoMes with alogliptIN versus standard of carE in patients with type 2 diabetes mellitus and acute coronary syndrome (EXAMINE): a cardiovascular safety study of the dipeptidyl peptidase 4 inhibitor alogliptin in patients with type 2 diabetes with acute coronary syndrome. Am Heart J 2011;162:620-6.

89. Einhorn PT, Davis BR, Massie BM, Cushman WC, Piller LB, Simpson LM, Levy D, Nwachuku CE, Black HR; ALLHAT Collaborative Research Group. The Antihypertensive and Lipid Lowering Treatment to Prevent Heart Attack Trial (ALLHAT) Heart Failure Validation Study: diagnosis and prognosis. Am Heart J. 2007 Jan;153(1):42-53.

VA Central IRB

90. Go AS, Mozaffarian D, Roger VL, et al; AHA Statistics Committee and Stroke Statistics Subcommittee. Heart disease and stroke statistics--2013 update: a report from the American Heart Association. Circulation 2013;127:e6-e245.

91. Kannel WB. Blood pressure as a cardiovascular risk factor: prevention and treatment. JAMA 1996;275:1571-6.

92. Roger VL, Weston SA, Redfield MM, Hellermann-Homan JP, Killian J, Yawn BP, Jacobsen SJ. Trends in heart failure incidence and survival in a community based population. JAMA 2004;292:344-50.

93. Hicks KA, Hung HMJ, Mahaffey KW, et al. Standardized definitions for cardiovascular and stroke end point events in clinical trials. FDA draft report, 2012.

94. Ron E, Carter R, Jablon S, Mabuchi K. Agreement between death certificate and autopsy diagnoses among atomic bomb survivors. Epidemiology. 1994 Jan;5(1):48-56.

95. Sesso HD, Gaziano JM, Glynn RJ, Buring JE. Value of an Endpoints Committee versus the use of nosologists for validating cause of death. Contemp Clin Trials.2006 Aug;27(4): 333-9.

96. German RR, Fink AK, Heron M, et al. The accuracy of cancer mortality statistics based on death certificates in the United States. Cancer epidemiology 2011;35(2):126-31.

97. Prieto-Merino D, Smeeth L, Staa TP, Roberts I. Dangers of non-specific composite outcome measures in clinical trials. BMJ 2013;347:f6782.

98. McKee PA, Castelli WP, McNamara PM, Kannel WB. The natural history of congestive heart failure: the Framingham study. N Engl J Med. 1971;285:1441–6.

99. Sohn MW, Arnold N, Maynard C, Hynes DM. Accuracy and completeness of mortality data in the Department of Veterans Affairs. Popul Health Metr. 2006 Apr 10;4:2.

100. Fletcher RD, Amdur RL, Kolodner R, McManus C, Jones R, Faselis C, Kokkinos P, Singh S, Papademetriou V. Blood pressure control among US veterans: a large multiyear analysis of blood pressure data from the Veterans Administration health data repository. Circulation 2012;125:2462-8.

101. ACCORD Study Group, Cushman WC, Evans GW, Byington RP, et al. Effects of intensive blood-pressure control in type 2 diabetes mellitus. N Engl J Med. 2010;362:1575-85(+ online supplement).

102. Jennison, C and Turnbull, B. Group Sequential Methods with Applications to Clinical Trials. Boca Raton, Fl: Chapman & Hall/CRC Interdisciplinary Statistics, 2000.

103. Daniels MJ and Hogan JW. Missing Data in Longitudinal Studies: Strategies for Bayesian Modeling and Sensitivity Analysis. Boca Raton, Fl: Chapman and Hall/CRC, 2008.

VA Central IRB

104. Beyersmann J, Allignol A, and Schumacher M. Competing Risks and Multistate Models with R. New York, NY: Springer, 2012.

105. http://answers.hhs.gov/ohrp/questions/7276

106. Morris MC; Nelson RM. Randomized, controlled trials as minimal risk: An ethical analysis. Crit Care Med 2007; 35:940–4.

107. Nelson K, Garcia RE, Brown J, Mangione CM, Louis TA, Keeler E, Cretin S. Do patient consent procedures affect participation rates in health services research? Med Care 2002;40:283-8.

108. Cann CI, Rothman KJ. IRBs and epidemiologic research: how inappropriate restrictions hamper studies. IRB 1984;6(4):5-7.

109. Armstrong D, Kline-Rogers E, Jani SM, Goldman EB, Fang J, Mukherjee D, Nallamothu BK, Eagle KA. Potential impact of the HIPAA privacy rule on data collection in a registry of patients with acute coronary syndrome. Arch Intern Med 2005;165:1125-9.

110. Kaiser J. Patient privacy. Rule to protect records may doom long-term heart study. Science 2006;311:1547-8.

111. Ellickson PL, Hawes JA. An assessment of active versus passive methods for obtaining parental consent. Eval Rev 1989;13:45-55.

VA Central IRB

CSP 597: The Diuretic Comparison Project (DCP) Protocol – Summary of Changes

Version	Effective Date	Description of Changes
**2.2**	March 16, 2016	• Removal of reference to the 'ART Database' for identification of allergic reactions (p. 28, 53). The study will collect all data from the Corporate Data Warehouse (CDW) and Electronic Medical Record (EMR); ART is a specific VistA package that will not be used<br>• Removal of "(2 weeks)" from sentence on p. 33. This protocol indicates monthly data pulls elsewhere, thus we removed the reference to "2 weeks" for consistency<br>• Clarified CPRS tools used for randomization (order vs. progress note). CPRS changes allow for improved communication with PCPs and consistency between randomization arms.<br>• Added language for ICD-10 codes
**2.3**	July 07, 2016	• Revised references on p29 to fix typos.<br>• Removed Appendix D. CPRS Screens v2.1 from protocol<br>• Clarified voicemail procedures. The voicemail procedures were amended to allow callers to leave a voicemail after a caller returns a previous voicemail to ensure good customer service.
**2.4**	January 05, 2017	• Edited opt-out language to remove references to opt-out card and to explain new voicemail box phone tree system<br>• Fixed typo on p.36 about Type I errors<br>• Clarified consent procedures, allowing research staff to contact, consent, and withdraw patients on follow-up calls that originated with the Call Center<br>• Added a pre-eligibility check prior to sending randomization view alerts to providers<br>• Removed vascular access for dialysis from the definition of renal failure<br>• Redefined the definition of stroke from WHO definition to AHA definition
**2.5**	May 08, 2017	• Clarified language on external mail contractor
**2.6**	June 28, 2017	• Removed reference to health factors which are not being utilized<br>• Updated definition of urgent coronary revascularization to include unstable angina<br>• Added definition of unstable angina<br>• Clarified 'Ascertainment of Outcome Data' section to reflect the current process being utilized<br>• Removed specific outcome definition from protocol to be an appendix (Outcome Definitions)<br>• Updated source for vital status and cause of death data<br>• Clarified 'Data Management and Data Security Plans' section to better describe security protocols and data access

		• Added language noting that the DMC can request additional safety data not specified in the protocol<br>• Removed language regarding monthly medical record data
**2.7**	August 23, 2017	• Added language permitting the study team to provide education to sites as well as the site liaison<br>• Added a reminder email to providers (See Appendix A.8)
**2.8**	October 02, 2017	• Added language changing that voicemails will be left on the 1st, 8th and 14th call to Veterans
**2.9**	May 01, 2018	• Generalized call center language to include trained study staff<br>• Added that we will contact pharmacists during site rollout<br>• Added the option to call a patient an additional 3 times if they contact the study team after the 15th call<br>• Added information on temporary and permanent ineligibility<br>• Replaced "encrypted mail" as the method to send secure information to the mail center to "secure method that meets VA requirements"<br>• Expanded planned publications section
**3.0**	July 06, 2018	• Changed PI from Dr. Frank Lederle to Dr. Areef Ishani
**3.1**	October 23, 2018	• Added second testpatient view alert order to request provider participation to providers that have previously declined, to be sent eight weeks after original order
**3.2**	November 23, 2018	• Removed Canandaigua VAMC as the call center
**3.3**	February 13, 2019	• Added language about email or texting<br>• Replaced reference to CSRD with CSP
**3.4**	May 06, 2019	• Changed MAVERIC to Boston CSPCC<br>• Edited voicemail language to change voicemails to be on a time scale (months) instead of a call scale<br>• Added language to potentially resend initial mailing materials (A2) to Veterans after 5 calls
**3.5**	June 06, 2019	• Added language about Spanish mailings and Spanish speaking callers
**3.6**	December 18, 2019	• Added an option to use video for site education
**3.7**	June 24, 2020	• Removed Dr. William Cushman as Co-Chair. Removed him in header line
**3.8**	September 03, 2020	• Edited language about reaffirmation calls to include all study team members
**3.9**	November 25, 2020	• Added language about dual enrollment<br>• Exchanged CRPS for VA HER<br>• Changed language to make temporarily ineligible letters optional
**4.0**	March 23, 2021	• Updated recruitment number, study timeline, and follow-up duration<br>• Added provider survey
**4.1**	July 28, 2021	• Added end of recruitment letters
**4.2**	October 08, 2021	• Removed QA component from outcomes<br>• Removed email from Appendix 23

		• Added Appendix 25
**4.3**	April 07, 2022	• Updated sample size to the final number of enrolled patients<br>• Added dual enrollment with CSP2005 that has been approved by the Study PIs and Coordinating Centers<br>• Clarified how data may be used for the analysis of secondary and tertiary objectives
**4.4**	May 09, 2022	• Added dual enrollment with CSP2026 that has been approved by both study PIs and Coordinating Centers

*Please note that the protocol version initially approved by the VA Central IRB was version 2.1

<table>
<tr><td rowspan="2">MAVERIC Study Specific Documents</td><td>DATE OF ISSUE</td><td colspan="2">DOCUMENT DESCRIPTION</td></tr>
<tr><td>8/29/2017</td><td colspan="2">This document describes the statistical analysis plan for the CSP597.</td></tr>
<tr><td colspan="2">AUTHORS: Robert Lew, PhD
Sarah Leatherman, PhD
Cynthia Hau, MPH</td><td colspan="2">SUBITTED BY:</td></tr>
<tr><td colspan="4">DOCUMENTS/ RECORD HISTORY:</td></tr>
<tr><td>DATE</td><td>VERSION</td><td colspan="2">RECORD HISTORY</td></tr>
<tr><td>12/14/2016

8/29/2017</td><td>Initial Version for review at DMC meeting

Version 1.0</td><td colspan="2">

Finalized comments generated after DMC meeting</td></tr>
<tr><td colspan="4">AUTHORIZATION SIGNAURES</td></tr>
<tr><td>NAME</td><td>FUNCTION TITLE</td><td>SIGNATURE</td><td>DATE</td></tr>
<tr><td>Robert Lew, PhD</td><td>Biostatistician</td><td>[illegible]</td><td>[illegible]</td></tr>
<tr><td></td><td></td><td></td><td></td></tr>
</table>

Table of Contents

1. Introduction 5
 1.1 Overview of the Study Design and Objectives 5
2. Investigational Plan 6
 2.1 Description of the Study Population 6
  2.1.1 Inclusion Criteria 6
  2.1.2 Exclusion Criteria 6
 2.2 Description of the Intervention Strategy 7
  2.2.1 Randomization 7
  2.2.2 Study Intervention 7
  2.2.3 Follow-Up Assessment 7
3. Statistical Methods 8
 3.1 Primary Data Analysis 8
  3.1.1 Primary Objective 8
  3.1.2 Primary Endpoint 8
  3.1.3 Primary Hypothesis 8
  3.1.4 Statistical Methods for Primary Data Analysis 8
 3.2 Secondary Data Analysis 9
  3.2.1 Secondary Objectives 9
  3.2.2 Secondary Endpoints 9
  3.2.3 Statistical Methods for Secondary Data Analyses 10
 3.3 Tertiary Data Analysis 10
  3.3.1 Tertiary Objective 10
  3.3.2 Tertiary Endpoints 10
  3.3.3 Statistical Methods for Tertiary Data Analysis 11
 3.4 Exploratory Data Analysis 11
  3.4.1 Exploratory Objective 11
  3.4.2 Statistical Methods for Exploratory Data Analysis 11
4. Sample Size and Power 13
 4.1 Interim Analysis 13
 4.2 Futility Analysis 13
5. General Considerations 14
 5.1 Definition of Intention to Treat Sample 14

5.2 Definition of Per-Protocol Sample 14
5.3 Missing and Miscoded Data 14
5.4 Baseline Characteristics 15
5.4.1 Demographics 15
5.4.2 Clinical factors 15
5.5 Safety Evaluation and Reporting 15
5.4 Outcome Adjudication 16
6. Data Monitoring Committee Report 17
6.1 Analytic Sample for DMC Report. 17
6.2 Algorithm 17
6.3 Outline of DMC Report 17
6.3.1 Section A: Subject Disposition 17
6.3.2 Section B: Baseline and Follow-Up Measures 17
6.3.3 Section C: Outcome Measures 18
6.3.4 Section D: Safety Assessment 18

4487 ## Abbreviations

ACME	Automated Classification of Medical Entitles
ADD	Average Daily Dose
BMI	Body Mass Index
CDW	Corporate Data Warehouse
ED	Erectile Dysfunction
CMS	Centers for Medicare & Medicaid Services
CPRS	Computerized Patient Record System
CSP	Cooperative Studies Program
CSPCC	Cooperative Studies Program Coordinating Center
CV	Cardiovascular
CVD	Cardiovascular Disease
DCP	Diuretic Comparison Project
DMC	Data Monitoring Committee
ED	Erectile Dysfunction
eGFR	estimated Glomerular Filtration Rate
EMR	Electronic Medical Record
GEE	Generalized Estimating Equation
ICD	International classification of disease
ITT	Intention To Treat
MI	myocardial infarction
MPR	Medication Possession Ratio
MSM	Multistage Model
PCP	Primary Care Provider
POC	Point-Of-Care
SAP	Statistical Analysis Plan
SBP	Systolic Blood Pressure
SOP	Standard Operating Procedure
VA	Veterans Affairs
VHA	Veterans Health Administration

1. Introduction

This document outlines the statistical methods for the analysis of the data collected in the Department of Veterans Affairs (VA) Cooperative Studies Program (CSP) study #597 entitled "Diuretic Comparison Project {DCP)". The purpose of this document is to provide guidelines from which the analysis will proceed.

1.1 Overview of the Study Design and Objectives

The present study is an open-label, blinded-endpoint, multicenter, prospective randomized two-arm controlled clinical trial. This trial is designed to compare the effects of two thiazide-type diuretics, hydrochlorothiazide and chlorthalidone, on cardiovascular {CV) events and non-cancer death. A randomized trial comparing the effectiveness of the two drugs has never been conducted, primarily for reasons of cost. Consequently, a less-expensive study design {termed a "point-of-care {POC)" trial) is utilized to answer the question of whether chlorthalidone is more effective than hydrochlorothiazide at preventing CV events and non-cancer death among older patients with hypertension.

This study aims to enroll 13,500 patients over 3 years at the 50 Veterans Affairs {VA) medical centers. The participants will berandomized with equal probability into one of the treatment arms. Based on time of enrollment, participants can be followed for a maximum of 4.5 years. Assuming a uniform rate of enrollment over time (90 patients per medical center each year), the average follow-up time will be approximately 3 years among all participants.

2. Investigational Plan

2.1 Description of the Study Population

Patients who satisfy the inclusion and exclusion criteria are considered eligible to be enrolled in this study.

2.1.1 Inclusion Criteria

Patients who meet the following criteria are considered eligible:

I. Over 65 years of age

II. Receiving hydrochlorothiazide from the VA pharmacy at a daily dose of 25 or 50 mg

III. Have a most recent systolic blood pressure (SBP) 120 mmHg, with no SBP <120 mmHg recorded in CPRS over the past 90 days

2.1.2 Exclusion Criteria

Patients with any of the following conditions are excluded from enrollment:

I. Impaired decision-makingcapacity rendering the patient unable to provide informed consent. (Indicated in medical chart or determined by the primary care provider (PCP))

II. Death expected within 6 months (inferred by PCP permission to randomize)

III. Potassium <3.1or <3.5 (if on digoxin) meq/L over the past 90 days

IV. Sodium <130 meq/L over the past 90 days

V. Enrolled in Medicare Part C (extracted from administrative data or obtained from patients through consent phone call)

2.2 Description of the Intervention Strategy

2.2.1 Randomization

Participants will be randomized with a 1:1 allocation ratio to:

I. Continue receiving hydrochlorothiazide at current daily dose (25 or SO mg), or
II. Switch to chlorthalidone at half dose (12.5 or 25 mg, respectively)

Both stratification and blocking will be employed to control for potential imbalance in randomization. The randomization scheme will be stratified by participating medical centers to account for possible regional differences in clinical practice, and the treatment allocation will be performed within blocks of size 6.

2.2.2 Study Intervention

We will obtain informed consent from patient for study participation. Prior to the start of the intervention, we will obtain approval from his/her associated PCP for randomizing the particular patient. Participants of this study will be randomly allocated to either the hydrochlorothiazide or chlorthalidone arm. The administration of the allocated treatment will be managed by his/her PCP. There are no study-specific clinic visits or assessments. During the entire study period, participants will be monitored by their health care providers through usual clinical care. Data collection of this study will be performed by extracting relevant study data from electronic medical records. A series of pre-defined_algorithms will be developed to identify study outcomes and safety events. The overall compliance of study medications will be determined indirectly using medication possession ratio (MPR) and average daily dose (ADD) based on electronic pharmacy records.

2.2.3 Follow-Up Assessment

Participants will be followed passively through the VA electronic medical record (EMR) system. The pre-defined extraction algorithms will be performed and collect relevant follow-up data from the VHA Corporate Data Warehouse (CDW), Centers for Medicare & Medicaid Service {CMS) database, and the National Death Index.

Assuming a uniform rate of enrollment over time, the first patient entered will have 4.5 years of follow-up and the last patient entered will have 1.5 years of follow-up, yielding an average follow-up time of 3 years on 13,500 subjects.

3. Statistical Methods

3.1 Primary Data Analysis

3.1.1 Primary Objective

The primary objective is to determine whether chlorthalidone is superior to hydrochlorothiazide for the prevention of CV events and non-cancer death over time.

3.1.2 Primary Endpoint

The primary endpoint is time to a composite outcome involving CV events of interest and non-cancer death. The CV events of interest are defined as hospitalization for stroke, myocardial infarction (Ml), urgent coronary revascularization due to unstable angina, and acute decompensated heart failure. Time to event will be measured as time from study enrollment to the first occurrence of the composite outcome.

3.1.3 Primary Hypothesis

We posit a 4.5 year event rate of 13.5% in the hydrochlorothiazide arm and a 17.5% reduction of CVD events in the chlorthalidone arm to inform our primary hypothesis stated below:

H_0: The 4.5 year.event rate will be 13.5% (or 578 primary events) in both treatment arms

H_1: The 4.5 year event rate will be 11.1% (or 477 primary events) in the chlorthalidone arm

In theory, if the primary events occurred as projected, the relative percent change will be 17.5%((578-477)/ 578 *100%}. The hazard ratio (chlorthalidone hazard rate/ hydrochlorothiazide hazard rate) will be approximately 0.81, which will be midway between the odds ratio of 0.80 (11.1(100-13.5)/13.5(100-11.l}) and the risk ratio of 0.82 (11.1/13.5}.

3.1.4 Statistical Methods for Primary Data Analysis

The effect of treatment on the primary study outcome will be assessed by means of a two-sided log-rank test and Cox proportional hazards models based on intention-to-treat (ITT) principles. Both unadjusted and adjusted models will be used. The adjusted Cox model will account for baseline characteristics such as demographics (e.g., age, sex) and clinical factors (e.g., blood pressure, medications, history of disease, and body mass index (BMI)). A time-treatment interaction term will also be included to test the proportional hazards assumption. The final study results will present both unadjusted

and adjusted hazard rates and corresponding hazard ratios with 95% confidence intervals.

3.2 Secondary Data Analysis

3.2.1 Secondary Objectives

The secondary objectives are to compare the treatment effects 1) across pre-specified subgroups, 2) on individual components of the primary outcomes, and 3) on additional outcomes of interest.

Differential treatment effects will be evaluated in the following baseline characteristics:

I. gender
II. age (dichotomized at the median)
III. baseline SBP (dichotomized a the median)
IV. history of MI or stroke
V. black race vs. not
VI. diabetes vs. not
VII. eGFR <60 ml/min/1.73m^2
VIII. good compliance (medication possession ratio 80%) with hydrochlorothiazide over the year before randomization

3.2.2 Secondary Endpoints

Individual components of the primary outcome will be examined:

I. stroke
II. **MI**
III. urgent coronary revascularization due to unstable angina
IV. hospitalization for acute decompensated heart failure
V. non-cancer death

Treatment effects will be evaluated for additional outcomes below:

I. all-cause mortality
II. the composite outcome substituting all deaths for non-cancer deaths

III. "possible vascular deaths" defined as all deaths caused by vascular diseases, diabetes, external causes, and unknown causes

IV. the composite outcome substituting "possible vascular deaths" for non-cancer deaths

V. any revascularization of an artery

VI. erectile dysfunction (ED), defined as first prescription of PDES inhibitor or referral for ED

3.2.3 Statistical Methods for Secondary Data Analyses

Cox regression modeling will be used to explore variation in treatment efficacy across the pre-specified subgroups. The components of the primary outcomes will be examined individually using log-rank and Cox proportional hazards models. Both unadjusted and adjusted analyses will be performed. Covariate adjustment will be similar to that used in the primary data analysis.

3.3 Tertiary Data Analysis

3.3.1 Tertiary Objective

The tertiary objective is to examine the occurrence of expected adverse events (defined as common events related to diuretics), and their associations with the assigned treatment regimens.

3.3.2 Tertiary Endpoints

The expected adverse events of this study are:

I. hospitalization for primary diagnosis of hypokalemia, hyponatremia, or renal failure

II. renal failure, defined as dialysis, vascular access for dialysis, or renal transplant

III. other recorded hypokalemia (<3.5 meq/L) or hyponatremia (<130 meq/L)

IV. new diabetes, defined as first use of medication for diabetes

V. acute gout episodes

VI. new allergic reaction to thiazide-type diuretics

3.3.3 Statistical Methods for Tertiary Data Analysis

The expected adverse event will be examined individually using log-rank and Cox proportional hazards models. Both unadjusted and adjusted Cox models will be performed. The adjustment for baseline covariates will be similar to that used in the primary data analysis.

3.4 Exploratory Data Analysis

3.4.1 Exploratory Objective

The ITT analyses take no account of post-baseline changes in study medications. Thus, exploratory analyses may be performed to examine the primary, secondary, and tertiary endpoints in a per-protocol subset, which defined as participants who persistently receive the allocated medication prescriptions throughout the duration of the study.

Other exploratory analyses may be performed to evaluate the treatment efficacy with considerations of protocol deviations and time-varying repeated measures, as well as treatment interruptions due to hospitalization and other clinical factors. We may also explore the between-group difference in SBP, which will be measured repeatedly over the disease-free intervals (free of component events).

3.4.2 Statistical Methods for Exploratory Data Analysis

The per-protocol analysis will be performed following statistical procedures used in the ITT cohort. Other exploratory analyses may be conducted using a generalized estimating equation (GEE) to account for var.ying time intervals and treatment interruptions, with potential modeling of time-dependent effects of protocol deviations. We may also explore censoring patterns such as models that assume not missing-at-random using a Bayesian approach to the pattern mixture model (Missing Data in Longitudinal Studies Chapman and Hall Boca Raton 2008). In addition, a change in medication may alter the subsequent risk of a cardiovascular event. Therefore, we may add time-dependent covariates to the Cox model, such as binary indicators of medication switching (from chlorthalidone to hydrochlorothiazide, or vice versa). To directly estimate subsequent risk, we may use a multistage model (MSM) to assess the hazard rate of a major cardiovascular event after a switch. MSM models extend the time-to-first-event models to second, third, and more events. This extension of the Cox model allows direct estimation of the hazard rate associated with any transition adjusted for previous history and baseline characteristics. Within MSM, we may carry out a competing risks analysis to assess the effect of cancer deaths.

In addition, a mixed effects model for repeated measures may be used to test whether:

1) SBPincreases/decreases over time among participants receiving chlorthalidone, 2) SBP increases/decreases over time among participants receivingydrochlorothiazide,

and 3) SBP is statistically significant different between study arms. The model will allow for irregular time intervals and a random effect will be included for both intercept and linear time.

4. Sample Size and Power

The annual primary outcome occurrence rate is projected to be 3% (578 events over 4.5 years) in the hydrochlorothiazide arm. With the goal of enrolling 13,500 patients (6,750 in each study arm for an overall type-I error of 5%), the two-sided test will have a 90% power to detect a 17.5% reduction in CV events among participants receiving chlorthalidone. Thus, this study will have a 90% power to detect a hazard ratio of less than 0.82 or greater than 1.22 in the chlorthalidone arm. However, if the annual occurrence rate is between 2% and 2.5% (rather than the posited 3%), the study will have a power of 75% to 84%.

4.1 Interim Analysis

This study will have one interim analysis, which will be performed when the 500th primary outcome event occurs (approximately 3.5 years after initiation of enrollment). Assuming a uniform rate of enrollment over time, the first patient entered will potentially have 3.5 years of follow-up and the last patient entered will potentially have 0.5 years of follow-up. Thus, we will have an average follow-up time of approximately 2 years on 13,500 subjects.

Using the O-Brien Fleming procedure, this interim analysis will have a type I error of 0.1%, which negligibly decreases the overall type I error and has virtually no effect on the power to show that chlorthalidone is different from hydrochlorothiazide. Given the potential inflation factor, the increase in sample size of 1.001will result in 14 more subjects per arm. We will confer with the Data Monitoring Committee (DMC) members and the program leadership for potential stopping guidelines based on findings from the interim analysis.

4.2 Futility Analysis

If requested by the DMC, a futility analysis that includes conditional probability estimation will be conducted to determine the probability of observing a significant result assuming the distribution of future event rates from additional data from the second half of the study follow three scenarios. These scenarios are:

1) No-change - future event rates are the same as the currently observed event rates,
2) Expected - future event rates are as proposed in the protocol, and
3) Extreme - all new events at the currently observed event rate are in the control group.

5. General Considerations

5.1 Definition of Intention to Treat Sample

All consented and randomized subjects will be accounted for and reported in the CONSORT diagram. However, only those consented subjects for whom a randomized drug order is entered will be considered as ITT subjects and included in the DMC reports and primary efficacy analysis. An analytic sample data file consisting of only the ITT subjects will becreated and maintained throughout the study period.

5.2 Definition of Per-Protocol Sample

Participants categorized as protocol compliant will make up the per-protocol subset. The overall compliance to the randomly assigned medication will bemeasured over the entire follow-up period using MPR and ADD defined as follows:

$$MpR = \frac{\text{Days of chlorthalidone or hydrochlorothiazide supply between the date of randomization and the study end date}}{(\text{Total number of days between the first and last refill date}) + \text{days of supply from the last refill}}$$

$$ADD = \frac{\text{Total cumulative dose of chlorthalidone or hydrochlorothiazide}}{\text{available days of supply}}$$

5.3 Missing and Miscoded Data

A related issue is the possibility that patients who suffer a cardiac or non-cardiac event during the study will have the wrong ICDl0 code assigned despite review of the electronic patient record by cardiologists in our study. If this appears to be a major issue we will apply Carroll Ruppert and Stefanski's methods as implemented in the r_langauge package DECON (2013). While this will not correct any errors it will increase the standard errors and thereby assess the robustness of our conclusions.

Changes in medication may also prove to be a major issue of missing data. Subjects in this study will not have frequent clinic visits wherein our staff asks them about their current medications. Instead we will passively detect changes by review of electronic charts including pharmacy data. A systematic change in medication, if not detected,

would bias study results. Thus, we will intensively review electronic data for any evidence of such changes. This vigilance is more a matter of study protocol than statistical adjustment.

5.4 Baseline Characteristics

With the use of all available data stored in the CDW, baseline measurements will be recorded as the closest date before randomization. The baseline measurement will be evaluated among the entire study cohort, between study arms, and stratified by enrollment sites. The number of observations, mean, median, standard deviation, minimum, and maximum will be calculated for continuous variables. The categorical results will be reported as frequencies and percentages.

Distribution of continuous variables and proportions of categorical variables will be tabulated by intervention group, and t-test and chi-square tests will be performed to evaluate if these variables are balanced across the two intervention groups.

5.4.1 Demographics

Baseline demographics including age, sex, race, ethnicity, marital status, military service, height, weight, and BMI will be determined before the start of the study intervention.

5.4.2 Clinical factors

Critical clinical factors such as medical history, comorbidities, medication history, vital signs, and key laboratory values will also be extracted.

5.5 Safety Evaluation and Reporting

The CSPCC staff will collect safety data from the medical record from the time of consent through the end of the study period. If the subject withdraws from the study prior to study end, collection of safety data will cease on the date of withdrawal.

Pre-defined safety events of interest will beidentified through data extraction from the VA CDW and CMS database. In brief, participants' health care providers will identify, monitor, and treat (as necessary) adverse events that occur during the course of the study. Informatics staff at the MAVERIC CSPCC will extract EMR data routinely with pre-defined algorithms including, but not limited to, International Classification of Disease (ICD) 10 codes, Current Procedure Terminology (CPT) codes, medication names, and laboratory test name with critical values. In addition to data culled from the EMR, the trial will allow for spontaneous reporting of events by study participants. There are no unanticipated safety events for this study. However, participants will begiven a study-specific information sheet with relevant contact information for communicating safety concerns with the study team.

All safety data will bereported with aggregated data tables detailing the frequencies of these events by treatment arms. The expected adverse events will b_e reported to in the DMC report as secondary and tertiary outcomes.

5.4 Outcome Adjudication

In cases where the outcome diagnosis is not clear based on the electronic adjudication, we will resort to manual adjudication to determine the validity of the diagnosis. Please refer to the study protocol for more details regarding the process of outcome adjudication.

6. Data Monitoring Committee Report

Data and study progress will be monitored by the study executive committee and by the DMC. The DMC will review the study progress and safety semi-annually with additional meetings and communications as needed.

6.1 Analytic Sample for DMC Report

All subjects randomized more than four weeks prior to the DMC meeting date will be included in the analytic cohort for the upcoming DMC report.

6.2 Algorithm

Shell tables with annotated algorithms for creating aggregated tables in the DMC report are kept securely on the CSP597 SharePoint site and/or study data storage network.

Only authorized study personnel will have access to the secured SharePoint site/data storage network.

6.3 Outline of DMC Report

The DMC report is divided into four sections to cover subject disposition, baseline & follow-up Measures, outcome measures, and safety events. Following is the list of tables/figures to be included within each of these sections. Revision to this list, if any, will be discussed at the first DMC meeting.

6.3.1 Section A: Subject Disposition

Table Al. Consort Diagram for CSP597

Table A2. Enrollment by Sites

Table A3. Protocol Deviation

6.3.2 Section B: Baseline and Follow-Up Measures

Table Bl. Baseline information

Table B2. Medical History

Table B3. Compliance to study medication

Figure B4. Current prescription status of study medication

Table BS. Systolic blood pressure and other use of antihypertensive Agents

6.3.3 Section C: Outcome Measures

Table Cl. Occurrence of Study Outcomes

6.3.4 Section D: Safety Assessment

Table D1. Expected Safety Events

Table D2. List of Unanticipated Serious Adverse Events
