# Supplementary material for: Chlorthalidone vs Hydrochlorothiazide and Kidney Outcomes in Patients With Hypertension: A Secondary Analysis of a Randomized Clinical Trial
Source: JAMA Netw Open. 2024 Dec 10;7(12):e2449576. doi: 10.1001/jamanetworkopen.2024.49576 (PMC11632543; doi:10.1001/jamanetworkopen.2024.49576)
Supplement: Supplement 2. — eFigure 1. Consort Diagram of Patient Flow eTable 1. CPT Codes Used to Define Dialysis Procedures eTable 2. Demographic and Baseline Information by Treatment and eGFR Level eFigure 2. Primary Outcome: Time to Doubling of Serum Creatinine, Terminal eGFR<15 mL/min, or Dialysis Initiation eFigure 3. Exploratory Outcome: Time to a 40% Reduction in eGFR, Terminal eGFR<15 mL/min, or Dialysis Initiation eTable 3. Use of Selected Medications Over Time eFigure 4. Adjusted Subgroup Analysis: Time to Doubling of Serum Creatinine, Terminal eGFR<15 mL/min/1.73m2, or Dialysis Initiation eFigure 5. Unadjusted Subgroup Analysis: Time to >40% Reduction in eGFR, Terminal eGFR<15 mL/min/1.73m2, or Dialysis Initiation eTable 4. Safety Outcomes by Treatment and eGFR Level [file jamanetwopen-e2449576-s002.pdf]

Supplementary Online Content

Ishani A, Hau C, Raju S, et al. Chlorthalidone vs hydrochlorothiazide and kidney outcomes in patients with hypertension: a secondary analysis of a randomized clinical trial. *JAMA Netw Open*. 2024;7(12):e2449576. doi:10.1001/jamanetworkopen.2024.49576

eFigure 1. Consort Diagram of Patient Flow

eTable 1. CPT Codes Used to Define Dialysis Procedures

eTable 2. Demographic and Baseline Information by Treatment and eGFR Level[a]

eFigure 2. Primary Outcome: Time to Doubling of Serum Creatinine, Terminal eGFR < 15 mL/min, or Dialysis Initiation

eFigure 3. Exploratory Outcome: Time to a 40% Reduction in eGFR, Terminal eGFR <15 mL/min, or Dialysis Initiation

eTable 3. Use of Selected Medications Over Time

eFigure 4. Adjusted Subgroup Analysis: Time to Doubling of Serum Creatinine, Terminal eGFR <15 mL/min/1.73m^2, or Dialysis Initiation

eFigure 5. Unadjusted Subgroup Analysis: Time to > 40% Reduction in eGFR, Terminal eGFR < 15 mL/min/1.73m^2, or Dialysis Initiation

eTable 4. Safety Outcomes by Treatment and eGFR Level

This supplementary material has been provided by the authors to give readers additional information about their work.

© 2024 Ishani A et al. *JAMA Network Open.*

eFigure 1. Consort Diagram of Patient Flow

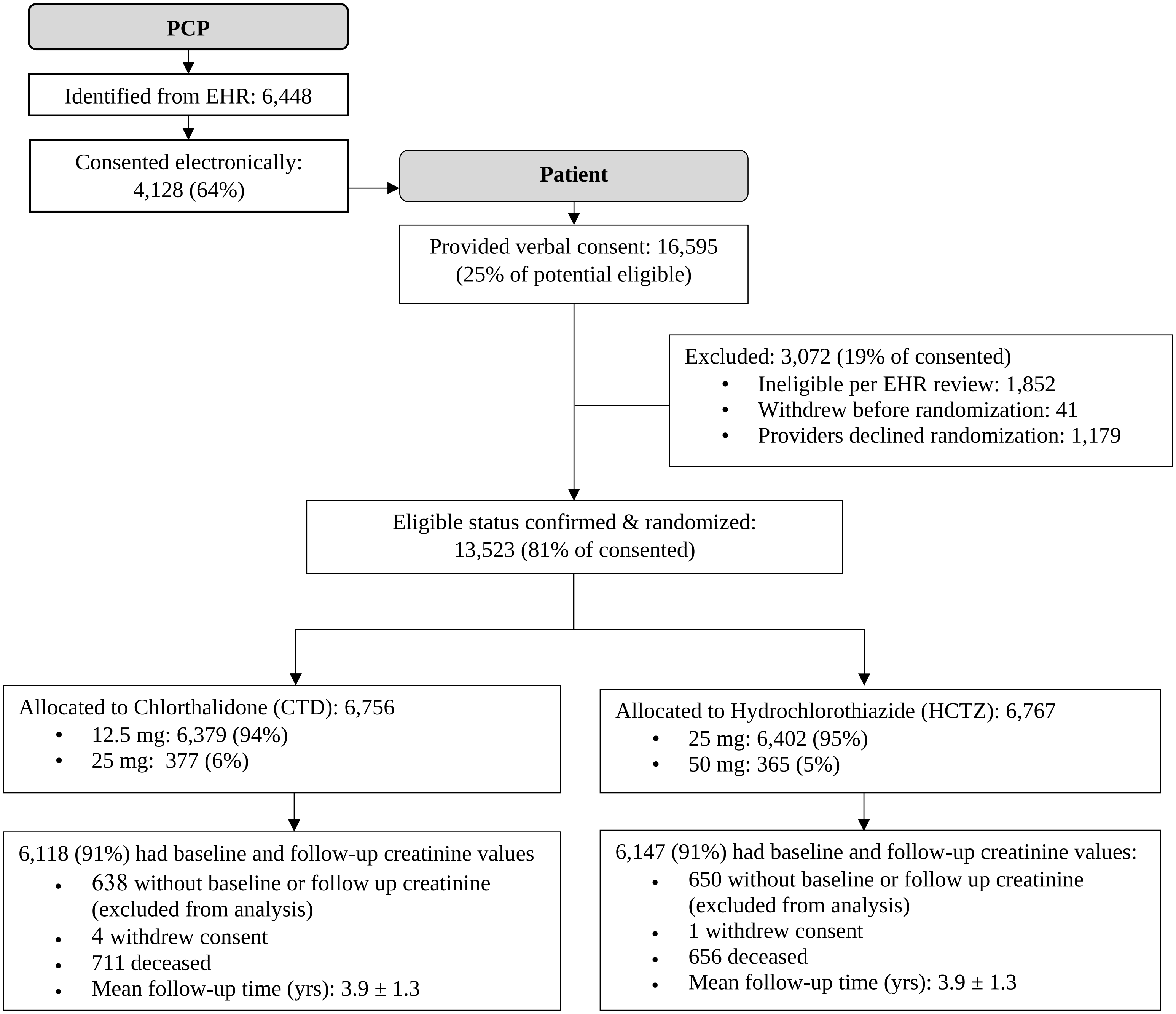

© 2024 Ishani A et al. *JAMA Network Open.*

eTable 1. CPT Codes Used to Define Dialysis Procedures

**CPT Codes**	
**Dialysis**	90918, 90919, 90920, 90921, 90922, 90923, 90924, 90925, 90935, 90937, 90939, 90940, 90941, 90942, 90943, 90944, 90945, 90947, 90951, 90952, 90953, 90954, 90955, 90956, 90957, 90958, 90959, 90960, 90961, 90962, 90963, 90964, 90965, 90966, 90967, 90968, 90969, 90970, 90976, 90977, 90978, 90979, 90982, 90983, 90984, 90985, 90988, 90989, 90990, 90991, 90992, 90993, 90994, 90995, 90996, 90997, 90998, 90999

© 2024 Ishani A et al. *JAMA Network Open.*

eTable 2. Demographic and Baseline Information by Treatment and eGFR Level[a]

	Baseline eGFR >=60 (N = 9,038)		Baseline eGFR <60 (N = 3,227)	
	**CTD (n = 4,520)**	**HCTZ (n = 4,518)**	**CTD (n=1,598)**	**HCTZ (n=1,629)**
Age, yrs – median (IQR)	71 (68 – 74)	71 (68 – 74)	73 (70 – 77)	73 (70 -77)
Body Mass Index, kg/m^2 - median (IQR)	31.2 (27.8 – 35.1)	31.3 (27.9 – 35.3)	30.9 (27.7- 34.8)	31.0 (27.9 – 35.0)
Sex				
Female	149 (3.3)	139 (3.1)	49 (3.1)	52 (3.2)
Male	4,371 (96.7)	4,379 (96.9)	1,549 (96.9)	1,577 (96.8)
Race				
Black	704 (15.6)	685 (15.2)	211 (13.2)	237 (14.5)
White	3501 (77.5)	3,514 (77.8)	1,244 (77.8)	1,259 (77.3)
Other[b]	94 (2.1)	96 (2.1)	50 (3.1)	46 (2.8)
Unknown due to missing data	221 (4.9)	223 (4.9)	93 (5.8)	87 (5.3)
Ethnicity				
Hispanic/Latino	164 (3.6)	185 (4.1)	55 (3.4)	60 (3.7)
Not Hispanic/Latino	4,199 (92.9)	4,190 (92.7)	1,482 (92.7)	1,503 (92.3)
Unknown due to missing data	157 (3.5)	143 (3.2)	61 (3.8)	66 (4.1)
Resided in rural areas[c]	2,043 (45.2)	2,074 (45.9)	735 (46.0)	709 (43.5)
Current smoker	1,038 (23.0)	959 (21.2)	347 (21.7)	342 (21.0)
Medical History				
Diabetes	1,865 (41.3)	1,926 (42.6)	898 (56.2)	936 (57.5)
Heart failure	312 (6.9)	295 (6.5)	174 (11.0)	192 (11.8)
Myocardial infarction	146 (3.2)	152 (3.4)	68 (4.3)	80 (4.9)
Stroke	322 (7.1)	294 (6.5)	172 (10.8)	151 (9.3)
Hydrochlorothiazide (HCTZ) 25 mg	4,279 (94.6)	4,266 (94.4)	1,508 (94.4)	1,544 (94.8)
Systolic BP, mm Hg - median (IQR)	139 (131 – 152)	139 (130 – 152)	139 (130 – 152)	139 (130 – 154)
Serum Creatinine, mg/dL - median (IQR)	1.0 (0.9 – 1.1)	1.0 (0.9 – 1.1)	1.4 (1.3 – 1.6)	1.4 (1.3 – 1.6)
Serum Potassium, mg/dL - median (IQR)	4.0 (3.8 – 4.3)	4.0 (3.8 – 4.3)	4.1 (3.9 – 4.5)	4.1 (3.9 – 4.4)
eGFR, $ml/min/1.73m^2$ - median (IQR)	77.5 (68.6 – 89.2)	77.2 (68.3 – 87.3)	50.0 (42.3 – 55.0)	49.8 (42.1 – 55.2)
Antihypertensive drug use				
HCTZ alone	644 (14.2)	633 (14.0)	148 (9.3)	127 (7.8)
HCTZ + 1 additional BP med	1,606 (35.5)	1,584 (35.1)	494 (30.9)	482 (29.6)
HCTZ + 2 additional BP meds	1,454 (32.2)	1,464 (32.4)	535 (33.5)	562 (34.5)
HCTZ + 3 additional BP meds	668 (14.8)	666 (14.7)	316 (19.8)	339 (20.8)
HCTZ + 4 additional BP meds	148 (3.3)	171 (3.8)	105 (6.6)	119 (7.3)
Other drug use				
ACE inhibitor or ARB	2,841 (62.9)	2,844 (62.9)	1,133 (70.9)	1,191 (73.1)
Loop diuretic	73 (1.6)	83 (1.8)	58 (3.6)	74 (4.5)
SGLT-2 inhibitor	110 (2.4)	109 (2.4)	51 (3.2)	61 (3.7)
Spironolactone or Eplerenone	581 (12.9)	601 (13.3)	188 (11.8)	183 (11.2)

a Data are presented as number(percentage) of patients unless otherwise indicated.

© 2024 Ishani A et al. *JAMA Network Open.*

b Included American Indian, Asian, Hawaiian, Pacific Islander, and multiracial.
c Based on Place of residence was defined according to the Veterans Affairs urban, rural, or highly rural classification system.

© 2024 Ishani A et al. *JAMA Network Open.*

eFigure 2. Primary Outcome: Time to Doubling of Serum Creatinine, Terminal eGFR < 15 mL/min, or Dialysis Initiation

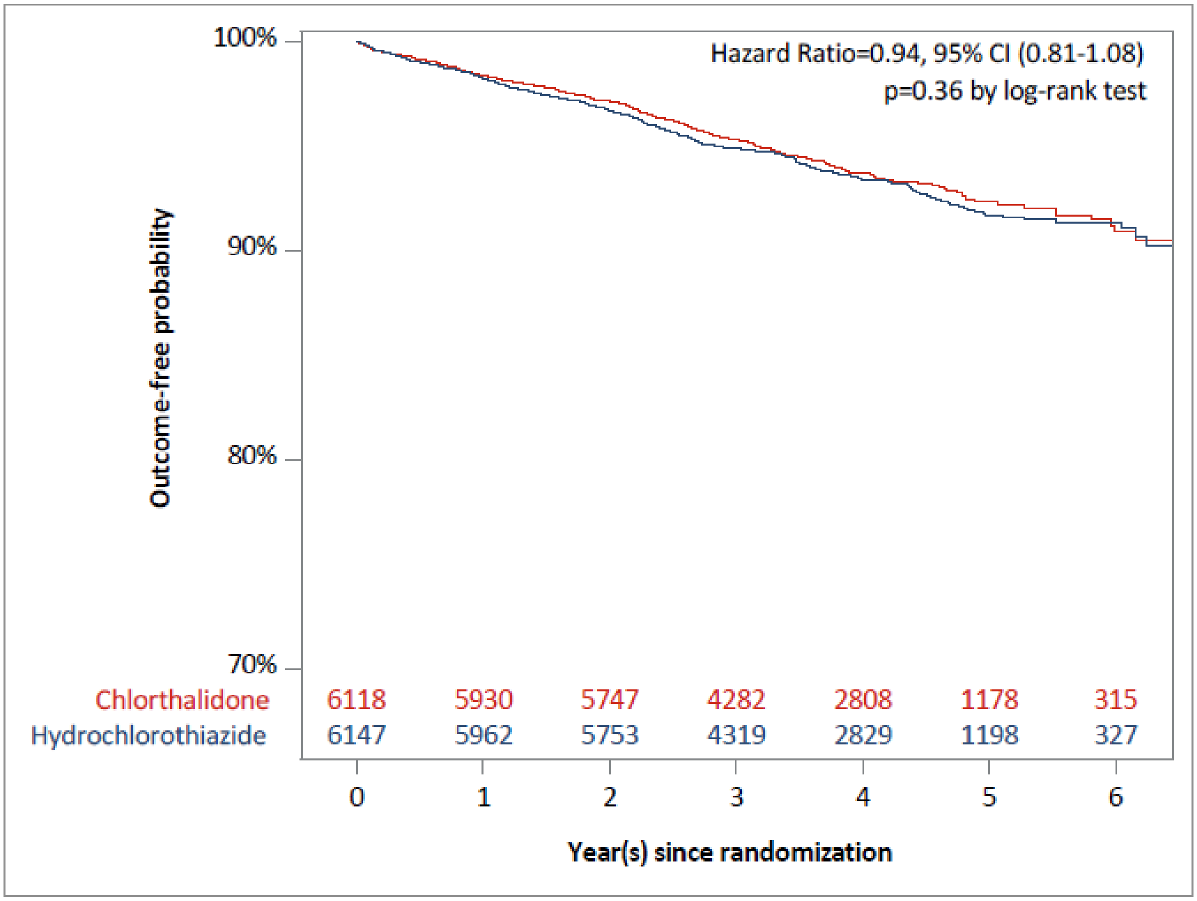

© 2024 Ishani A et al. *JAMA Network Open.*

eFigure 3. Exploratory Outcome: Time to a 40% Reduction in eGFR, Terminal eGFR <15 mL/min, or Dialysis Initiation

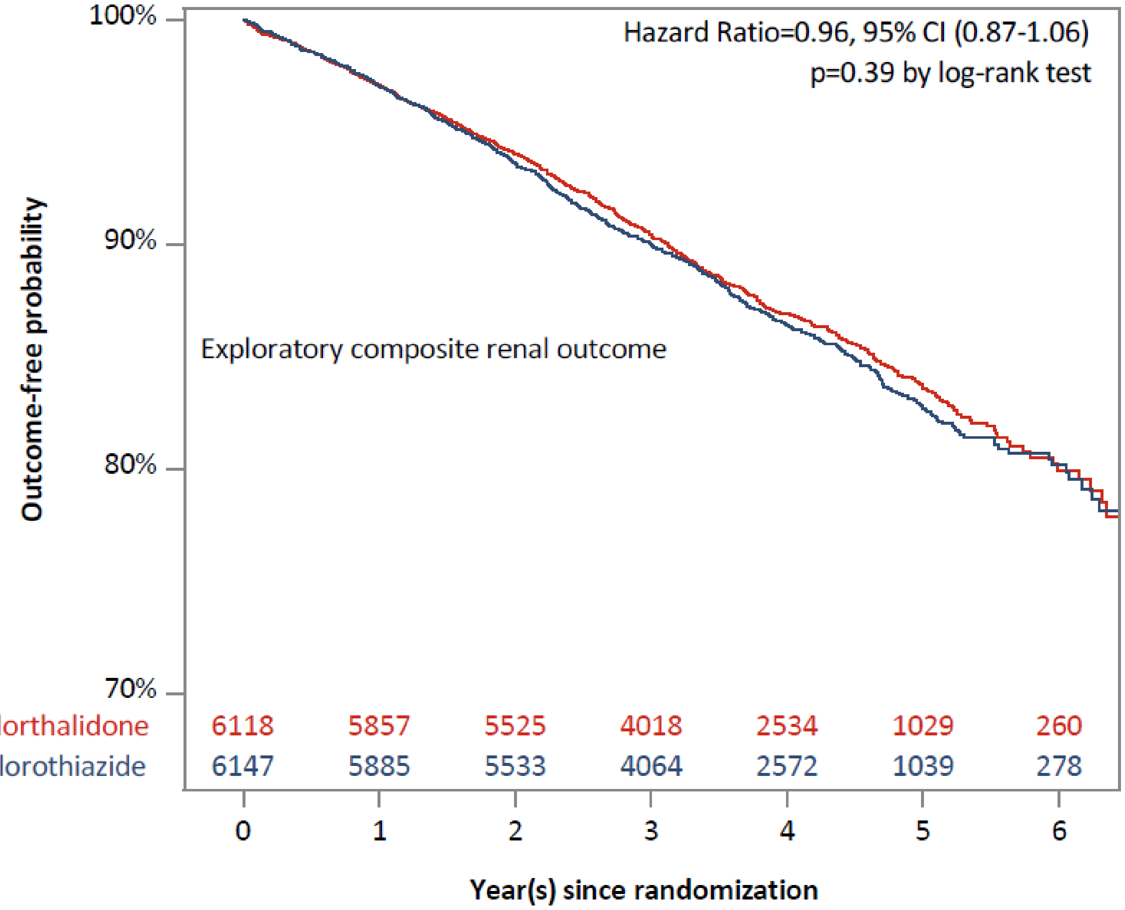

© 2024 Ishani A et al. *JAMA Network Open.*

eTable 3. Use of Selected Medications Over Time

Baseline eGFR <60 (N=3,227)	Baseline		6 months		12 months		18 months		24 months	
	CTD (n=4,520)	HCTZ (n=4,518)	CTD (n=4,503)	HCTZ (n=4,501)	CTD (N=4,014)	HCTZ (n=4,048)	CTD (n=3,352)	HCTZ (n=3,366)	CTD (n=2,706)	HCTZ (n=2,724)
ACE	1,604 (35.5)	1,650 (36.5)	1,555 (34.5)	1,629 (36.2)	1,377 (34.3)	1,448 (35.8)	1,121 (33.4)	1,196 (35.5)	886 (32.7)	934 (34.3)
ARB	1,256 (27.8)	1,209 (26.8)	1,247 (27.7)	1,233 (27.4)	1,119 (27.9)	1,105 (27.3)	949 (28.3)	907 (26.9)	766 (28.3)	728 (26.7)
ACE or ARB	2,841 (62.9)	2,844 (62.9)	2,788 (61.9)	2,844 (63.2)	2,481 (61.8)	2,543 (62.8)	2,052 (61.2)	2,093 (62.2)	1,645 (60.8)	1,655 (60.8)
SGLT2i	110 (2.4)	109 (2.4)	163 (3.6)	160 (3.6)	174 (4.3)	167 (4.1)	161 (4.8)	139 (4.1)	140 (5.2)	138 (5.1)
MRA	581 (12.9)	601 (13.3)	643 (14.3)	650 (14.4)	645 (16.1)	617 (15.2)	542 (16.2)	522 (15.5)	475 (17.6)	446 (16.4)
Loop diuretics	73 (1.6)	83 (1.8)	95 (2.1)	129 (2.9)	130 (3.2)	171 (4.2)	140 (4.2)	176 (5.2)	135 (5.0)	159 (5.8)

Baseline eGFR>=60 (N=9,038)	Baseline		6 months		12 months		18 months		24 months	
	CTD (n=1,598)	HCTZ (n=1,629)	CTD (n=1,591)	HCTZ (n=1,620)	CTD (N=1,412)	HCTZ (n=1,405)	CTD (n=1,146)	HCTZ (n=1,160)	CTD (n=888)	HCTZ (n=885)
ACE	662 (41.4)	675 (41.4)	648 (40.7)	648 (40.0)	577 (40.9)	554 (39.4)	452 (39.4)	447 (38.5)	346 (39.0)	331 (37.4)
ARB	472 (29.5)	525 (26.8)	473 (29.7)	528 (27.4)	414 (29.3)	449 (27.3)	346 (30.2)	377 (26.9)	273 (30.7)	280 (26.7)
ACE or ARB	1,133 (70.9)	1,191 (73.1)	1,115 (70.1)	1,172 (72.3)	985 (69.8)	1,001 (71.2)	791 (69.0)	822 (70.9)	617 (69.5)	63 (7.1)
SGLT2i	51 (3.2)	61 (3.7)	79 (5.0)	97 (6.0)	79 (5.6)	91 (6.5)	66 (5.8)	84 (7.2)	61 (6.9)	107 (12.1)
MRA	188 (11.8)	183 (11.2)	218 (13.7)	192 (11.9)	201 (14.2)	173 (12.3)	156 (13.6)	163 (14.1)	126 (14.2)	126 (14.2)
Loop diuretics	58 (3.6)	74 (4.5)	88 (5.5)	110 (6.8)	105 (7.4)	109 (7.8)	90 (7.9)	116 (10.0)	90 (10.1)	606 (68.5)

© 2024 Ishani A et al. *JAMA Network Open.*

eFigure 4. Adjusted Subgroup Analysis: Time to Doubling of Serum Creatinine, Terminal eGFR <15 mL/min/1.73m^2, or Dialysis Initiation

Subgroup	Total no. of patients	No. of patients with outcome (%)	HR (95% CI)	Interaction P
eGFR				0.42
eGFR >=60	9,038	452 (5)	0.90 (0.75, 1.09)	
eGFR <60	3,227	313 (10)	1.02 (0.82, 1.27)	
Age				0.02
<=72	7,320	508 (7)	0.83 (0.70, 1.00)	
>72	4,945	257 (5)	1.21 (0.94, 1.55)	
Race				0.87
Non-Black	10,428	617 (6)	0.95 (0.81, 1.12)	
Black	1,837	148 (8)	0.92 (0.67, 1.28)	
Sex				0.66
Female	389	24 (6)	1.14 (0.50, 2.58)	
Male	11,876	741 (6)	0.94 (0.81, 1.09)	
History of diabetes				0.26
No	6,640	253 (4)	1.06 (0.83, 1.37)	
Yes	5,625	512 (9)	0.89 (0.75, 1.07)	
History of MI or stroke				0.76
No	10,936	665 (6)	0.95 (0.82, 1.11)	
Yes	1,329	100 (8)	0.89 (0.60, 1.33)	
Baseline SBP				0.06
<=136 mmHg	5,543	306 (6)	0.79 (0.63, 0.99)	
>136 mmHg	6,722	459 (7)	1.05 (0.87, 1.26)	

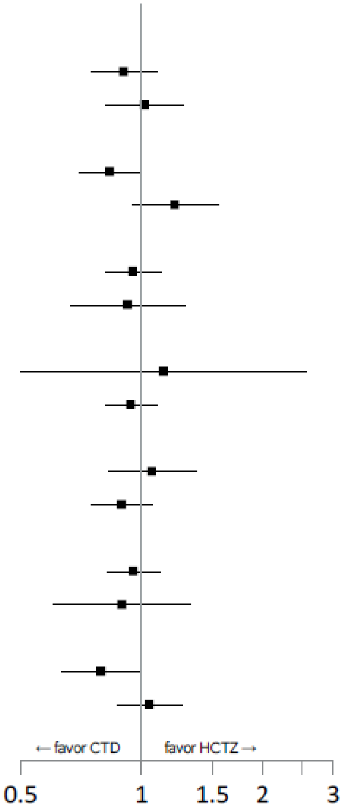

© 2024 Ishani A et al. *JAMA Network Open.*

eFigure 5. Unadjusted Subgroup Analysis: Time to > 40% Reduction in eGFR, Terminal eGFR < 15 mL/min/1.73m^2, or Dialysis Initiation

Subgroup	Total no. of patients	No. of patients with outcome (%)	HR (95% CI)	Interaction P
eGFR				0.39
eGFR >=60	9,038	1059 (12)	0.99 (0.88, 1.12)	
eGFR <60	3,227	537 (17)	0.90 (0.76, 1.07)	
Age				0.01
<=72	7,320	1007 (14)	0.87 (0.77, 0.99)	
>72	4,945	589 (12)	1.13 (0.96, 1.32)	
Race				0.10
Non-Black	10,428	1304 (13)	0.92 (0.83, 1.03)	
Black	1,837	292 (16)	1.14 (0.91, 1.44)	
Sex				0.47
Female	389	45 (12)	1.19 (0.66, 2.14)	
Male	11,876	1551 (13)	0.95 (0.86, 1.05)	
History of diabetes				0.55
No	6,640	552 (8)	1.01 (0.85, 1.19)	
Yes	5,625	1044 (19)	0.95 (0.84, 1.07)	
History of MI or stroke				0.40
No	10,936	1388 (13)	0.97 (0.87, 1.08)	
Yes	1,329	208 (16)	0.86 (0.65, 1.13)	
Baseline SBP				0.53
<=136 mmHg	5,543	631 (11)	0.92 (0.79, 1.08)	
>136 mmHg	6,722	965 (14)	0.98 (0.87, 1.12)	

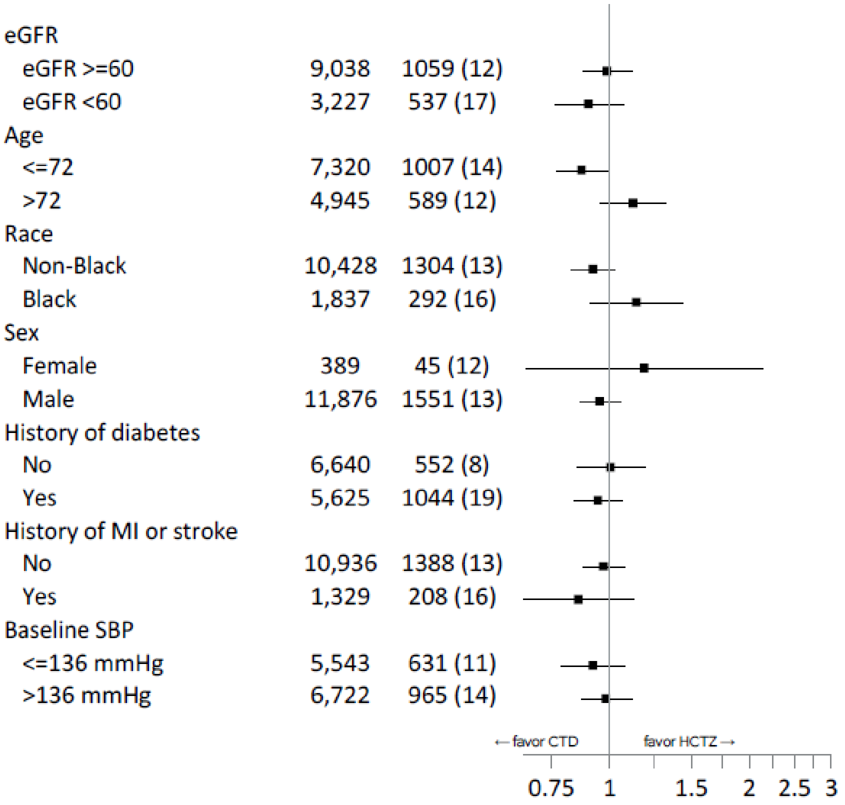

© 2024 Ishani A et al. *JAMA Network Open.*

eTable 4. Safety Outcomes by Treatment and eGFR Level

	Baseline eGFR >=60 (N=9,038)			Baseline eGFR <60 (N=3,227)		
	CTD (n = 4,520)	HCTZ (n = 4,518)	p-value[a]	CTD (n = 1,598)	HCTZ (n = 1,629)	p-value[a]
**Safety outcomes**						
Hospitalization for acute kidney injury	229 (5.1)	205 (4.5)	0.26	162 (10.1)	174 (10.7)	0.64
Hypokalemia	416 (9.2)	315 (7.0)	<0.001	129 (8.1)	111 (6.8)	0.18
Primary cause of hospitalization	161 (3.6)	123 (2.7)	0.03	52 (3.3)	55 (3.4)	0.92
Potassium lab <3.1 mEq/L	305 (6.8)	226 (5.0)	<0.001	95 (5.9)	67 (4.1)	0.02

[a] Computed by Fisher exact test.

© 2024 Ishani A et al. *JAMA Network Open.*
