# Supplementary material for: Chlorthalidone vs Hydrochlorothiazide and Kidney Outcomes in Patients With Hypertension: A Secondary Analysis of a Randomized Clinical Trial
Source: JAMA Netw Open. 2024 Dec 10;7(12):e2449576. doi: 10.1001/jamanetworkopen.2024.49576 (PMC11632543; doi:10.1001/jamanetworkopen.2024.49576)
Supplement: Supplement 3. — Data Sharing Statement [file jamanetwopen-e2449576-s003.pdf]

Data Sharing Statement

Ishani. Chlorthalidone vs Hydrochlorothiazide and Kidney Outcomes in Patients With Hypertension. *JAMA Netw Open*. Published December 10, 2024.
doi:10.1001/jamanetworkopen.2024.49576

Data
Additional Information: ClinicalTrials.gov number, NCT02185417
Data available: No

Additional Information
Explanation for why data not available: Outcomes are from clinical datasets which cannot be shared
